# Non-alcoholic fatty liver disease promotes breast cancer progression through upregulated hepatic fibroblast growth factor 21

**DOI:** 10.1038/s41419-023-06386-8

**Published:** 2024-01-18

**Authors:** Yue Sui, Qingqing Liu, Cong Xu, Kumar Ganesan, Zhen Ye, Yan Li, Jianmin Wu, Bing Du, Fei Gao, Cailu Song, Jianping Chen

**Affiliations:** 1 School of Chinese Medicine, The University of Hong Kong, Pokfulam, Hong Kong China; 2 Chengdu University of Traditional Chinese Medicine, 611137 Chengdu, China; 3 Xiamen University, 361005 Xiamen, China; 4 School of Pharmacy, Southwest Medical University, 646000 Luzhou, China; 5 South China Agricultural University, 510000 Guangzhou, China; 6 Sun Yat-Sen University Cancer Center, 510000 Guangzhou, China; 7 Shenzhen Institute of Research and Innovation, The University of Hong Kong, 518000 Shenzhen, China

**Keywords:** Breast cancer, Tumour biomarkers

## Abstract

Non-alcoholic fatty liver disease (NAFLD) has been shown to influence breast cancer progression, but the underlying mechanisms remain unclear. In this study, we investigated the impact of NAFLD on breast cancer tumor growth and cell viability through the potential mediator, hepatic fibroblast growth factor 21 (FGF21). Both peritumoral and systemic administration of FGF21 promoted breast cancer tumor growth, while FGF21 knockout attenuated the tumor-promoting effects of the high-fat diet. Mechanistically, exogenous FGF21 treatment enhanced the anti-apoptotic ability of breast cancer cells through STAT3 and Akt/FoXO1 signaling pathways, and mitigated doxorubicin-induced cell death. Furthermore, we observed overexpression of FGF21 in tumor tissues from breast cancer patients, which was associated with poor prognosis. These findings suggest a novel role for FGF21 as an upregulated mediator in the context of NAFLD, promoting breast cancer development and highlighting its potential as a therapeutic target for cancer treatment.

## Introduction

Breast cancer is the most commonly diagnosed cancer and the leading cause of cancer-related deaths among women worldwide [1]. Its initiation and progression are complex and influenced by various factors that are still under investigation. Non-alcoholic fatty liver disease (NAFLD), the most prevalent liver disease worldwide [2], is considered a risk factor for breast cancer. It is associated with a rapid increase in incidence rates and worse outcomes [3]. However, current research has mainly focused on epidemiology, while the direct effects and underlying mechanisms of NAFLD on breast cancer remain poorly investigated. One of the obstacles is the co-concurrence of complex epidemics, such as obesity, metabolic syndromes, and type 2 diabetes mellitus, which are also implicated in breast cancer development and obscure the specific hepatic contribution [4]. Notably, NAFLD is commonly observed in individuals with obesity [5]. Although it may not be necessary or feasible to completely separate these two diseases and their influence on breast cancer, liver dysfunction resulting from NAFLD leads to systemic metabolic dysfunction, including exposure to cancer-related sex hormones and rewiring of nutrition [6, 7]. This suggests that the significance of NAFLD in breast cancer may be underestimated.

The crosstalk between distant organs relies on the circulatory system, and as a gland, the liver interacts with other organs and tissues partly through the production of hepatokines [8]. Fibroblast growth factor 21 (FGF21) was identified in 2000 [9]. As a hepatokine, FGF21 is primarily secreted from the liver and exerts its effects on extrahepatic organs or tissues [10]. Over the past two decades, FGF21 has been extensively studied for its role in mediating lipid metabolism and maintaining energy homeostasis [11]. Circulating levels of FGF21 are elevated in obesity and NAFLD [12, 13]. Accumulating studies have shown that circulating FGF21 levels are induced by high-fat diets and positively correlated with the stage of NAFLD, indicating that blood FGF21 levels are highly sensitive in reflecting NAFLD [13]. Furthermore, overexpression of FGF21 has been observed in various cancers including liver, thyroid, and lung cancer [10]. Mechanistically, FGF21 is involved in multiple processes of tumor progression through specific signaling pathways that depend on the type of cancer and the source of FGF21. For example, exogenous FGF21 promotes thyroid cancer cell migration and invasion by upregulating FGFR-EMT signaling [14], while overexpressed FGF21 protects against oxidative stress in lung cancer through the Sirtuin 1/PI3K/AKT signaling pathway [15].

Limited data are available regarding the role of FGF21 in breast cancer. Previous studies have shown higher serum levels of FGF21 in the early stages of breast cancer, but these levels decreased in patients treated with hormones [16, 17]. However, the expression patterns of FGF21 in breast tumor tissues remain unclear, and the impact of FGF21 on tumor growth and progression has not been reported. In this study, we demonstrate that FGF21 is overexpressed in NAFLD models and promotes breast cancer tumor growth while enhancing cell anti-apoptosis abilities. These findings suggest that FGF21 may serve as a potential missing link in the NAFLD–breast cancer axis. Additionally, we provide the first report on the expression profile and prognostic value of FGF21 in breast cancer patients.

## Results

### NAFLD is associated with mammary tumor growth

**Fig. 1:**
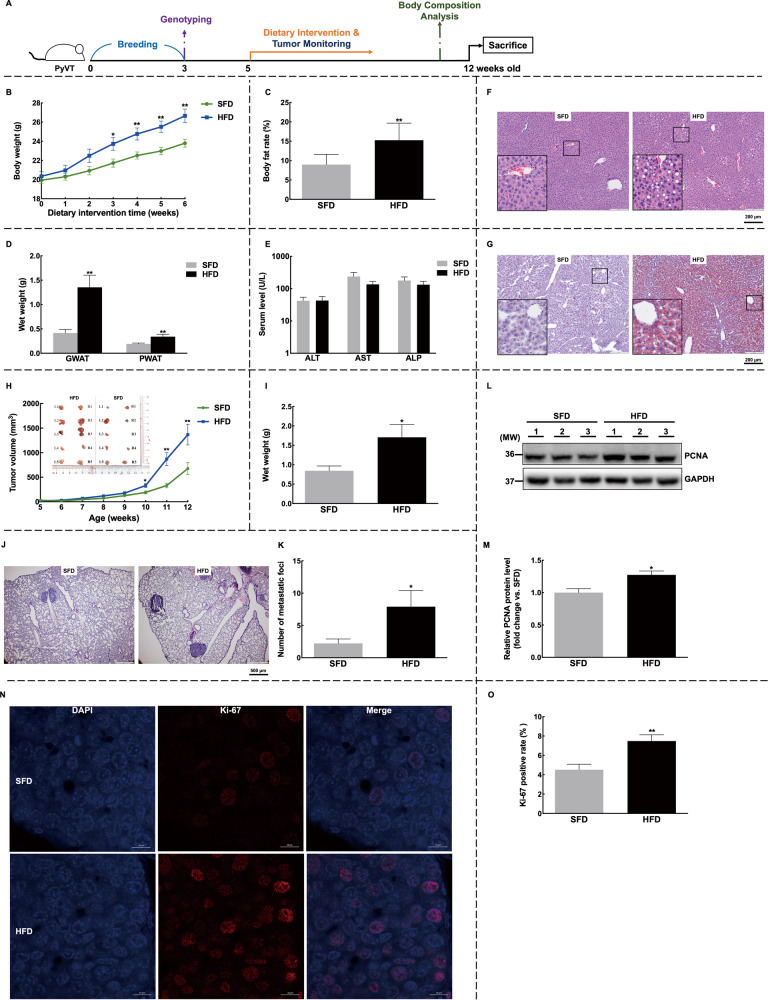
HFD induces NAFLD and promotes the development of mammary tumors in PyVT mice. **A** Trial schematic for establishing HFD-fed NAFLD-breast cancer mice model. Briefly, hemizygous female MMTV-PyMT mice were bred for 3 weeks and genotyped. Mice were randomly divided into the HFD and SFD groups. Weekly measurements included body weight, food intake, and tumor volume with body composition tested before sacrifice. **B**, **C** Mice on the HFD showed accelerated body weight growth (**B**) and higher body fat rates (**C**). **D** Increased weights of adipose tissue were observed in both posterior and gonadal white adipose tissues. **E**–**G** Evaluation of NAFLD revealed that the HFD did not induce significant changes in liver enzymes (**E**) but stimulated lipid droplet accumulation in the liver tissues, as observed through H&E staining (**F**) and oil red O staining (**G**); Images were shown at ×4 magnification, scale bars of 200 μm, and insert images were at ×20 magnification. **H**–**O** For tumors, mice on the HFD showed accelerated mammary tumor growth (**H**) and higher tumor wet weight (**I**); **J**, **K** Lung metastasis was evaluated by H&E staining (**J**), and the number of metastasis foci was quantified (**K**). **L**, **M** The expression levels of PCNA were confirmed by western blot (**L**) and quantified (**M**). **N**, **O** Immunofluorescence staining of Ki67 was performed on tumor slices (**N**) and the percentage of Ki67-positive cells was quantified (**O**). Representative images were shown at ×40 magnification, and scale bars were 10 μm. *n* = 15 for each group; Data were expressed as mean ± SD. The difference between groups was assessed using Student’s *t-*test, *p < 0.05, **p < 0.01. HFD high-fat diet group, SFD standard-food diet group, PWAT posterior white adipose tissue, GWAT gonadal white adipose tissue.

To investigate the relationship between NAFLD and breast cancer progression, we used a high-fat diet (HFD) to establish the NAFLD model in female MMTV-PyMT mice. This model closely resembles human NAFLD and induces the metabolic comorbidities commonly observed in humans with NAFLD [18]. The mice were fed with HFD from 5 weeks old and continued for 7 weeks to establish a breast cancer model with NAFLD (Fig. 1A). Our results showed that the HFD led to increased body weight (Fig. 1B) and body fat percentage (Fig. 1C), as well as excessive accumulation of subcutaneous and visceral fat (Fig. 1D). Although liver enzyme levels did not exhibit significant changes (Fig. 1E), the presence of lipid droplet accumulation confirmed the fatty liver characteristics (Fig. 1F and G). Evaluation of tumors in 10 mammary glands per mouse revealed that while there was no difference in tumor latency between the two groups (data not shown), mice in the HFD group showed faster tumor growth, as evidenced by increased tumor volume and weight (Fig. 1H and  I). Additionally, we observed a higher metastatic burden in the lungs of mice in the HFD group, indicating an increased propensity for mammary tumors to metastasize (Fig. 1J and K). Furthermore, higher expression levels of PCNA (Fig. 1L and M) and Ki67 (Fig. 1N and O) indicated enhanced cell proliferative activity in the tumors of the HFD group. Taken together, our findings demonstrated that HFD-induced NAFLD was observed in MMTV-PyMT mice, which was accompanied by accelerated breast cancer growth.

**Fig. 2:**
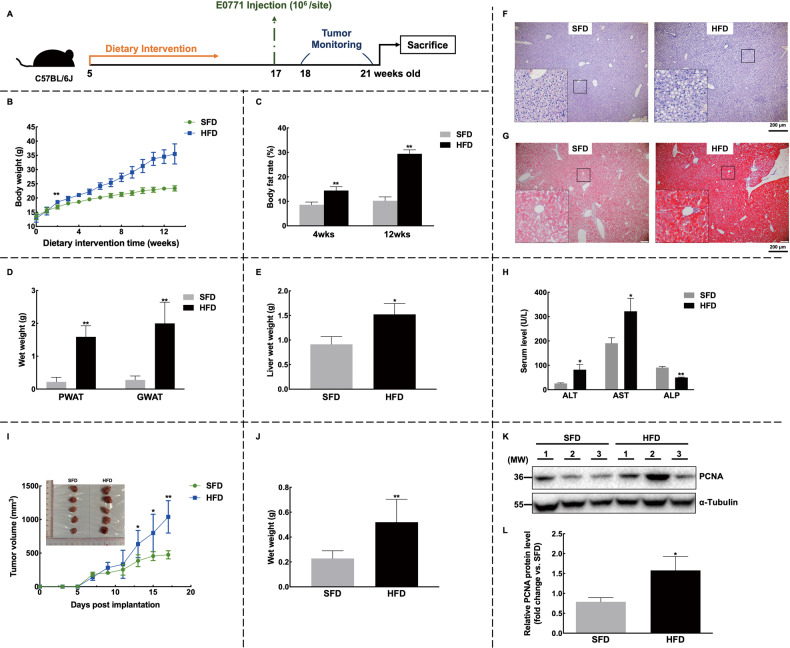
HFD induces NAFLD and promotes the development of mammary tumors in C57BL/6J mice. **A** Trial schematic for establishing an HFD-fed NAFLD-breast cancer mice model. **B**, **C** Mice on the HFD diet showed accelerated body weight growth (**B**) and higher body fat rates (**C**). **D** Increased wet weights of adipose tissue were observed. **E**–**H** Evaluation of NAFLD revealed that the HFD induced higher liver wet weight (**E**) and aberrant expression of liver enzymes (**H**). Lipid droplet accumulation in the liver tissue was detected by H&E (**F**) and oil red O (**G**) staining; Images were shown at ×4 magnification, with scale bars of 200 μm, and insert images were at ×20 magnification. **I**–**L** For tumors, mice fed with HFD showed accelerated mammary tumor growth (**I**) and higher tumor wet weight (**J**). The expression levels of PCNA were confirmed by western blot (**K**) and quantified (**L**). *n* = 10 for each group; Data were expressed as mean ± SD. The difference between groups was assessed by Student’s *t-*test, *p < 0.05, **p < 0.01. HFD high-fat diet group, SFD standard-food diet group, PWAT posterior white adipose tissue, GWAT gonadal white adipose tissue.

Given that MMTV-PyMT mice are not responsive to HFD and develop tumors spontaneously [19], we established another NAFLD-breast cancer model with C57BL/6J background (Fig. 2A). As expected, mice that were fed with HFD for 12 weeks showed typical fatty liver characteristics, including increased liver weight (Fig. 2E) and hepatic steatosis (Fig. 2F and G). The altered levels of ALT, AST, and ALP further indicated aberrant liver function (Fig. 2H). Similarly, we observed faster tumor growth in the E0771 breast cancer model (Fig. 2I–L).

**Fig. 3:**
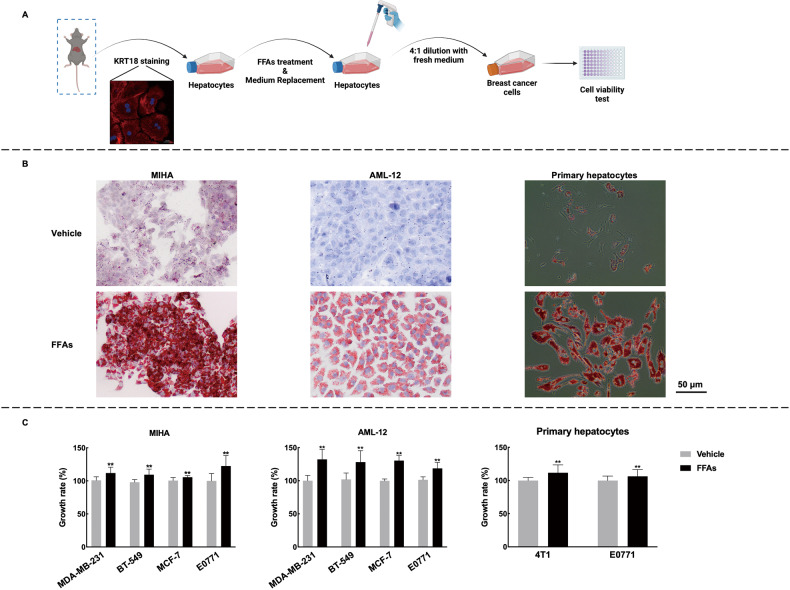
Conditioned media from hepatocytes promote cell viability of breast cancer cell lines. **A** Trial schematic for establishing an in vitro NAFLD model. Briefly, primary hepatocytes were isolated from C57BL/6J mice and identified with immunofluorescence staining of KRT18. To establish the NAFLD model, hepatocytes were treated with FFAs for 24 h and then cultured with serum-free medium for an additional 12 h. The conditioned medium was collected, filtered, and diluted to 25% with fresh culture medium before being used to treat breast cancer cell lines. **B** The presence of lipid droplets in hepatocytes was detected by oil-red O staining. The representative images were shown at ×20 magnification, and the scale bars were 50 μm. **C** Cell viability was assessed using the MTS assay. The results suggested that conditioned medium from hepatocytes promoted breast cancer cell growth. *n* = 5 for each group; Data were expressed as mean ± SD. The difference between groups was assessed by Student’s *t-*test, * *p* < 0.05, **p < 0.01. FFAs: 1 mM palmitic acid combined with 0.25 mM oleic acid. The trial schematic was created with BioRender.com.

High-fat diet-induced NAFLD often leads to metabolic disorders such as excessive fat accumulation (Fig. 2B–D), which can hinder our ability to directly investigate the effects of the liver on breast cancer. To address this limitation, we established an in vitro NAFLD model using a conditioned medium (Fig. 3A). Hepatocytes were treated with combined free fatty acids (FFAs), and the abundance of lipid droplets was detected by oil red O staining (Fig. 3B). We observed a modest but significant increase in breast cancer cell viability when treated with conditioned medium from FFAs-treated hepatocytes (Fig. 3C), indicating that NAFLD promotes breast cancer growth.

### Hepatic FGF21 is overexpressed in the NAFLD model

**Fig. 4:**
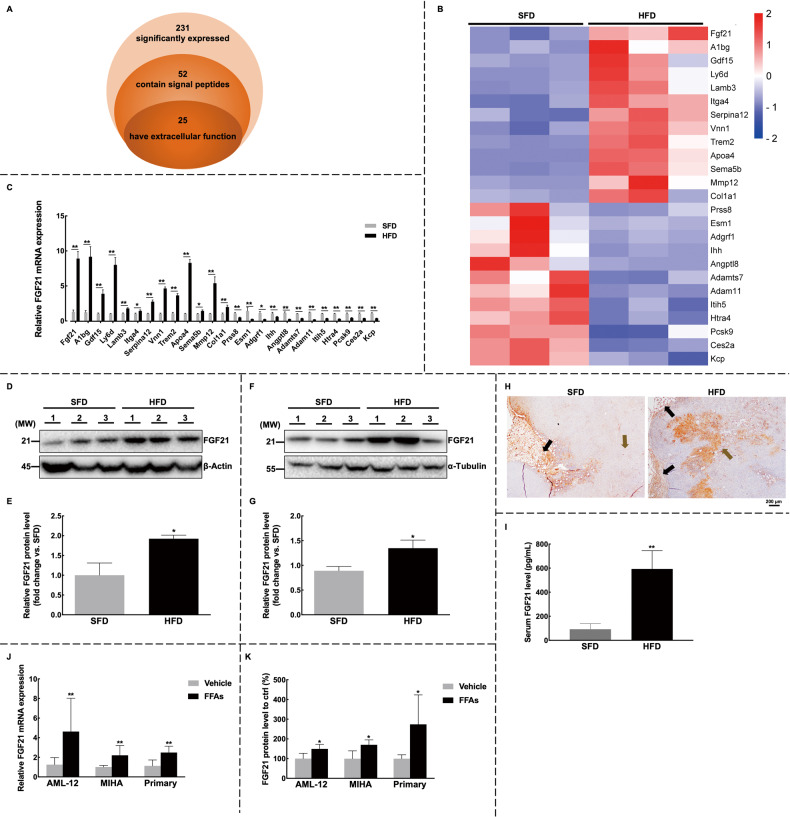
FGF21 is found to be over-expressed in the NAFLD-breast cancer model. **A**–**C** The significantly changed secretory proteins in the livers of HFD-fed C57BL/6J mice were investigated. **A** A total of 231 genes were sequenced using mRNA sequencing and found to be differentially expressed between the groups. Among these, 52 genes were identified to have the secretory signal peptides using SignalP 6.0. Their functional information was further evaluated by UniProt and GeneCards. **B** Finally, 25 genes with potential extracellular functions were identified as differentially expressed in NAFLD livers. **C** The expression levels of these 25 genes were further confirmed by RT-qPCR. The experiments were conducted with *n* = 3 for each group in (**A**–**C**). **D**, **E** The expression of FGF21 in the livers was evaluated by western blot (**D**) and quantified (**E**). **F**, **G** The expression of FGF21 in tumors was evaluated by western blot (**F**) and quantified (**G**). **H** The expression pattern of FGF21 in tumor tissues was analyzed by immunohistochemistry staining. Images were shown at ×4 magnification, scale bars 200 μm. Black arrow: peritumor area; Brown arrow: tumor area. **J**, **K** The expression levels of FGF21 were tested in immortalized hepatic cell lines MIHA and AML-12, as well as primary hepatocytes. **J** The mRNA expression levels of FGF21 were elevated in the conditioned media of FFAs-treated hepatocytes. **K** The protein levels of FGF21 were elevated in the conditioned media of FFAs-treated hepatocytes. The experiments were conducted with *n* = 3 for each group in (**J** and **K**). **I** The increased serum FGF21 levels in C57BL/6J mice were detected using an ELISA kit, with *n* = 6 for each group. Data were expressed as mean ± SD. The difference between groups was assessed by Student’s *t-*test, *p < 0.05, **p < 0.01. HFD high-fat diet group, SFD standard-food diet group, FFAs 1 mM palmitic acid combined with 0.25 mM oleic acid.

To investigate the possible oncogenic mechanisms of NAFLD, we performed RNA sequencing analysis on liver tissues from C57BL/6J mice with mammary tumors fed either a standard food diet (SFD) or HFD. Our analysis revealed significant changes in the expression of 231 genes in the livers of mice following HFD intervention, out of which 52 genes showed the potential to encode secretory proteins (Fig. 4A). Based on the functional information and gene expression levels, we further narrowed down the selection to 25 genes that exhibited secretory capacity or extracellular activities (Fig. 4B and C). These findings shed light on the altered secretory system of the liver under conditions of NAFLD.

The expression of FGF21, a hepatokine, was significantly increased in the liver of mice in the HFD group (Figs. 4D and E, S1A–C). Moreover, FGF21 levels were consistently elevated in the serum and conditioned medium of NAFLD models (Figs. 4I–K, S1D). We also evaluated the distribution of FGF21 in tumors (Fig. 4H). Interestingly, compared to adjacent adipose tissues, tumor tissues in the SFD group showed minimal expression of FGF21, but there was a marked increase in the HFD group, as confirmed by western blot analysis (Fig. 4F and G). A similar trend was observed in PyVT mice, although the enrichment of FGF21 was more apparent in the peritumoral areas, with both adipose tissue and the core tumor area showing low expression of FGF21 (Fig. S1E–S1G). These findings suggest that increased hepatic FGF21 levels in NAFLD may circulate to breast tumors and influence tumor growth.

### In vivo FGF21 administration promotes breast cancer tumor growth

**Fig. 5:**
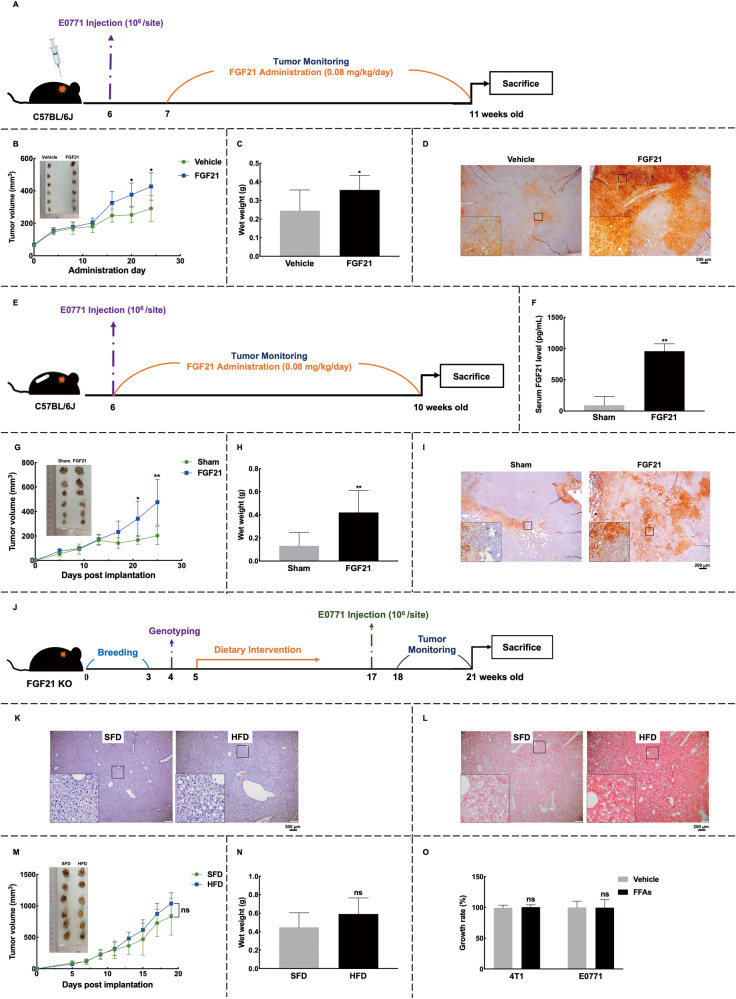
Administration of recombinant FGF21 promotes breast cancer tumor growth. **A**–**D** FGF21 peritumoral injection model. **A** Trial schematic. **B**, **C** The promoting effects of FGF21 on breast cancer were observed as accelerated tumor growth (**B**) and higher tumor weight (**C**). **D** Tumor sampling was performed 2 h after FGF21 administration was applied to confirm the in-situ enrichment of FGF21. **E**–**I** FGF21 sustained-release model. **E** Trial schematic. **F** The serum levels of FGF21 were measured by ELISA at the endpoint, and mice in the FGF21 group showed comparable serum levels to the HFD-fed mice shown in Fig. 4I. **G**, **H** Mice with FGF21 supplementation showed faster tumor growth (**G**) and higher tumor weight (**H**). **I** The expression levels of tumoral FGF21 were increased in the treatment group. **J**–**O** FGF21 knockout model. **J** Trial schematic. **K**, **L** Liver tissue exhibited over-accumulation of lipid droplets with HFD (**K**), stained red with oil red O (**L**). **M**, **N** Mice on the HFD showed comparable tumor growth curves (**M**) and tumor weights (**N**) to mice in the SFD group. **O** Conditioned medium from FGF21 KO hepatocytes was collected to test the effects on breast cancer cell lines 4T1 and E0771. *n* = 6 for each group. Data were expressed as mean ± SD. The difference between groups was assessed by Student’s *t-*test, *p < 0.05, **p < 0.01, ns *p* > 0.05. Images were shown at ×4 magnification, with scale bars 200 μm, and inset images at ×20 magnification. HFD high-fat diet group, SFD standard-food diet group, FFAs 1 mM palmitic acid combined with 0.25 mM oleic acid.

To study the effects of elevated systemic or hepatic FGF21 on breast cancer, we utilized an allograft breast cancer animal model by transplanting E0771 cells into C57BL/6J mice, followed by peritumoral injection of recombinant mouse FGF21 (Fig. 5A). Immunohistochemistry (IHC) staining of tumor samples collected after 2 h of FGF21 administration confirmed the in-situ enrichment of FGF21 (Fig. 5D). Importantly, we observed that FGF21 treatment over a period of 3 weeks significantly accelerated E0771 tumor growth, as evidenced by increased tumor volume and wet weight (Fig. 5B and C).

The hepatic FGF21 can reach tumor tissues via the circulating system. To assess the effects of circulating FGF21 on breast cancer, osmotic pumps containing FGF21 were used to mimic the levels of circulating FGF21 detected in NAFLD models (Fig. 5E and F). Consistent with this, we observed a higher intratumoral density of FGF21 in the FGF21 treatment group (Fig. 5I), which corresponded with faster tumor growth and higher tumor weight (Fig. 5G and H). These findings suggest that the increased circulating FGF21 in NAFLD can be enriched in breast tumor tissues and promote breast cancer progression.

To further investigate the role of FGF21 in NAFLD-induced tumor growth, we used FGF21 knockout mice (Fig. 5J). The results showed that despite the presence of severe NAFLD status (Fig. 5K and L), FGF21 knockout diminished HFD-induced breast cancer tumor growth, resulting in comparable tumor volume and weight to the SFD group (Fig. 5M and N). Furthermore, in an in vitro NAFLD model using FGF21 KO hepatocytes, the promoting effects on breast cancer cell viability were lost (Fig. 5O). These findings collectively demonstrate that FGF21 promotes mammary tumor growth, and is implicated in NAFLD-related breast cancer development.

### FGF21 promotes breast cancer cell viability and inhibits doxorubicin-induced cell apoptosis

**Fig. 6:**
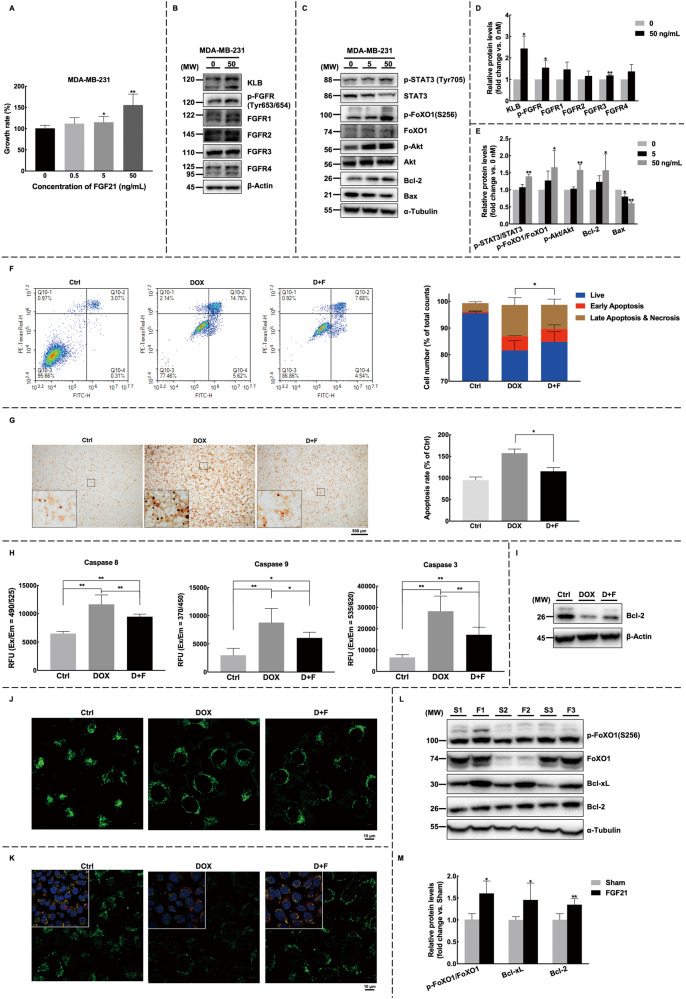
Recombinant FGF21 enhances the anti-apoptotic capability of breast cancer cells through STAT3 and Akt/FoXO1 pathways. **A** MDA-MB-231 cells were exposed to recombinant FGF21 at different concentrations (0, 0.5, 5, 50 ng/mL) for 24 h prior to the MTS assay. **B**–**E** The treatment with FGF21 activated FGF receptors and anti-apoptotic pathways, as indicated by the quantification of the blots. **F**–**I** The antagonistic effects of FGF21 towards doxorubicin were examined. MDA-MB-231 cells were treated with 50 ng/mL FGF21 and 1.25 μM DOX for 24 h. **F** Cell apoptosis was evaluated using Annexin V assay, with representative images shown (left) and quantified (right). **G** TUNEL assay was also performed to evaluate cell apoptosis with representative images shown (left) and quantified (right). Images were shown at ×4 magnification, scale bars 500 μm, and insert images were at ×20 magnification. The difference in late apoptosis between DOX and D + F groups was marked. **H**, **I** The expression levels of apoptosis-related proteins were determined by the caspase activity test (**H**) and western blot (**I**). **J**, **K** FGF21 was found to protect mitochondria. **J** Mitochondrial membrane potential was evaluated using JC-10, and the fluorescence intensity in live cells was detected by confocal microscopy. **K** The intra-mitochondrial cytochrome c was detected by immunofluorescence. Cytochrome c was stained with anti-cytochrome c antibody in green, the nucleus was counterstained with DAPI in blue, and the mitochondrion was stained with mitotracker in red. Images for **J** and **K** were shown at ×40 magnification, scale bars 10 μm. **L**, **M** The activation of apoptosis pathways was detected in the FGF21 sustained-release model. Graphs represent the quantification of the blots. Data were expressed as mean ± SD. The difference between groups was assessed by Student’s *t-*test or One-way ANOVA combined with Turkey’s test for multiple comparison tests, *p < 0.05, **p < 0.01 com*p*ared to control groups unless otherwise stated. Ctrl control group, DOX doxorubicin treatment group, D + F doxorubicin plus FGF21 treatment group, S sham group, F FGF21 treatment group.

To investigate the molecular mechanisms underlying the tumor-promoting effects of FGF21, breast cancer cell lines were directly treated with recombinant FGF21. FGF21 acts through its obligatory receptors FGFR and co-receptor β-Klotho [20], which were found to be expressed and responsive to FGF21 treatment in breast cancer cell lines (Figs. 6B and D, S2B and S2D). Functionally, FGF21 treatment resulted in varying degrees of increased cell viability, as measured by MTS assay (Figs. 6A and S2A). Furthermore, FGF21 treatment effectively activated the phosphorylation levels of STAT3, Akt, and FoXO1, increased the expression of the anti-apoptotic Bcl-2, and decreased the activity of the pro-apoptotic protein Bax (Figs. 6C and E, S2C and S2E). These findings indicate that FGF21 treatment may enhance the anti-apoptotic ability of breast cancer cells through STAT3 and Akt/FoXO1 pathways.

Given that cancer cells are resistant to apoptosis, we conducted further experiments to confirm the anti-apoptotic effects of FGF21. We used the chemotherapy drug doxorubicin (DOX) as an apoptosis inducer [21] and observed antagonistic interactions between DOX and FGF21 on tumor cell viability (Fig. S2F). As expected, both the Annexin V assay (Figs. 6F and S2G) and the TUNEL assay (Figs. 6G and S2H) confirmed that simultaneous administration of FGF21 effectively mitigated DOX-induced breast cancer cell apoptosis. The caspase family activity test indicated that FGF21 inhibited DOX-induced breast cancer cell apoptosis via both extrinsic and intrinsic pathways (Figs. 6H and S2I). These results were further confirmed by the consistently activated anti-apoptosis signaling pathways in both cell levels (Figs. 6I, S2L and S2M) and tumor levels (Figs. 6L and M, S2J and S2K).

During apoptosis induced by chemotherapeutic agents, mitochondria permeabilization occurs [22]. In our study, we detected a decreased mitochondrial membrane potential, as indicated by the increased monomeric form of JC-10 dye in breast cancer cells treated with DOX. However, the decrease was mitigated when FGF21 treatment was added (Figs. 6J and S3A). Additionally, cytochrome c is known to be released into the cytosol during apoptosis, where it activates the caspase cascade [23]. To evaluate the release of cytochrome c, we selectively permeabilized the plasma membrane using digitonin and visualized the expression of intra-mitochondrial cytochrome c through immunofluorescence. As expected, cytochrome c was released from the mitochondria in the DOX treatment group, resulting in faint intracellular fluorescence. However, the FGF21 supplement protected cells from cytochrome c translocation (Figs. 6K and S3B).

In conclusion, our findings suggest that FGF21 promotes the anti-apoptotic ability of breast cancer cells via Akt and STAT3 pathways.

### Clinical relevance of FGF21 in breast cancer

**Fig. 7:**
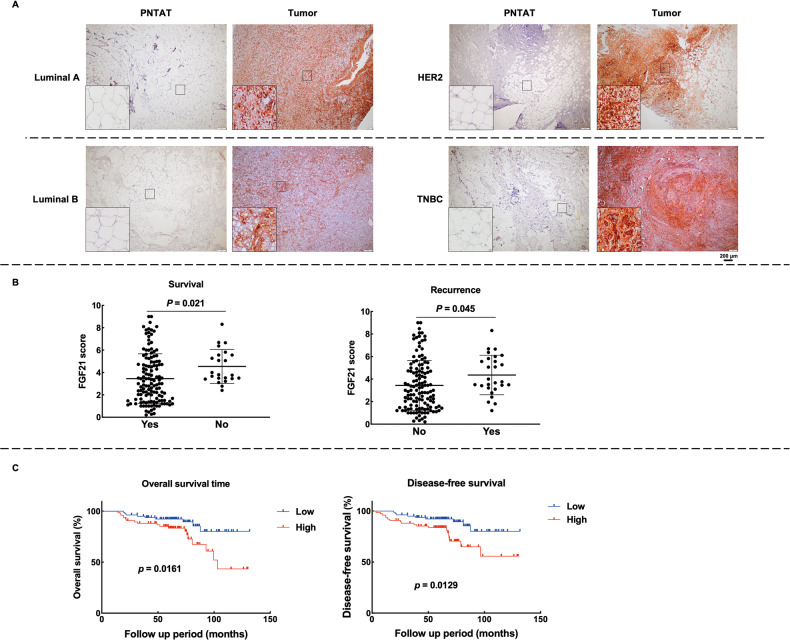
FGF21 is overexpressed in breast cancer tissue and correlates with prognosis. IHC staining was utilized to investigate the relationship between FGF21 expression levels and clinical pathological characteristics. **A** Tumor samples from patients with different molecular types of breast cancer were collected and subjected to staining with anti-FGF21 antibodies. Images were shown at ×4 magnification, with scale bars 200 μm, and insert images at ×20 magnification. **B**, **C** A tissue microarray consisting of tumors from 157 TNBC patients was analyzed. **B** Overexpression of FGF21 was observed in subjects with recurrent and deceased patients. The difference between groups was assessed by Student’s *t-*test, and the mean with SD was presented. **C** Patients were categorized into low and high-expression groups based on FGF21 levels. The disease-free survival rate and overall survival between these groups were analyzed using the Kaplan–Meier tool, and differences were analyzed by log-rank test. PNTAT paired normal tissues adjacent to the tumor.

To assess the clinical relevance of FGF21 in breast cancer, we analyzed the expression of FGF21 in different molecular types of tumor samples along with paired adjacent normal tissues (PNTAT). Interestingly, while minimal positive staining was observed in PNTAT, FGF21 was generally expressed in tumor tissues regardless of the molecular type (Fig. 7A). To further investigate the association between FGF21 expression and breast cancer prognosis, we grouped 157 breast cancer patients into low or high-FGF21 expression subsets. Consistent with the tumor-promoting effects observed in mice and cell lines, patients with high expression levels of FGF21 showed significantly shorter overall survival time and shorter disease-free survival (Fig. 7C). Moreover, patients in the high FGF21 expression groups had lower survival rates and higher recurrence rates (Table 1, Fig. 7B). These findings suggest the potential of intratumoral FGF21 as a biomarker for breast cancer diagnosis and prognosis.

**Table 1:** Clinicopathological parameters of 157 patients according to intratumoral levels of FGF21.

Variables		Groups	Low FGF21 group (*n* = 81)	High FGF21 group (*n* = 76)	*p* Value
Age (years)		<45	36	36	0.327
		≥45	45	40	
Menopause		No	52	29	0.418
		Yes	44	32	
TNM Staging	T stage	T1–T2	76	68	0.637
		T3–T4	5	8	
	Lymph node metastasis	No	38	39	0.581
		Yes	43	37	
	Distant metastasis	No	80	75	0.964
		Yes	1	1	
	TNM staging	T1–T2	52	50	0.863
		T3–T4	29	26	
Recurrence		No	72	57	0.023*
		Yes	9	19	
Survival		No	7	17	0.017*
		Yes	74	59	
Ki67		Low	51	52	0.247
		High	27	16	

The difference between groups was tested by either Chi-squared test or Ridit analysis.

*p < 0.05.

## Discussion

Although NAFLD has been established as an independent risk factor for breast cancer, its exact contributions are still unclear. Instead, obesity, which is closely associated with NAFLD and an important cause of the disease, has garnered significant attention in breast cancer research due to its well-established oncogenic mechanisms [24]. Obesity is often induced through an obesogenic diet, which also is the most common method to establish an obesity model, but it also induces hepatic steatosis [25]. Current studies have primarily focused on the contribution of adipose tissue in tumor-adjacent areas, neglecting the impact of systemic factors or other implicated organs on breast tumor progression [26]. Significantly, adipose tissue-generated adipokines have been shown to correlate with tumor initiation, progression, and recurrence [27]. However, the role of hepatokines, another group of hormones aberrantly expressed in similar contexts, in breast cancer is poorly understood. To explore the undefined mechanism underlying NAFLD and obesity-related breast cancer progression, our study targeted liver tissue and identified significantly overexpressed hepatokines, with FGF21 being one of them. Importantly, FGF21 is not expressed or released in human adipose tissue; Instead, hepatic FGF21 knockout in mice resulted in undetectable levels of circulating FGF21 and impaired glucose metabolism [28, 29], highlighting the dominant role of the liver in mediating the systemic concentration and activities of FGF21. In addition, FGF21 resistance is observed in metabolic diseases including NAFLD [30], suggesting that aberrant expression in these scenarios may endow FGF21 with aberrant activities, such as the tumor-promoting effect observed in our study.

We previously discussed the potential role of FGF21 in cancers [10]. However, the effects of endocrine FGF21 in cancers, as well as the underlying molecular mechanisms, remain largely unknown. To determine the source of FGF21 and its impact on breast cancer, we established an in vitro conditioned medium-based system. This allowed us to study the influence of secretory substances from hepatocytes on breast cancer cell viability. Additionally, we used primary hepatocytes from both wild-type and FGF21 knockout mice to emphasize the importance of FGF21 in the NAFLD–breast cancer axis. Furthermore, to mimic the elevated circulating levels of FGF21 induced by HFD, we employed an osmotic pump. Interestingly, we observed comparable tumor-promoting effects with peritumoral FGF21 administration, suggesting the significant impact of pathological levels of systemic FGF21 on breast cancer. It is important to note that the concentrations of FGF21 administrated in our study were intended to mimic levels observed in HFD-induced NAFLD or obesity. These concentrations were much lower than the pharmacological levels [31] and were consistent with comparable body weight between treatment and control groups (data were not shown). On the other hand, the increased tumor growth observed in FGF21 knockout mice suggests that FGF21 is not the sole mediator involved in high-fat diet-induced breast cancer progression.

The biological actions of FGF21 rely on its binding to FGFR facilitated by β-Klotho. Although the preference of FGFR for specific FGFR members is still a subject of debate and may vary in different contexts [32, 33], we detected the general expression of β-Klotho and FGFR in the tested cell lines. This opens up opportunities to investigate the downstream pathways of FGF/FGFR signaling. Previous studies have shown that FGF21 promotes aggressiveness in thyroid tumors by activating EMT signaling, ERK, and Akt pathways [14]. However, in our study, we did not observe significant effects of FGF21 on the migration of breast cancer cell lines (Fig. S4).

Evading apoptosis is a hallmark of cancer, and there is considerable interest in therapies that restore apoptosis signaling pathways to normality [34]. DOX, a widely used chemotherapy agent in breast cancer treatment, has been shown to regulate cell death through both extrinsic and intrinsic apoptosis pathways [21, 35]. Consistent with these findings, we observed damaged plasma membrane integrity (annexin V/PI staining), DNA fragmentation (TUNEL), mitochondrial dysfunction, and activation of the Bcl-2 family and caspases in tumor cells treated with DOX. However, when combined with FGF21, the efficacy of DOX was effectively reduced, indicating the adverse effects of FGF21 on DOX-related chemotherapy. On the other side, the regulation of upstream pathways showed high variability among cell lines.

HFD has been found to decrease the effectiveness of DOX [36], although the underlying mechanism is still unclear. Previous studies have shown that adipokines such as adiponectin can induce cell apoptosis and enhance the effects of DOX in breast cancer [37, 38] while leptin and resistin can decrease cancer cell apoptosis and reduce the efficacy of DOX [39, 40]. In our study, we showed that FGF21 promotes resistance to DOX in mammary tumor cells, indicating that FGF21 may be implicated in the chemoresistance of breast cancer. The implication of NAFLD and the interactions between FGF21 and other secretory proteins in this process warrant further investigation.

Regarding the expression of FGF21 in cancers, it is important to note that while increased serum FGF21 has been widely observed in various cancers, intratumoral FGF21 expression profiles are poorly understood [10]. Notably, FGF21 is found to be overexpressed in liver cancer, and non-small cell lung cancer, decreased in prostate cancer and pancreatic cancer, and lacking in thyroid cancer [10]. This discrepancy indicates the diversity between tumors and the pleiotropic nature of FGF21 activities. In our study, we observed FGF21 aggregation in tumors from the HFD and FGF21 administration groups, suggesting a positive correlation between FGF21 expression and tumor development. Our clinical data further emphasized the overexpression of FGF21 in breast cancer and its prognostic value. Importantly, the expression of FGF21 appears to be uncorrelated to the presentation of estrogen receptor, progesterone receptor, human epidermal growth factor receptor 2, and Ki-67 (Fig. 7, Table 1). This indicates that FGF21 may have broad applicability as a clinical marker in breast cancer. Besides, serum FGF21 levels have been proposed as a sensitive diagnostic marker for early detection of breast cancer, although they are not associated with prognosis [16]. It is recommended to conduct further studies with a large sample size to validate these findings.

In summary, our study provides evidence that the increased expression of hepatic FGF21 in HFD-induced NAFLD promotes the progression of breast cancer by enhancing the anti-apoptotic abilities of breast cancer cells. We have observed overexpression of FGF21 in breast cancer tissues, and patients with high FGF21 levels show poorer prognoses. These findings highlight the clinical significance of FGF21 as both a prognostic indicator and a potential target for the treatment of breast cancer. Moreover, our study emphasizes the importance of maintaining liver health in the prevention and treatment of breast cancer, as it reveals the existence of distant crosstalk between the liver and breast cancer.

## Material and methods

### Cell lines

Cell lines were obtained from ATCC (Virginia, USA) with the exception of MIHA, which was purchased from Yaji Biotechnology Co. (Shanghai, China). The following culture media and supplements were used for different cell lines.

MDA-MB-231, BT-549 and E0771: DMEM (11965126, Gibco, MA, USA) supplemented with 10% FBS (10270106, Gibco) and 1% penicillin/streptomycin (15140122, Gibco).

MCF-7, 4T1 and MIHA: RPMI 1640 (11875135, Gibco) supplemented with 10% FBS and 1% penicillin/streptomycin.

AML-12: DMEM F12 (10565018, Gibco) supplemented with 10% FBS and 1% penicillin/streptomycin.

MCF-10A: DMEM F12 supplemented with 5% horse serum (16050122, Gibco) and 1% penicillin/streptomycin, 20 ng/mL EGF (SRP3027, Sigma, MA, USA), 0.5 μg/mL hydrocortisone (H0888, Sigma), 10 μg/mL insulin (I0516, Sigma), and 0.1 μg/mL cholera toxin (C8052, Sigma).

All cells were cultured in a humidified incubator at 37 °C with 5% CO_2_.

### Animals

Hemizygous female MMTV-PyMT mice were obtained from our in-house breeding colony. The colony was established using FVB/N females and hemizygous FVB/NTg(MMTV-PyVT)634Mul/J males, which were generously gifted by Dr. WANG Yu from the University of Hong Kong. Genotyping of the mice was performed by PCR using primers: (F) 5′-GGAAGCAAGTACTTCACAAGGG-3′; (R) 5′-GGAAAGTCACTAGGAGAGGG-3′. Female FGF21 knockout mice were kindly provided by Dr. XU Aimin [41]. C57BL/6J female mice (000664, The Jackson Laboratory, Maine, USA) were obtained from the Centre for Comparative Medicine Research of the University of Hong Kong.

All mice were housed in standard individually ventilated cages with 12 h light–dark cycle at a temperature of 22–24 °C, a humidity of 60–70%, and ad libitum access to water and food. For both wild-type and FGF21 knockout mice with C57BL/6J background, 5-week-old female mice were randomly divided into two groups: SFD or HFD (D12492, Research Diet, NJ, USA). Body weight and food intake were measured weekly, and body composition was assessed using a body composition analyzer (Minispec LF90, Bruker, MA, USA). After 12 weeks of dietary intervention, 1 × 10^6^ E0771 cells suspended in PBS were injected into the second mammary fat pad of the mice, and tumor volume was regularly measured. Tumor volume was determined using calipers and calculated using the formula 0.5 × long × width^2^ (mm^3^).

### Human breast cancer samples

Clinical samples from a total of 157 patients with primary breast carcinomas were collected from Sun Yat-sen University Cancer Center (Guangzhou, China) following surgical resection. All subjects were Chinese females, with an average age of 47 (ranging from 26 to 77). The first enrollment of patients took place from 9 March 2005 to 6 September 2011. The mean duration of follow-up was 64.5 ± 24.5 months, with a median follow-up period of 63.0 (ranging from 13.8–127.4) months.

### Isolation of hepatocytes

Primary hepatocytes were isolated using a previously published method [42, 43]. Briefly, a 21-week-old female C57BL/6J mouse was euthanized, and 70% ethanol (1.00983, Sigma) was used to saturate the mouse. The inferior vena cava was cannulated to enable in situ liver digestion with collagenase IV (17104019, Gibco). The released cells were filtered using a 70 mm cell strainer (352350, Corning, NY, USA), and the hepatocytes were purified using 45% percoll (40501, YEASEN, Shanghai, China). The hepatocytes were then cultured in a dish with DMEM supplemented with 10% FBS and 1% penicillin/streptomycin. Functional studies were initiated the following day.

### MTS assay

Breast cancer cell lines MDA-MB-231, MCF-7, BT-549, and E0771, as well as the human breast epithelial cell line MCF-10A, were treated with either recombinant human FGF21 (ab238297, Abcam, Cambridge, UK) or mouse FGF21 (42189, IMD, Hong Kong) for a duration of 24 h. Following the treatment, cell viability was assessed using the MTS assay (G3582, Promega, WI, USA). Cell viability was determined by measuring the absorbance at 490 nm using a microplate reader (0430, BMG LABTECH, Ortenberg, Germany).

The potential interaction between DOX (sc-200923, Santa Cruz, TX, USA) and FGF21 was evaluated using an MTS assay. Cells were treated with DOX at specific concentrations (1.25 μM for MDA-MB-231 and E0771, 5 μM for MCF-7) either alone or in combination with 50 ng/mL FGF21. After a 24-h treatment period, the cells were washed with PBS (70011044, Gibco) and subjected to the MTS assay. The interactions between DOX and FGF21 were further analyzed using an online tool [44].

### Western blot

Total protein was extracted using RIPA lysis buffer (20-188, Millipore) supplemented with protease inhibitor (ab201120, Abcam) and quantified using the bicinchoninic acid kit (23225, ThermoFisher). Equivalent amount of proteins was separated using 10% SDS–PAGE and transferred onto PVDF membranes (IPVH00010, Millipore). After blocking with 5% BSA (A3059, Sigma), the membranes were incubated overnight at 4 °C with primary antibodies. The primary antibodies used were PCNA (A0264, Abclonal, Massachusetts, USA), FGF21 (A3908, Abclonal), FGFR1 (A21219, Abclonal), FGFR2 (A19051, Abclonal), FGFR3 (A19052, Abclonal), FGFR4 (A9197, Abclonal), KLB (A15629, Abclonal), p-FGFR (3471S, Cell Signaling Technology), STAT3 (4904, Cell Signaling Technology), p-STAT3 (AP0705, Abclonal), FoXO1 (A2934, Abclonal), p-FoXO1 (AP0172, Abclonal), Akt (ab179463, Abcam), p-Akt (ab192623, Abcam), Bax (A0207, Abclonal), Bcl-2 (A19693, Abclonal), Bcl-xl (A0209, Abclonal), α-Tubulin (AC007, Abclonal), β-Actin (ab8226, Abcam) and GAPDH (AC002, Abclonal). The membranes were then incubated with secondary antibodies (7074, 7076, Cell Signaling Technology, Massachusetts, USA) for 1 h at room temperature. Protein bands were visualized using an HRP substrate (WBLUF0500, Millipore) on a gel imaging system (ChemiDoc XRS+, Bio-Rad, California, USA). The images were analyzed using Image Lab software (version 6.1, Bio-Rad).

### Immunohistochemistry

Tumor tissues were fixed in 4% paraformaldehyde (158127, Sigma), OCT-embedded (14020108926, Leica, TX, USA), and sectioned using a cryostat (CM1950, Leica). Antigen retrieval was performed using citrate buffer (005000, ThermoFisher) and non-specific binding was blocked using 1% goat serum (16210064, Gibco). The slides were then incubated with primary antibodies against FGF21 (diluted 1:100) overnight at 4 °C, followed by incubation with HRP-conjugated secondary antibodies (7074, Cell Signaling Technology) for 1 h at room temperature. The slides were counterstained with hematoxylin (GHS332, Sigma) after the DAB reaction (K3468, Dako, CA, USA), dehydrated and mounted (100579, Sigma). Images were obtained using a light microscope (BX43, OLYMPUS, Tokyo, Japan) and analyzed using cellSens imaging software (OLYMPUS).

Blind evaluation of FGF21 immunostaining was performed in clinical breast cancer tissues. The scoring criteria were based on the staining intensity and percentage of positive cells. Staining intensity was scored as 0 for negative, 1 for weakly positive, 2 for moderately positive, and 3 for strongly positive. The percentage of positive cells was scored as 1 for positive in 20%; 2 for positive in 60%, and 3 for positive in 100%. The IHC score was calculated as staining intensity multiplied by the percentage of positive cells. An IHC score of <3.4 was considered a low expression, while an IHC score of >3.4 was considered a high expression of FGF21 in tumor tissues.

### Immunofluorescence

Tumor tissues were fixed in 4% paraformaldehyde, embedded in OCT, and sectioned using a cryostat. Antigen retrieval was performed using citrate buffer and non-specific binding was blocked with 1% goat serum. The slides were then incubated overnight at 4 °C with primary antibodies against Ki67 (ab15580, Abcam) followed by incubation with Alexa Fluor-conjugated secondary antibodies (ab150062, Abcam) for 1 h at room temperature.

Primary hepatocytes grown on glass coverslips were fixed with 4% paraformaldehyde for 15 min at room temperature, washed with PBS, and permeabilized with 0.1% triton-X100 (T8787, Sigma) for 3 min. After washing with PBS, the cells were blocked with 3% BSA for 30 min at room temperature. Subsequently, the cells were incubated overnight at 4 °C with primary antibodies against cytokeratin 18 (A19778, ABclonal, diluted 1:100), followed by four washes with PBS and incubation with Alexa Fluor-conjugated secondary antibodies (ab150062, Abcam) for 1 h at room temperature.

Slides were counterstained with Hoechst 33342 (H3570, Invitrogen) and mounted with mounting medium (S3023, Dako) before being analyzed using a confocal microscope (LSM980, ZEISS, Jena, Germany). Laser wavelengths of 488 and 639 nm were used to visualize the fluorescence. Image analysis was performed using ZEN software (Blue 3.1, ZEISS). For Ki67 immunofluorescence staining, six tumors from each group were sectioned, and six slides from each tumor were observed under 20x magnification. Six fields were randomly selected from each slide to quantify the average percentage of Ki67-positive cells.

### Histological analysis

For H&E staining, tissues were fixed in 4% paraformaldehyde, embedded in paraffin (76242, Sigma), and sectioned into 5 mm slices. After deparaffinization and rehydration, the sections were stained with hematoxylin and eosin (HT110116, Sigma) and mounted with DPX (100579, Sigma). The resulting images were observed using a light microscope and analyzed using cellSens imaging software. Lung metastasis was evaluated with H&E staining. Metastatic foci were defined as clusters of more than 10 tightly packed tumor cells. The total number of micrometastases was counted.

For oil red O staining of liver tissues, tissues were fixed in 4% paraformaldehyde and cryopreserved with 15% sucrose (S0389, Sigma) at 4 °C. They were then embedded with the OCT compound. The slides were cut into 7 mm sections in the cryostat and dried at 37 °C for 45 min prior to staining.

For oil-red O staining of hepatocytes, the cells were washed with PBS and fixed in 4% paraformaldehyde for 13 min at room temperature. 0.5% oil red O stock solution was prepared by dissolving the oil red O (O0625, Sigma) in isopropanol (34863, Sigma). The stock solution was then diluted in distilled water in a 6:4 ratio to create the working solution. Slides were rinsed with 60% isopropanol for a few seconds and then stained with oil red O working solution for 15 min at room temperature. After staining, the slides were counterstained with hematoxylin and mounted using glycerin jelly. The glycerin jelly was prepared by dissolving 10 g of gelatin (G2500, Sigma) in 60 mL distilled water, heating to melt, adding 70 mL glycerol (G5516, Sigma) and 0.25 g of phenol (33517, Sigma), and mixing well. The glycerine jelly was stored at room temperature. The resulting images were observed using a light microscope and analyzed by cellSens imaging software.

### ALT, AST, and ALP assay

Blood was collected from the mouse by cardiac puncture and allowed to stand at room temperature for 1 h. Afterward, it was centrifuged at 1500×*g* for 10 min to separate the supernatant. The supernatant obtained after centrifugation is referred to as serum. The serum was then diluted 1:2 with water, The activities of alanine aminotransferase (ALT), aspartate aminotransferase (AST), and alkaline phosphatase (ALP) were measured using an automated biochemistry analyzer (BS-240, Mindray, Shenzhen, China).

### Free fatty acid preparation

The palmitic acid stock solution was prepared using fatty acid-free BSA (A8806, Sigma) at a molar ratio of 3.3:1 (palmitic:BSA) [45]. To prepare the stock solution, 500 mM palmitic acid (P0500, Merck) was dissolved in absolute ethanol at 70 °C. Then, 10 μL of palmitic acid solution was added to 1 mL prewarmed 10% BSA (prepared in cell culture medium). The mixture was incubated at 55 °C until it became clear and was then filtered before use. For the vehicle control, 10 μL ethanol was mixed with 1 mL 10% BSA. Oleic acid was prepared at a molar ratio of 6:1 (oleic acid:BSA) using the product (29557, Cayman, MI, USA). The vehicle stock solution was prepared by dissolving 0.8 mM BSA in 150 mM sodium chloride (S3014, Sigma).

### Conditioned medium collection and activity test

Conditioned medium was collected from AML-12, MIHA, and primary hepatocytes isolated from wild-type or FGF21 knockout C57BL/6J mice for use in this study. In detail, stock solutions of FFAs were diluted with serum-free medium and used to treat hepatocytes for a period of 24 h. After this treatment, the cells were washed twice with PBS and then cultured in the serum-free medium for an additional 12 h. Following the incubation period, the medium was collected from the cells. To remove any cellular debris, the collected medium was filtered through a 0.22 μm filter (16532-K, Sartorius, Göttingen, Germany).

For the activity test, the conditioned medium obtained previously was diluted with serum-free medium to create a 25% working solution. This working solution was then used to treat different breast cancer cell lines, MDA-MB-231, BT-549, MCF-7, E0771, and 4T1. The cell lines were seeded in a 96-well plate and treated with the diluted conditioned medium for a duration of 24 h. After the treatment period, cell viability was analyzed using the MTS assay.

### RNA sequencing and analysis

Liver tissues from HFD-fed C57BL/6J mice were prepared for RNA sequencing. The mouse liver was immersed in RNAiso Plus (9109, Takara, Shiga, Japan) and homogenized using LabServ Fast-24 (Thermo Fisher) to extract mRNA. Chloroform (650498, Sigma) was used for RNA purification, followed by cleaning with 75% cold ethanol. The RNA was left to dry and then dissolved in RNase-free water (10977015, Thermo Fisher). The purity and concentration of RNA were analyzed using absorbance measurements (EW-83056-26, Jenway, IL, USA).

For each group, mRNAs from two mice were mixed to create one sample, and three non-overlapping samples from each group were used for further analysis. The preparation of RNA library and transcriptome sequencing was conducted by Novogene Co., Ltd (Beijing, China). The genes between groups were analyzed using DESeq2 and considered differentially expressed when adjusted *p* ≤ 0.05 and |log2(FoldChange)| ≥ 1. The protein sequences of significantly changed genes were obtained from GenBank [46] and tested for the presence of signal peptides using SignalP 6.0 [47]. Genes with a probability >0.5 were further evaluated in GeneCard [48] and UniPort [49] for their extracellular activities.

### RT-qPCR

Cells were lysed using RNAiso Plus, and total RNA was isolated following the procedures mentioned earlier. For both cells and liver tissues, 1 μg total RNA was used for genomic DNA elimination. The RNA was then reverse-transcribed into cDNA using PrimeScript^TM^ RT Master Mix (RR047A, TaKaRa) as per the manufacturer’s instructions. Real-time PCR reactions were carried out using TB Green Premix Ex Taq^TM^ (RR820A, Takara) and 15 ng of cDNA templates with 0.4 μM primers. The reactions were performed on a Light Cycler 480 system (Roche, Basel, Switzerland). The PCR protocol included 45 cycles of denaturation at 95 °C for 30 s, followed by annealing/extension at 61 °C for 30 s. To analyze the results, the cycle threshold values for each gene were normalized to that of GAPDH or β-Actin. Relative quantification was performed using the 2^−ΔΔCt^ method. The primers used for the PCR amplification can be found in Supplementary Table S1.

### ELISA

Before the test, serum samples were diluted 1:2 with water. Meanwhile, the conditioned medium obtained from AML-12 and primary hepatocytes was used without any dilution. The mouse serum and conditioned medium from AML-12 and primary hepatocytes were analyzed for FGF21 concentration using a mouse FGF21 ELISA kit (32180, IMD), while the conditioned medium from MIHA cells was analyzed using a human FGF21 ELISA kit (31180, IMD). The manufacturer’s protocol was followed for each respective kit during the detection process. The standard curve and data were analyzed by an online data analysis tool [50].

### In vivo FGF21 administration

In the FGF21 peritumoral injection model, 6-week-old female C57BL/6J mice weighing 20.4 ± 1.25 g were used. To establish the model, 1 × 10^6^ E0771 cells were suspended in PBS and injected into the second mammary fat pad of each mouse. One week after injection, tumor-bearing mice were randomly divided into two groups.

For the FGF21 treatment group, a solution of 0.08 mg/kg recombinant mouse FGF21 solution was prepared in 20 μL of water and injected using an insulin syringe (29 G, TERUMO, Tokyo, Japan). The needle was inserted subcutaneously, positioned at a distance of 2 mm from the tumor periphery and the FGF21 was slowly injected. The needle was left in place for an additional 5 s before withdrawal to prevent any leakage from the injection point. In the vehicle group, 20 μL water was administrated to the mice using the same method. The treatment was continued for 24 days.

In the FGF21 sustained-release model, FGF21 was prepared in water at a concentration corresponding to a release rate of 0.08 mg/kg/day. This solution was then packed into an osmotic pump (1004 W, RWD, Guangdong, China) following the manufacturer’s instructions. For the experiment, 6-week-old female C57BL/6J mice weighing 19.85 ± 1.45 g were anesthetized. A small incision was made in the dorsolumbar skin, and a pocket was created using a hemostat to implant the osmotic pump. The incision was then stitched with sutures. After the surgical procedures, the mice were injected with 1 × 10^6^ E0771 cells in the second mammary fat pad. They were then housed in the ICU until recovered from the anesthesia. The osmotic pump continuously infused the FGF21 solution for a duration of 4 weeks.

### Flow cytometry analysis of apoptosis

Breast cancer cell lines MDA-MB-231, MCF-7 and E0771 were seeded in 12.5 cm^2^ flasks at a density of 1 × 10^5^ cells per flask. These cells were then treated with 50 ng/mL of FGF21 and DOX at different concentrations: 1.25 μM for MDA-MB-231 and E0771, 5 μM for MCF-7. The treatment duration was 24 h. After the treatment period, both the floating and attached cells were harvested and resuspended in the testing buffer. These cells were then stained with Annexin V Apoptosis Detection Kit (559763, 556547, BD, NJ, USA) according to the manufacturer’s protocol. The stained cells were analyzed with Agilent NovoCyte Advanteon BVYG flow cytometer (Agilent). To ensure accurate analysis, tubes containing only fluorescent dyes were used for the compensation in the control groups. Additionally, tubes containing DOX were used for extra compensation in the treatment groups. The obtained data were analyzed using FlowJo^TM^ software (version 10.7.1, BD) [51].

### TUNEL assay

Breast cancer cells (1.5 × 10^4^) were seeded in 24-well plates and treated with 50 ng/mL of FGF21 and DOX at different concentrations: 0.5 μM for MDA-MB-231 and 2 μM for MCF-7. The treatment duration was 40 h. After the treatment period, the cells were fixed with 4% PFA. Endogenous peroxidase was blocked using a 3% hydrogen peroxide solution (1.07209, Sigma) and the cell membrane was permeabilized with 0.1% Triton X-100. The TUNEL reaction mixture was then prepared for labeling according to the manufacturer’s protocol (11684817910, Roche). The signal was conversed using the DAB substrate and analyzed under the light microscope. The apoptosis rate was quantified based on the proportion of positive cells.

### Caspase activity assay

DOX induces both intrinsic and extrinsic apoptosis. To determine the type of apoptotic pathways that FGF21 rescued, a caspase multiplex activity assay was performed according to the instruction manual (ab219915, Abcam). Briefly, 3000 MDA-MB-231 and E0771 cells, or 4000 MCF-7 cells, were seeded in a 96-well plate overnight and then treated with DOX (1.25 μM for MDA-MB-231 and E0771, 5 μM for MCF-7) and 50 ng/mL of FGF21 for 20 h. Next, 100 μL test buffer containing caspase 3, 9, and 8 substrates were added to each well and the plate was incubated for another 1 h. The fluorescence intensity was detected at specific wavelengths (0430, BMG LABTECH). To ensure accuracy, wells containing equal volumes of treatment solution and the test buffer were set as blank controls for the corresponding groups.

### Mitochondrial membrane potential assay

Breast cancer cell lines MDA-MB-231, MCF-7, and E0771 were plated on a 24-well plate (82426, Ibidi, Bavaria, Germany) at a density of 1.5 × 10^4^ cells per well and cultured overnight. Cells were then treated with 50 ng/mL of FGF21 and DOX (1.25 μM for MDA-MB-231 and E0771, 5 μM for MCF-7). The treatment duration was 20 h. Following the treatment, the cells were incubated with JC-10 staining solution (CA1310, Solarbio, Beijing, China) and Hoechst 33342 (H3570, Invitrogen) for 30 min. Live cells were washed with assay buffer and imaged using the confocal microscope. Laser wavelengths of 488, 561, and 639 nm were applied to observe the fluorescence emitted by the stained cells. The obtained images were analyzed using ZEN software for further analysis and interpretation.

### Cytochrome c release assay

To detect the expression of mitochondrial cytochrome c, a digitonin-based permeabilization method was employed in this study [52, 53]. First, 3000 MDA-MB-231, MCF-7 and E0771 cells were seeded on a slide and cultured in a 24-well plate overnight. The cells were treated with 50 ng/mL FGF21 and DOX (1.25 μM for MDA-MB-231 and E0771, 5 μM for MCF-7). The treatment duration was 20 h. After the treatment, the cells were stained with Mitotracker^®^ red CMXRos (M9940, Solarbio) for 45 min. Following staining, the cells were washed with PBS and fixed with 4% paraformaldehyde for 10 min at 37 °C. To permeabilize the cells, a purified digitonin (0.004%, D5628, Sigma) was used for 2 min at room temperature. The digitonin was prepared according to the manufacturer’s protocol.

Next, the cells were incubated with blocking buffer (3% BSA, 0.2% sodium azide, and 0.1% tween-20 in PBS) for 30 min at room temperature. Subsequently, the cells were incubated with primary antibody (ab110325, Abcam, diluted 1:100) overnight at 4 °C. After the primary antibody incubation, the cells were incubated with Alexa Fluor-conjugated secondary antibodies (ab150105, Abcam) for 1 h at room temperature. Hoechst 33342 was used for counterstaining for 10 min. Finally, the slides were mounted using a mounting medium (S3023, Dako) and analyzed under a confocal microscope (LSM980, ZEISS).

### Quantification and statistical analysis

The number of replicates for each experiment and specific details of statistical analyses conducted were described in the figure legends or main text. Statistical analyses were performed with SPSS^®^ Statistics (version 25, IBM) [54]. Significant differences and notable non-significant differences were indicated in the figures. For the analysis of clinical data, the chi-squared test was used. Survival analysis was conducted using the Kaplan–Meier tool, and differences were analyzed using the log-rank test on Prism (version 9.5.1, GraphPad).

### Supplementary information

SUPPLEMENTAL MATERIAL

original data files

reproducibility checklist

## Data Availability

RNA sequencing data from this study have been deposited at the NCBI, with the accession number lPRJNA943464. All original data reported in this paper is available from the lead contact upon request. Any additional information required to reanalyze the data reported in this paper is available from the lead contact upon request.
