# Supplementary material for: Non-alcoholic fatty liver disease promotes breast cancer progression through upregulated hepatic fibroblast growth factor 21
Source: Cell Death Dis. 2024 Jan 18;15(1):67. doi: 10.1038/s41419-023-06386-8 (PMC10796330; doi:10.1038/s41419-023-06386-8)
Supplement: Supplementary file 1 — SUPPLEMENTAL MATERIAL [file 41419_2023_6386_MOESM1_ESM.docx]

**Non-alcoholic fatty liver disease promotes breast cancer progression through upregulated hepatic fibroblast growth factor 21**

**Author**

Yue SUI^1^, Qingqing LIU^1^, Cong XU^1^, Kumar GANESAN^1^, Zhen YE^2^, Yan LI^3^, Jianmin WU^4^, Bing DU^5^, Fei GAO^2^, Cailu SONG^6^, Jianping CHEN^1,7^

**Affiliations**

1. School of Chinese Medicine, The University of Hong Kong, Pokfulam, Hong Kong, China.

2. Chengdu University of Traditional Chinese Medicine, Chengdu, 611137, China.

3. Xiamen University, Xiamen, 361005, China.

4. School of Pharmacy, Southwest Medical University, Luzhou, 646000, China.

5. South China Agricultural University, Guangzhou, 510000, China.

6. Sun Yat-Sen University Cancer Center, Guangzhou, 510000, China.

7. Shenzhen Institute of Research and Innovation, The University of Hong Kong, Shenzhen, 518000, China.

**Supplementary** **Figure S1-S4**

**Supplementary Materials Table S1** **and Methods**

**
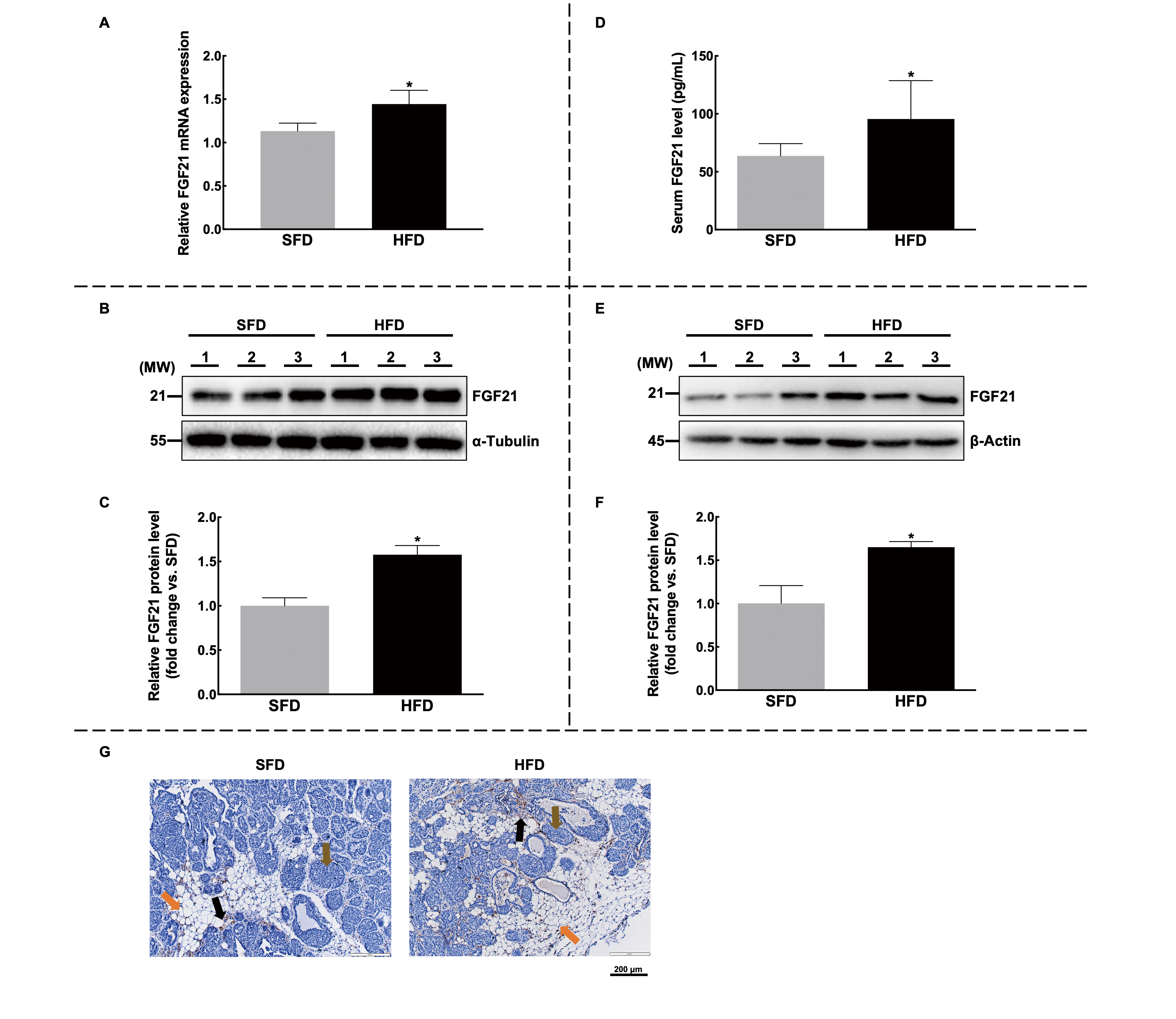
**

**Fig.S1 FGF21 is over-expressed in the liver and tumor tissues of HFD-fed PyVT mice.** (A-C) Hepatic expression levels of FGF21 were detected by RT-qPCR (A) and western bolt (B). Graphs represent the quantification of the blots (C). (D) The serum concentration of FGF21 was determined by ELISA kit. (E, F) Tumoral FGF21 expression levels were detected by western blot (E) and quantified (F). (G) FGF21 in tumor tissues was visualized by immunohistochemistry staining. Images were shown at 4×magnification, scale bars 200 μm. Black arrow: peritumor area; Brown arrow: tumor area; Orange arrow: white adipose tissue. *n* = 6 for each group. Data are expressed as mean ± SD. The difference between groups was assessed by Student’s *t-*test, * *p* < 0.05, ***p* < 0.01. Abbreviation: HFD, high-fat diet group; SFD, standard-food diet group.

**
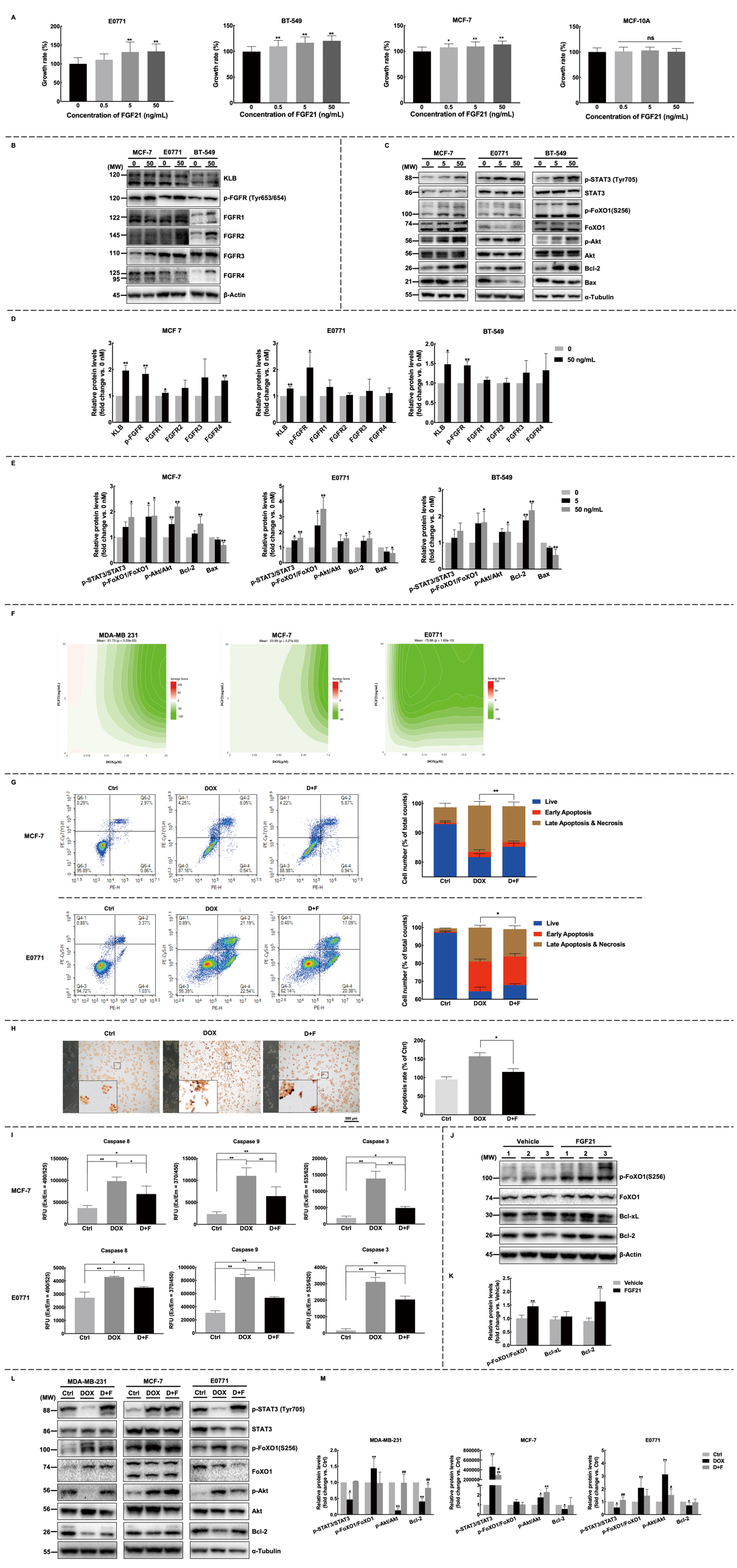
**

**Fig.S2 Recombinant FGF21 promotes the anti-apoptosis ability of Breast cancer cells via STAT3 and Akt/FoXO1 pathways.** (A-E) Cells were treated with recombinant FGF21 (0, 0.5, 5, 50 ng/mL) for 24h before cell viability assay (A) or harvested for western blot analysis (B, C). Graphs represent the quantification of the blots (D, E). MCF-10A is the normal mammary epithelial cell. (F) Antagonistic interactions between DOX and FGF21 were analyzed by the Bliss model. (G) The apoptosis proportion of MCF-7 and E0771 was detected with the PE Annexin V apoptosis detection kit after being treated with 50 ng/mL FGF21 and doxorubicin (1.25 μM for E0771, 5 μM for MCF-7) for 20 hours. The representative images were shown (left) and quantified (right). The difference in late apoptosis between D and D+F groups was marked. (H) TUNEL assay of MCF-7 was performed after cells were treated with 50 ng/mL FGF21 and 5 μM doxorubicin for 20 hours. Representative images were shown as centered TUNEL staining images with live cell images merged on the left and summarized quantification results on the right. (I) The expression levels of caspase family in cell lines were determined by the caspase activity test. (J-M) The activation of apoptosis pathways was also detected in FGF21 peritumoral injection model (J, K) and cell lines (L, M) by western blot and quantified. Data were expressed as mean ± SD. The difference between groups was assessed by One-way ANOVA combined with Turkey’s test for multiple comparison tests, * *p* < 0.05, ** *p* < 0.01 compared to the control group and ^#^ *p* < 0.05, ^##^ *p* < 0.01 compared to the DOX group. Live cell images were at 10 × magnification, TUNEL staining images were shown at 4×magnification with scale bars 500 μm, and insert images were at 20×magnification. Abbreviation: DOX, doxorubicin treatment group; D+F, doxorubicin plus FGF21 treatment group.


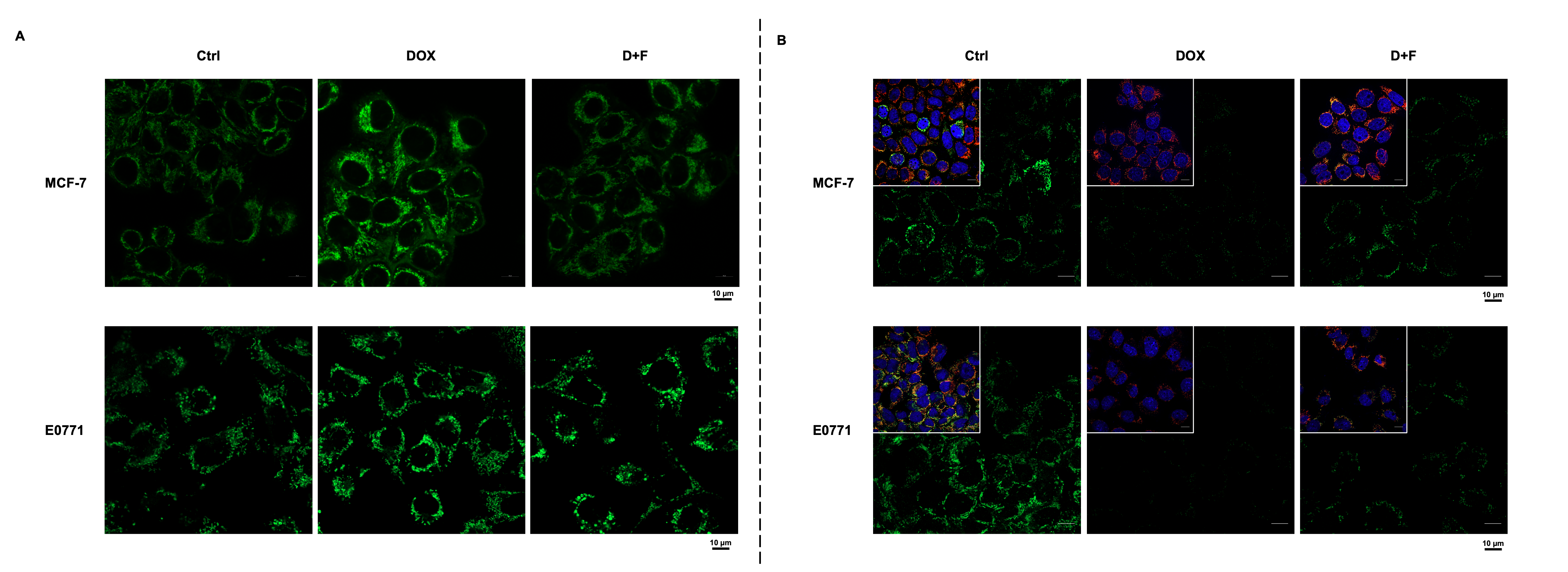


**Fig.S3 FGF21 protects mitochondrial function.** (A) Mitochondrial membrane potential was detected by JC-10. Cells were treated with FGF21 and DOX for 24h before staining with JC-10, and the fluorescence intensity in live cells was then detected by confocal microscopy. (B) The mitochondrial expression levels of cytochrome c were detected via immunofluorescence after being treated with DOX and FGF21 for 24h. Digitonin was used to permeabilize the plasma membrane of cells selectively. Cytochrome c was stained with anti-cytochrome c antibody in green, the nucleus was counterstained with DAPI in blue, and the mitochondrion was stained with mitotracker in red. Images were shown at 40×magnification, and scale bars 10 μm. Abbreviation: DOX, doxorubicin treatment group; D+F, doxorubicin plus FGF21 treatment group.

**
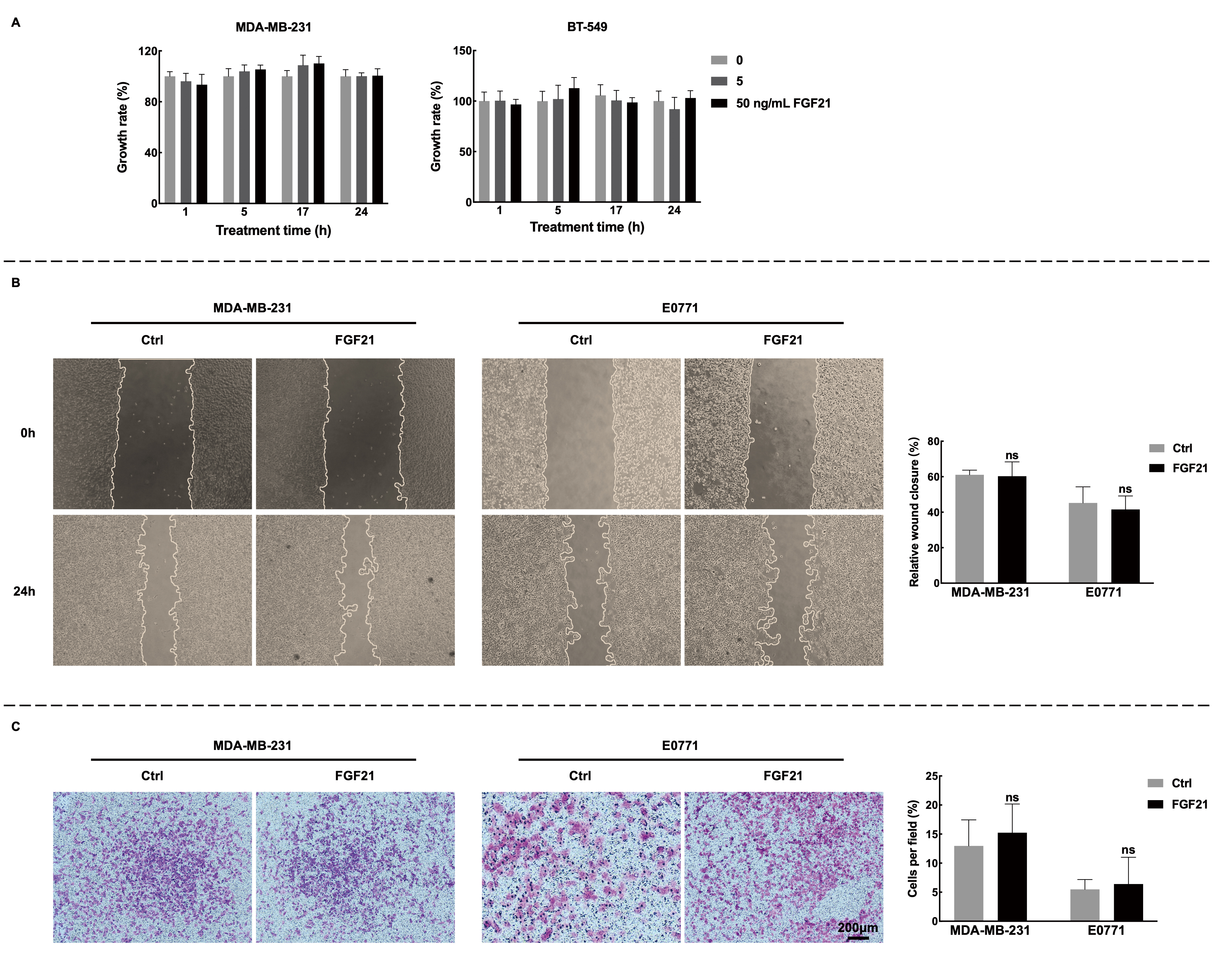
**

**Fig.S4 Recombinant human FGF21 shows no effect on cell proliferation and migration of Bca cell lines. (**A) BrdU assay. Cells were treated with recombinant FGF21 for 40 hours before the BrdU cell proliferation assay. Data were expressed as mean ± SD. The difference between groups was assessed by One-way ANOVA combined with Turkey’s test for multiple comparison tests, and no significant difference was observed between groups. (B) Wound healing assay. Cells were treated with 50 ng/mL FGF21 for 24 hours, the wound area was measured by MRI Wound Healing Tool in Image J. Images were shown at 4×magnification, scale bars 500 μm. (C) Transwell assay. Cells were seeded in the top chambers with 0.1% FBS, and 50 ng/mL FGF21 was added in the lower chamber with 10% FBS. Cell migration was evaluated after 48 hours of incubation by Image J. Images were shown at 4×magnification, scale bars 200 μm. Data were expressed as mean ± SD. The difference between groups was assessed by Student’s *t*-test, ns *p* > 0.05.

**Supplementary Materials Table S1**

**Table S1 RT-qPCR** **primer sequences for genes screened out by RNA sequencing**

| **Primer Name** | **Sequences** |
| --- | --- |
| Mouse FGF21 forward | GCTGCTGGAGGACGGTTACA |
| Mouse FGF21 reverse | CACAGGTCCCCAGGATGTTG |
| Mouse A1bg forward | CGTTTGGTCAGAGGACAGCAAG |
| Mouse A1bg reverse | CATCGAAGCTGCACCGTTGAAC |
| Mouse Gdf15 forward | AGCCGAGAGGACTCGAACTCAG |
| Mouse Gdf15 reverse | GGTTGACGCGGAGTAGCAGCT |
| Mouse Ly6d forward | CATGCTTTAGCCATGATGGAGGC |
| Mouse Ly6d reverse | ATGTCATCAGCTCTTGGTGCCC |
| Mouse Lamb3 forward | TGACCAGACCTATGGACACGTG |
| Mouse Lamb3 reverse | GTCACAGTGACCTCGTTGGCAT |
| Mouse Itga4 forward | GCAAAGAGGTCCCAGGCTACAT |
| Mouse Itga4 reverse | CCTGTAATCACGTCAGAAGTCCC |
| Mouse Serpina12 forward | GGCAGACATCTTTGCCAAGTGG |
| Mouse Serpina12 reverse | CTCCTCAAAGATTTTGCTGATGCC |
| Mouse Vnn1 forward | CTTTCCTCGCGGCTGTTTAC |
| Mouse Vnn1 reverse | CCTCCAGGTATGGGTAGATCGT |
| Mouse Trem2 forward | CTACCAGTGTCAGAGTCTCCGA |
| Mouse Trem2 reverse | CCTCGAAACTCGATGACTCCTC |
| Mouse Apoa4 forward | CAGAAGACGGATGTCACTCAGC |
| Mouse Apoa4 reverse | AGCTGTACGACAAAGGGCACCA |
| Mouse Sema5b forward | GAAGCCGTGGGTCTTTAACTT |
| Mouse Sema5b reverse | CAAGAGCAAGCTGGGAGAAAT |
| Mouse Mmp12 forward | CACACTTCCCAGGAATCAAGCC |
| Mouse Mmp12 reverse | TTTGGTGACACGACGGAACAGG |
| Mouse Col1a1 forward | CCTCAGGGTATTGCTGGACAAC |
| Mouse Col1a1 reverse | CAGAAGGACCTTGTTTGCCAGG |
| Mouse Prss8 forward | GCATCACCTACGATGGCAACCA |
| Mouse Prss8 reverse | TGCTGTAGGAGTCTAGCTGGTG |
| Mouse Esm1 forward | CTGGAGAAACCTGCTACCGTAC |
| Mouse Esm1 reverse | CATTCCATCCCGAAGGTGCCAT |
| Mouse Adgrf1 forward | CCTCCAGAACTCCTCTTTGCCA |
| Mouse Adgrf1 reverse | CCAAAGGAGATGCTGTTACACGG |
| Mouse Ihh forward | CGGCTTCGACTGGGTGTATTAC |
| Mouse Ihh reverse | AGGAAAGCAGCCACCTGTCTTG |
| Mouse Angptl8 forward | GCCTCTATGACAGAGCACTGGA |
| Mouse Angptl8 reverse | AGCTCGAAGGTGTAAAGCGTCC |
| Mouse Adamts7 forward | CCTTGCTACCAAAGCCGCATCA |
| Mouse Adamts7 reverse | CCTTGAAGGTCCTGCTCACAGT |
| Mouse Adam11 forward | CAGTGGTCCTCACCAGCAACTT |
| Mouse Adam11 reverse | GTCTCCAGTAGGTCATCCTGCA |
| Mouse Itih5 forward | AAGTGCTGCCTCTCCACAACAG |
| Mouse Itih5 reverse | GCTTGTTGGACCACAGTAGGCT |
| Mouse Htra4 forward | AGCCACCGTCAAAGACATCG |
| Mouse Htra4 reverse | TGTGGTGCTGACAATCCCTG |
| Mouse Pcsk9 forward | ATGGCACCAGACAGAGGAAGAC |
| Mouse Pcsk9 reverse | CACGCTGTTGAAGTCGGTGATG |
| Mouse Ces2a forward | GCTCTCCAAGTGGCACATTTCC |
| Mouse Ces2a reverse | CAAAGGCAACGTCATCACCATGG |
| Mouse Kcp forward | TGCTTGCACCAGGTCACAGAAC |
| Mouse Kcp reverse | GTGACACATGCAGGAGGAACAG |
| a. produced by Integrated DNA Technologies | |

**Supplementary Methods**

**BrdU assay**

Cell proliferation was tested by colorimetric immunoassay based on the measurement of BrdU incorporation during DNA synthesis (11 647 229 001, Roche). In detail, MDA-MB-231 and BT-549 were seeded in 96 well plates and treated with FGF21 for a certain period of time. BrdU was then added to the wells to label the proliferating cells for 2 hours. DNA is denatured by adding FixDenat, and the anti-BrdU-POD was added to bind the incorporated BrdU. The immune complexes were detected by adding the substrate and quantified by measuring the absorbance at 370 nm with 492 nm as the reference wavelength.

**Wound healing assay**

After cells reached over 90% confluency in 6-well plates, a scratch was made within the monolayer with a sterile 1mL pipette tip. After being washed with PBS, cells were treated by Vehicle or 50 ng/mL FGF21 for 24 hours. The same spots were photographed under microscopy before and after treatment. Cell migration was quantified by the wound area using MRI Wound Healing Tool in Image J.

**Transwell assay**

Cells were seeded in the top chambers with 0.1% FBS, and 50nM FGF21 was added in the lower chamber with 10% FBS. After incubating for 48 hours, cells on the upper surface were erased with cotton swabs, and cells on the lower surface were fixed with 4% paraformaldehyde for 20 min, followed by staining with 1% crystal violet for 30 min. The migration was evaluated with Image J by measuring the average cell density.
