# Supplementary material for: Non-alcoholic fatty liver disease promotes breast cancer progression through upregulated hepatic fibroblast growth factor 21
Source: Cell Death Dis. 2024 Jan 18;15(1):67. doi: 10.1038/s41419-023-06386-8 (PMC10796330; doi:10.1038/s41419-023-06386-8)
Supplement: Supplementary file 2 — original data files [file 41419_2023_6386_MOESM2_ESM.pdf]

PyVT

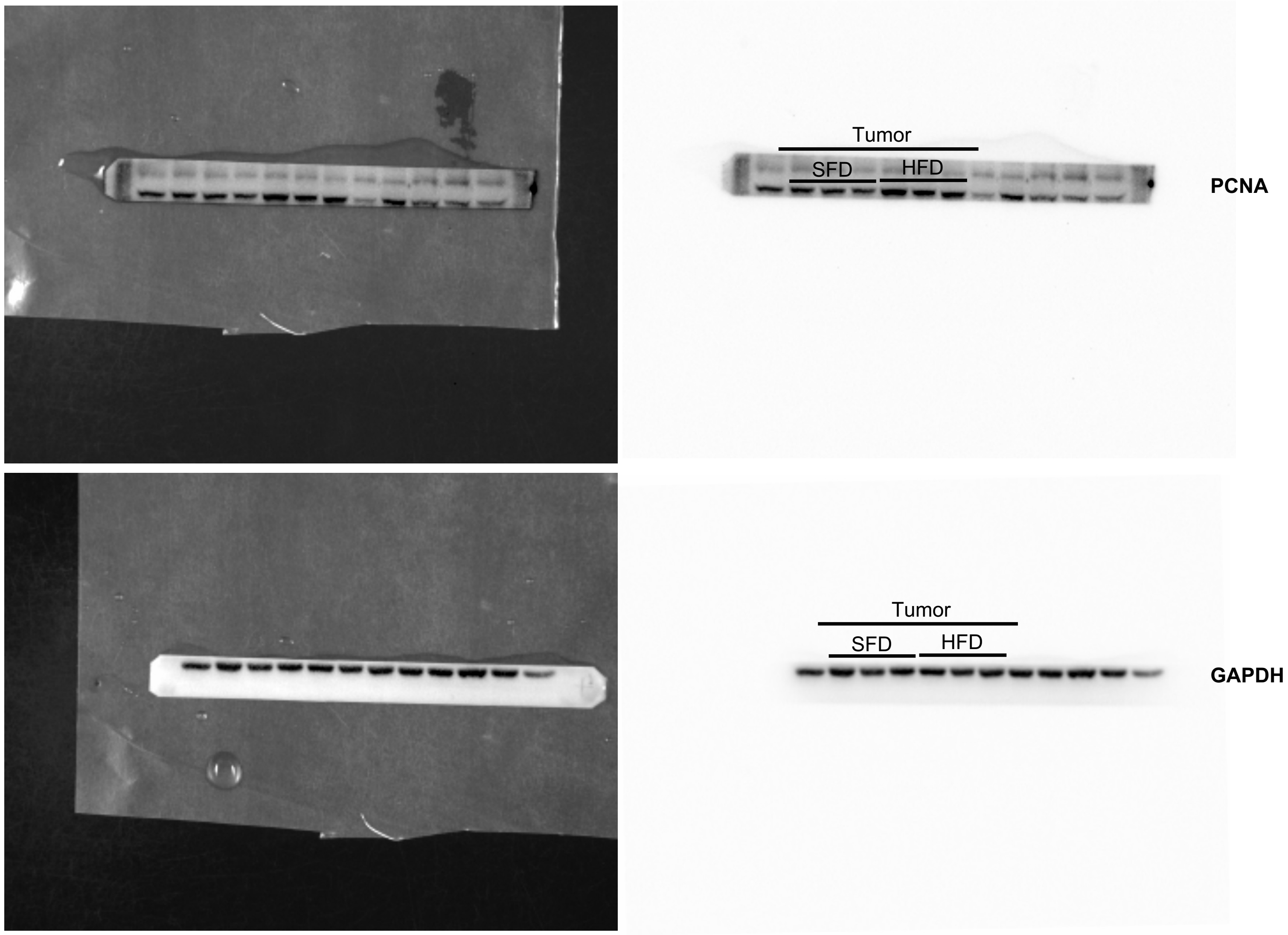

C57BL/6J

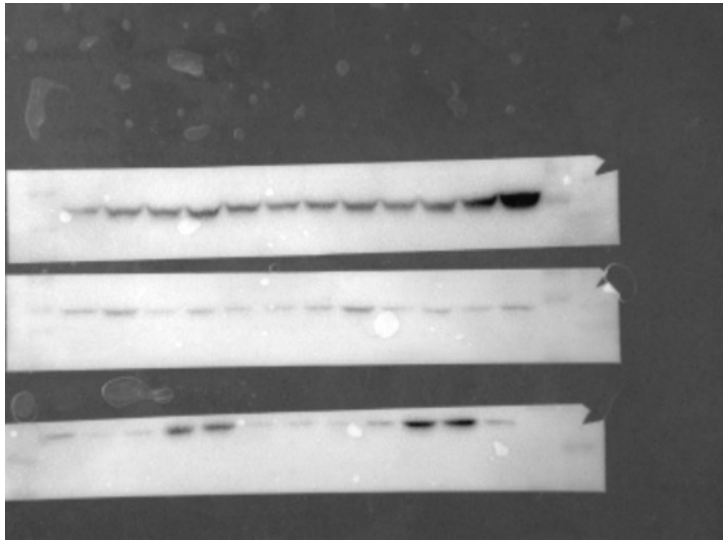

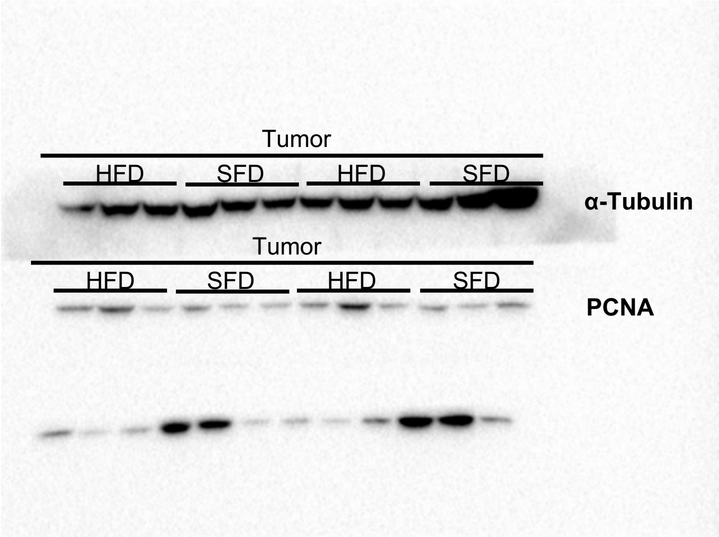

PyVT

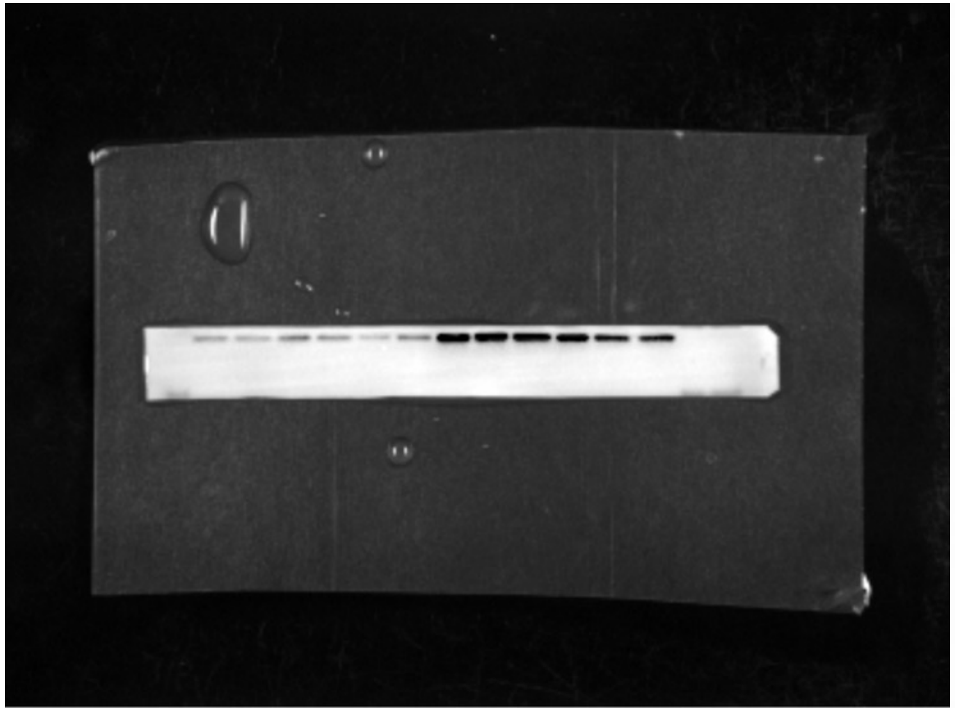

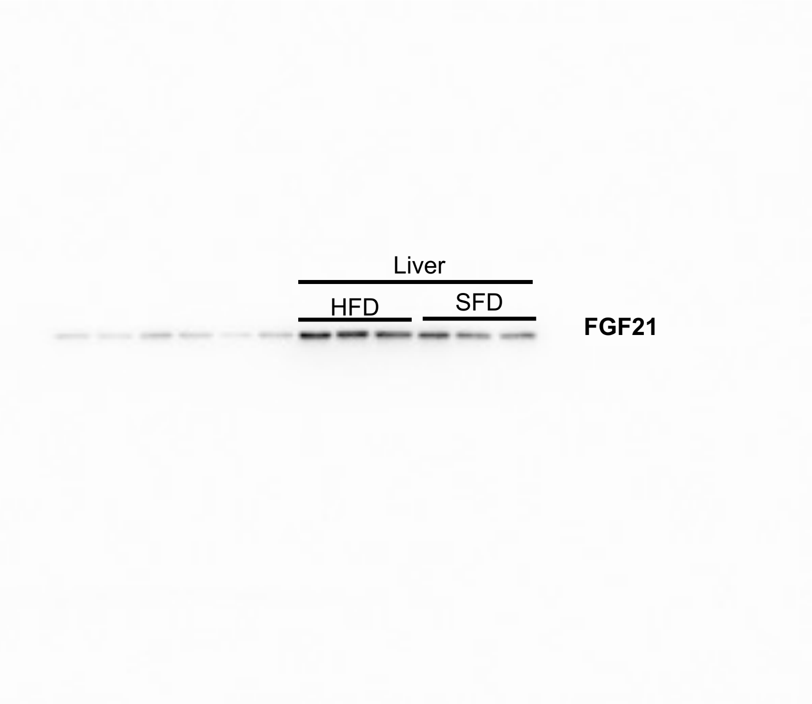

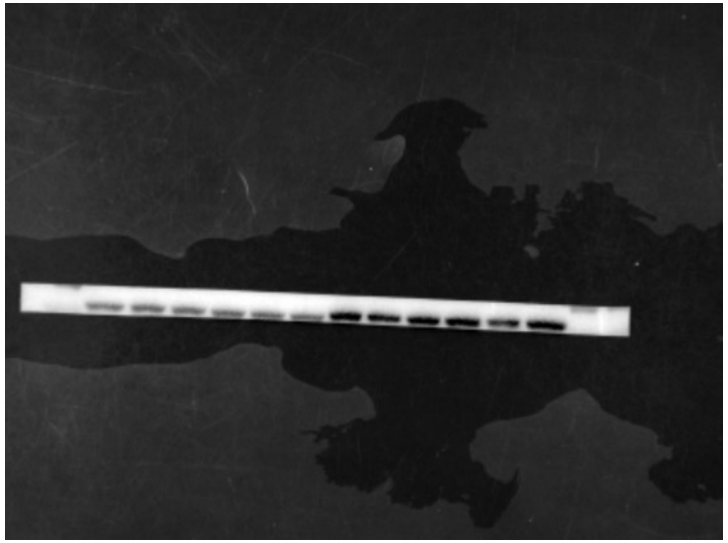

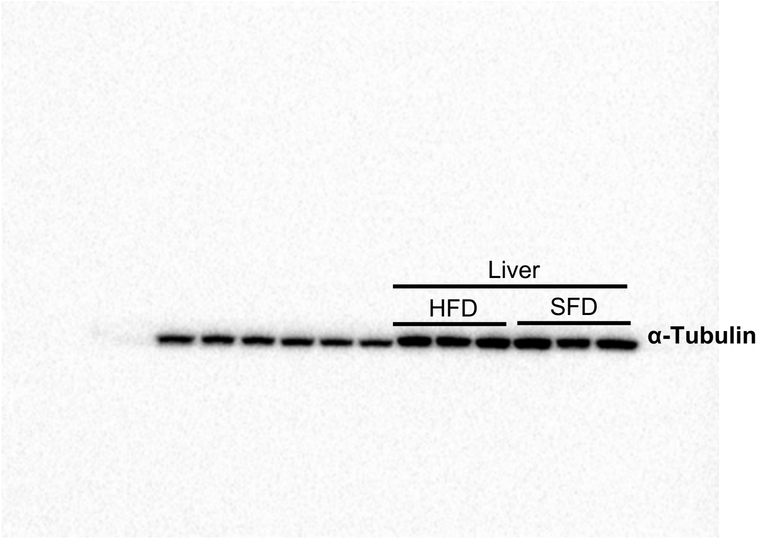

PyVT

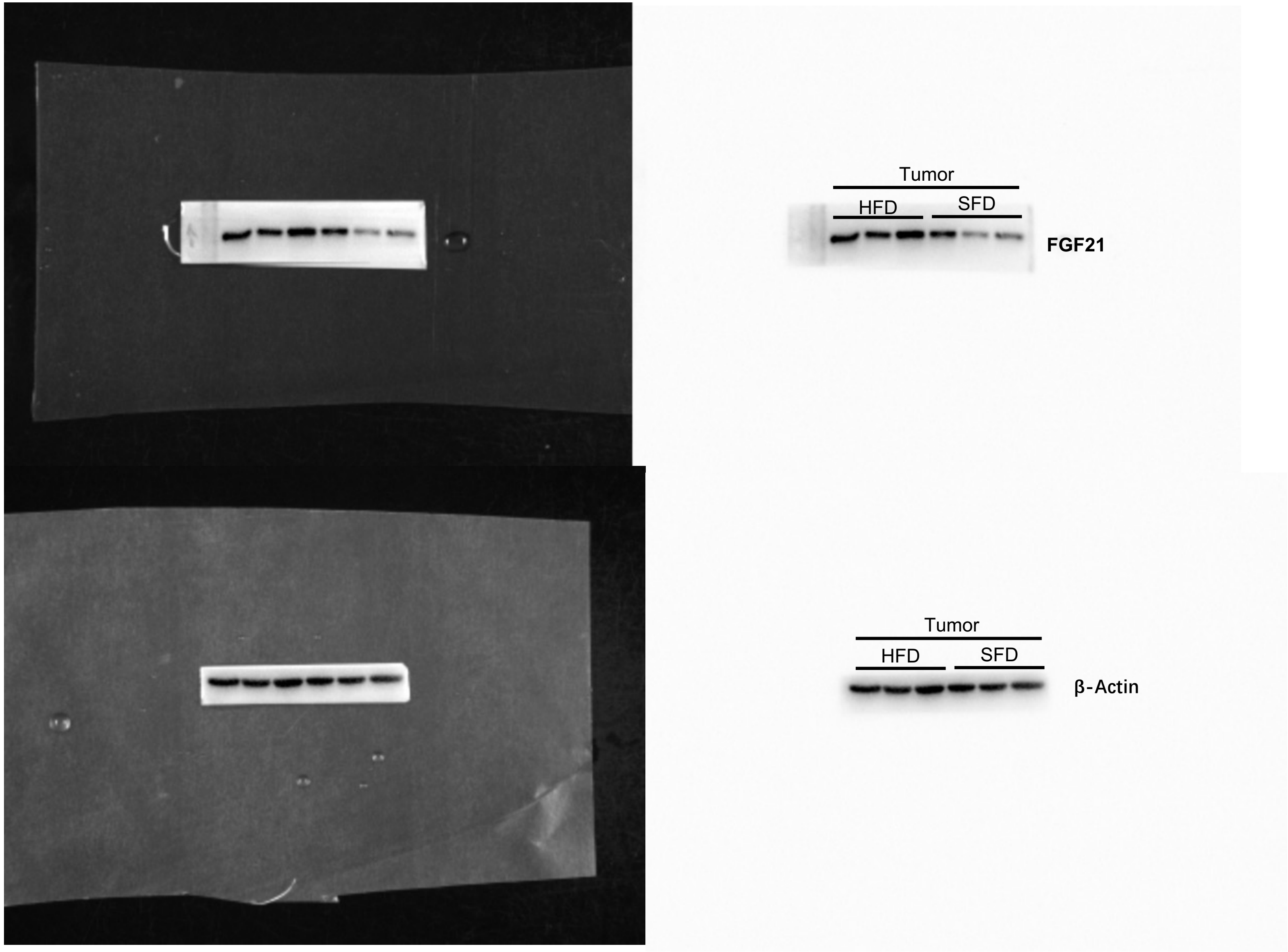

C57BL/6J

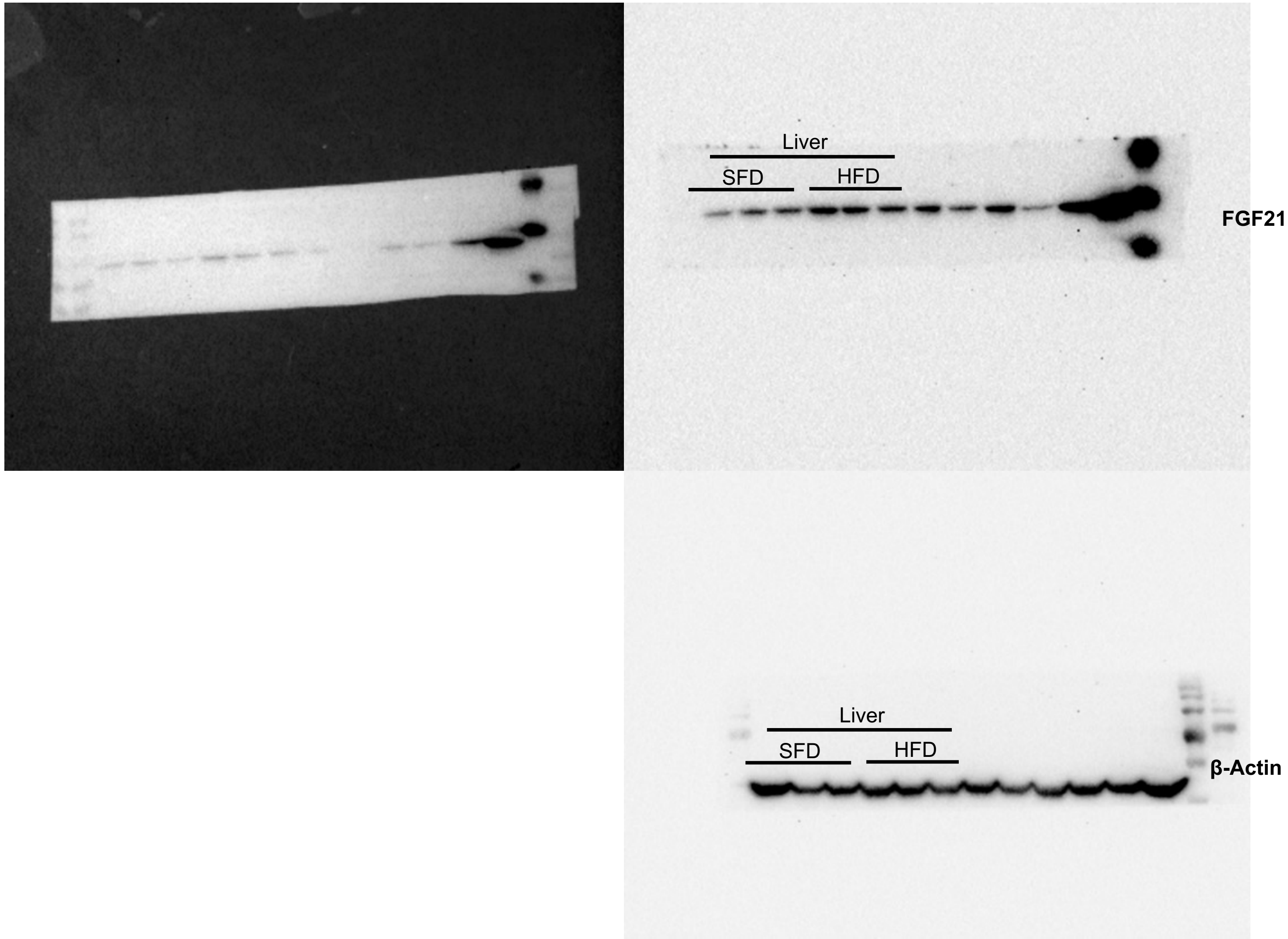

C57BL/6J

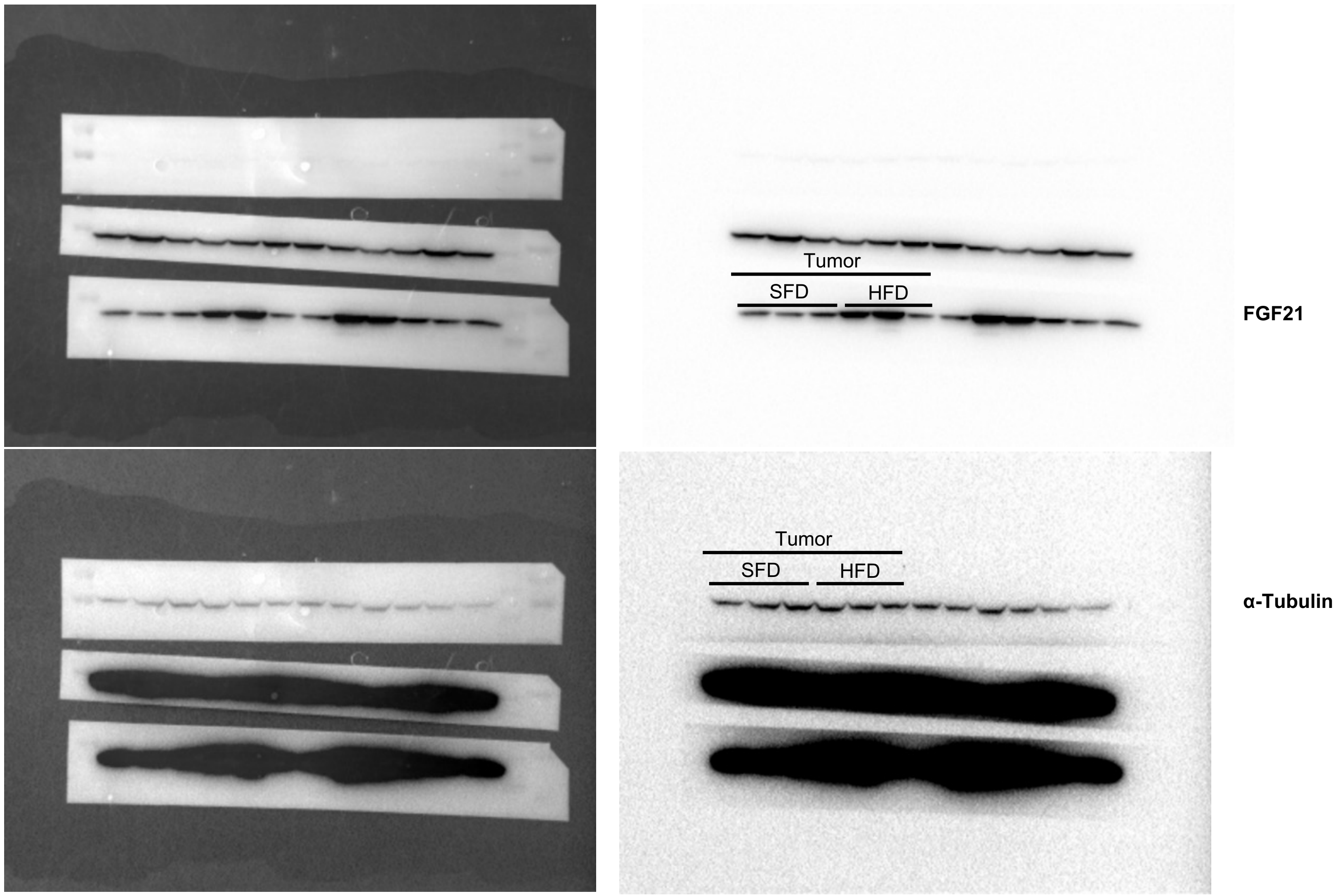

FGFR FGF21

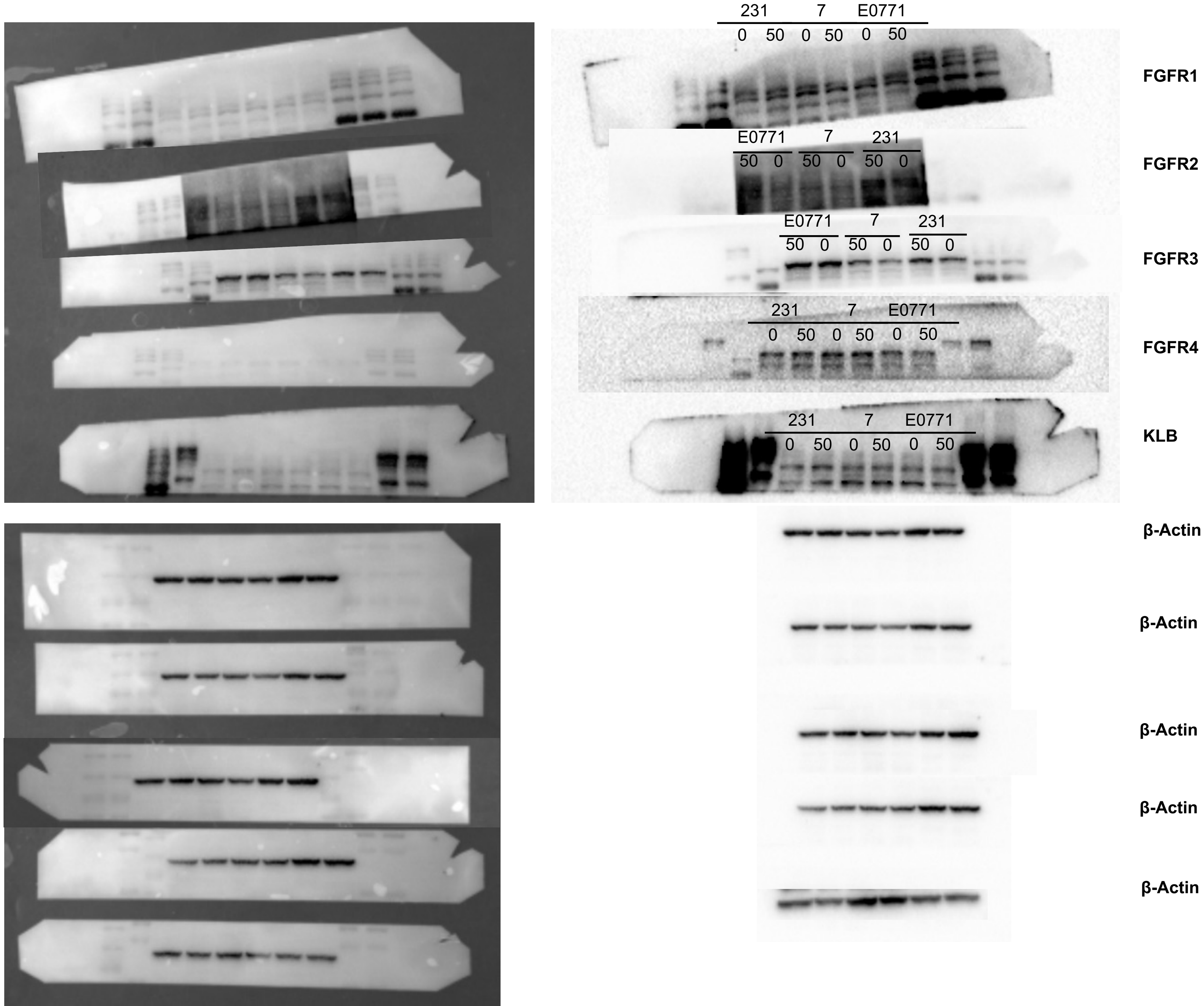

231 7 E0771
0 50 0 50 0 50
FGFR1
E0771 7 231
50 0 50 0 50 0
FGFR2
E0771 7 231
50 0 50 0 50 0
FGFR3
231 7 E0771
0 50 0 50 0 50
FGFR4
231 7 E0771
0 50 0 50 0 50
KLB
β-Actin
β-Actin
β-Actin
β-Actin
β-Actin

FGFR FGF21

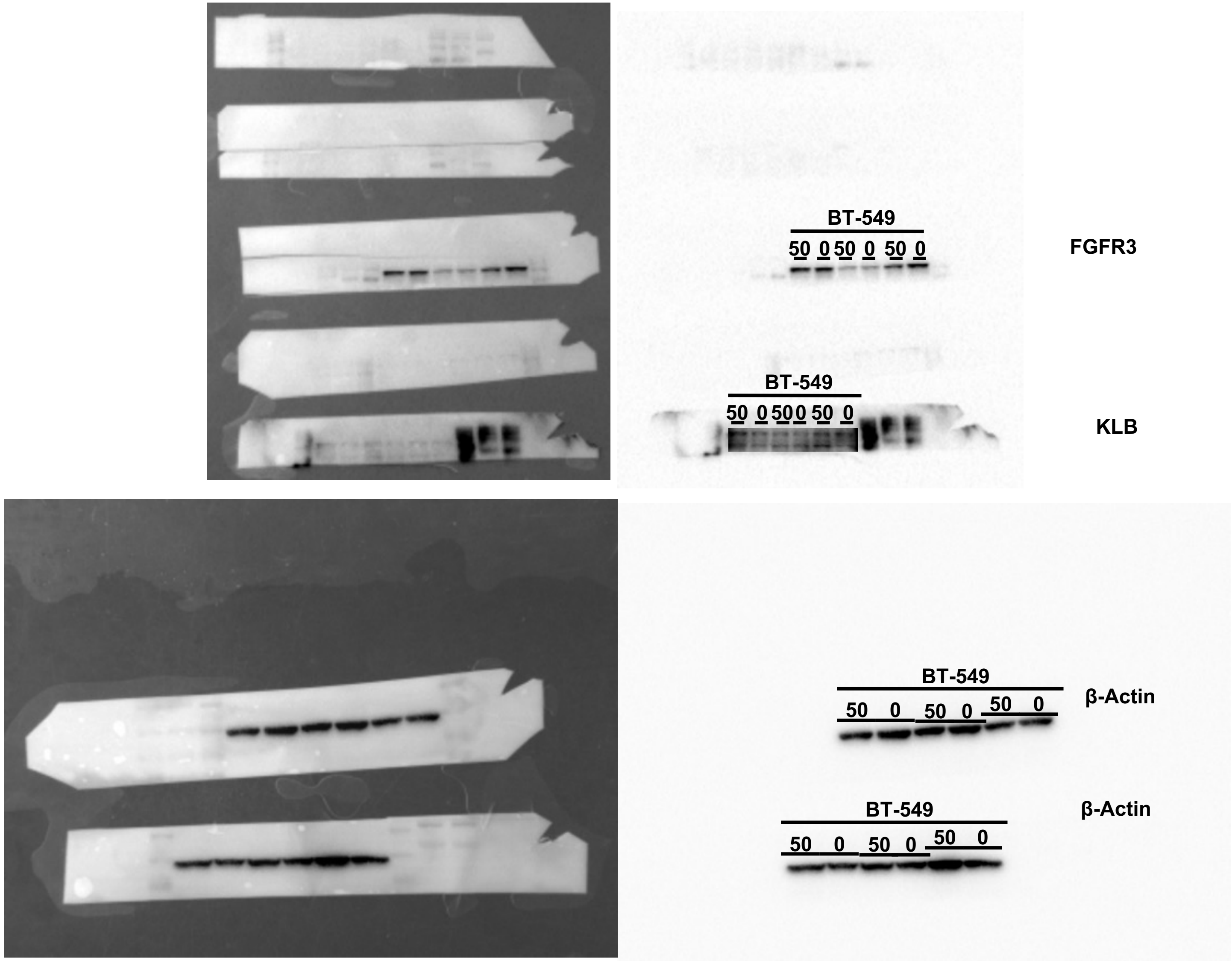
BT-549
50 0 50 0 50 0
FGFR3
BT-549
50 0 50 0 50 0
KLB
BT-549
50 0 50 0 50 0
β-Actin
BT-549
50 0 50 0 50 0
β-Actin

FGFR FGF21

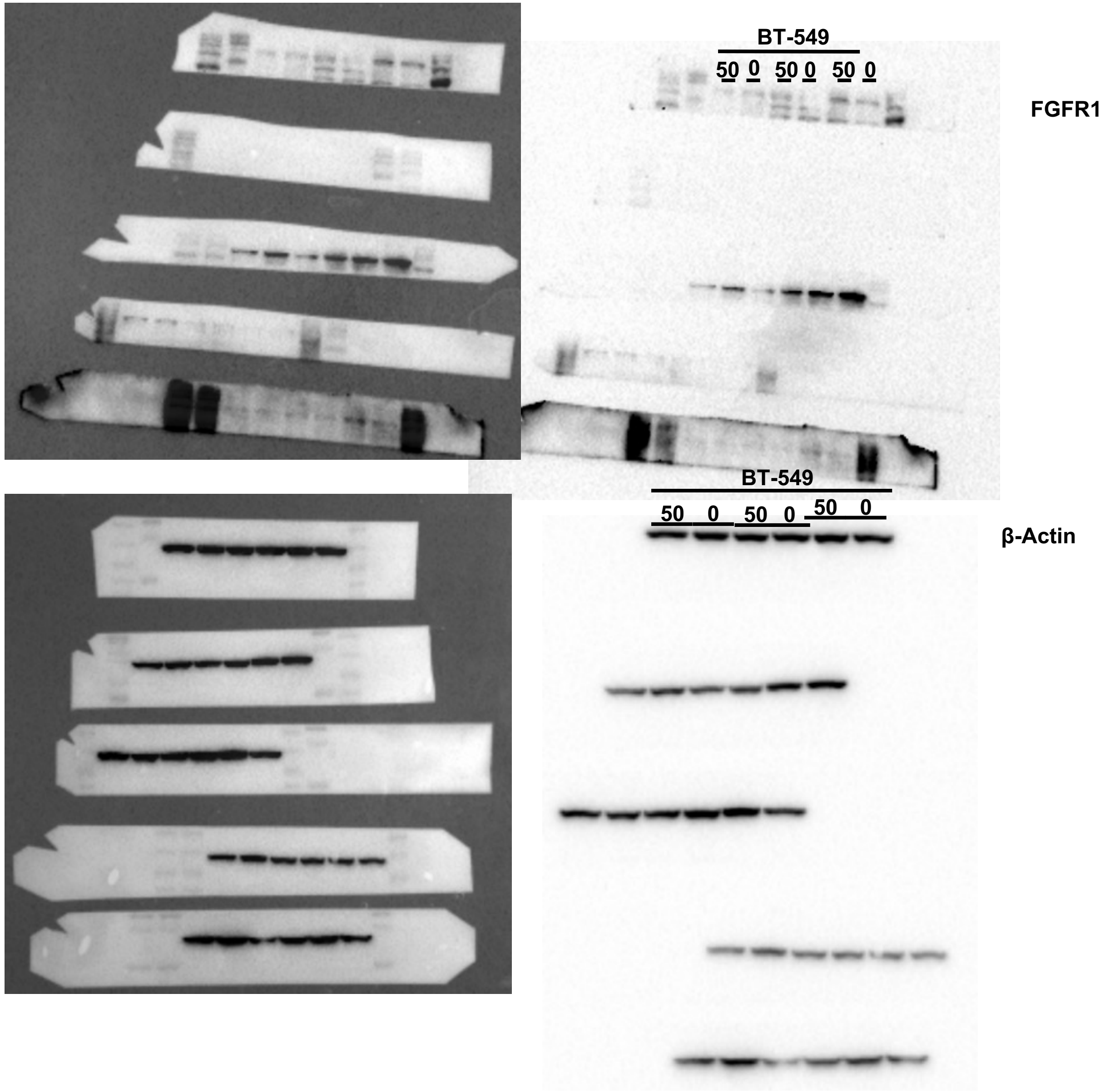

FGFR FGF21

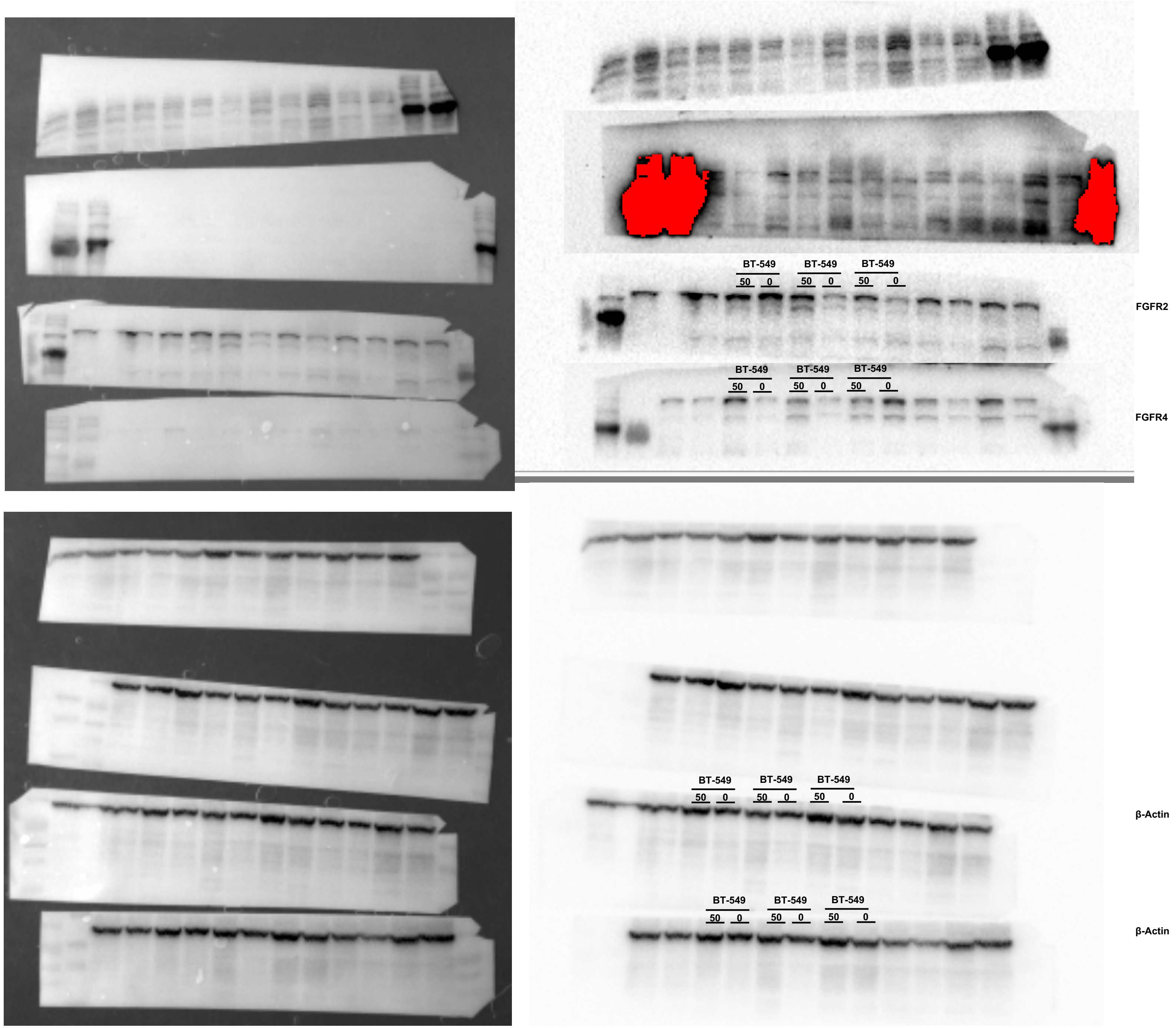

p-FGFR FGF21

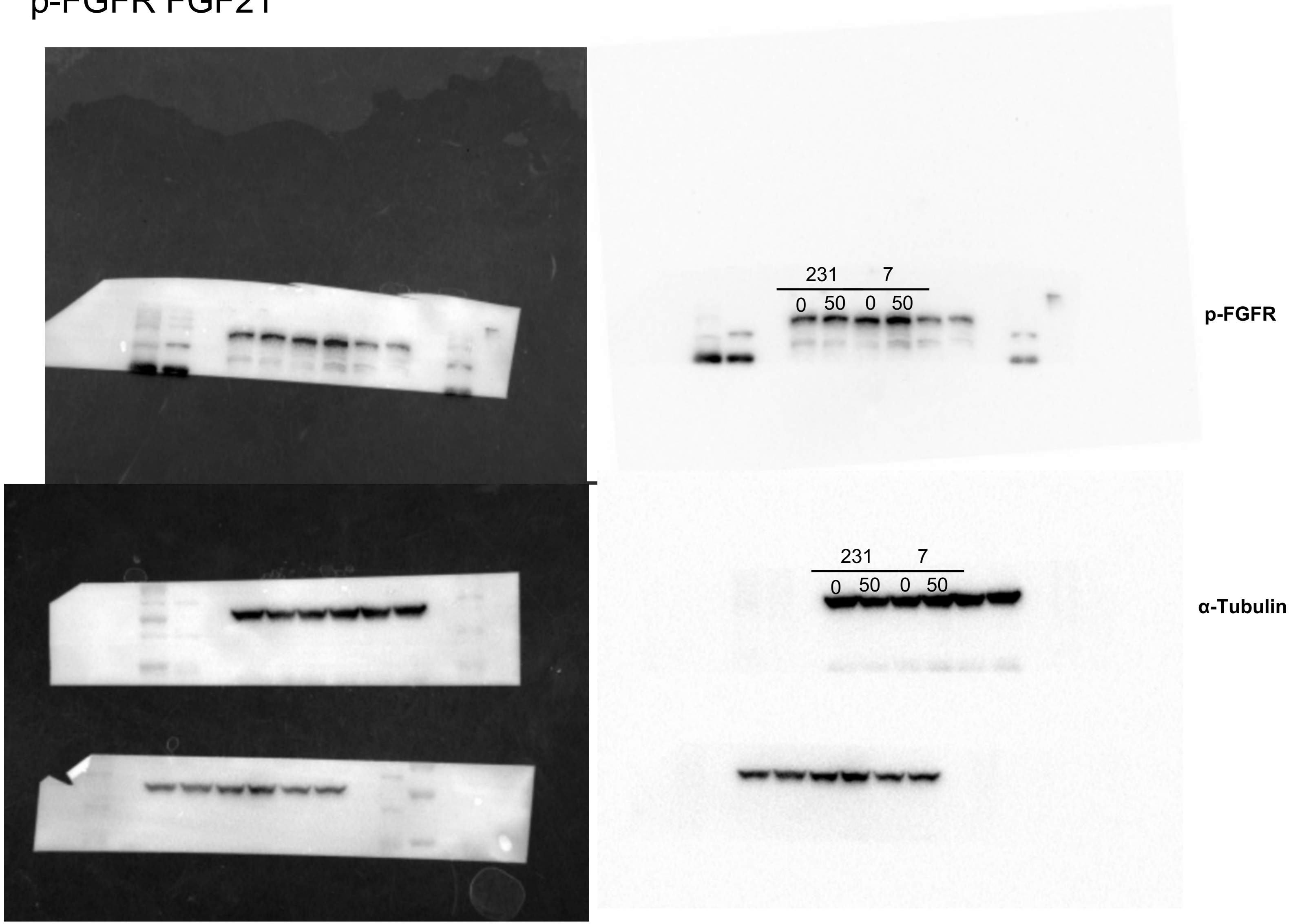

p-FGFR FGF21

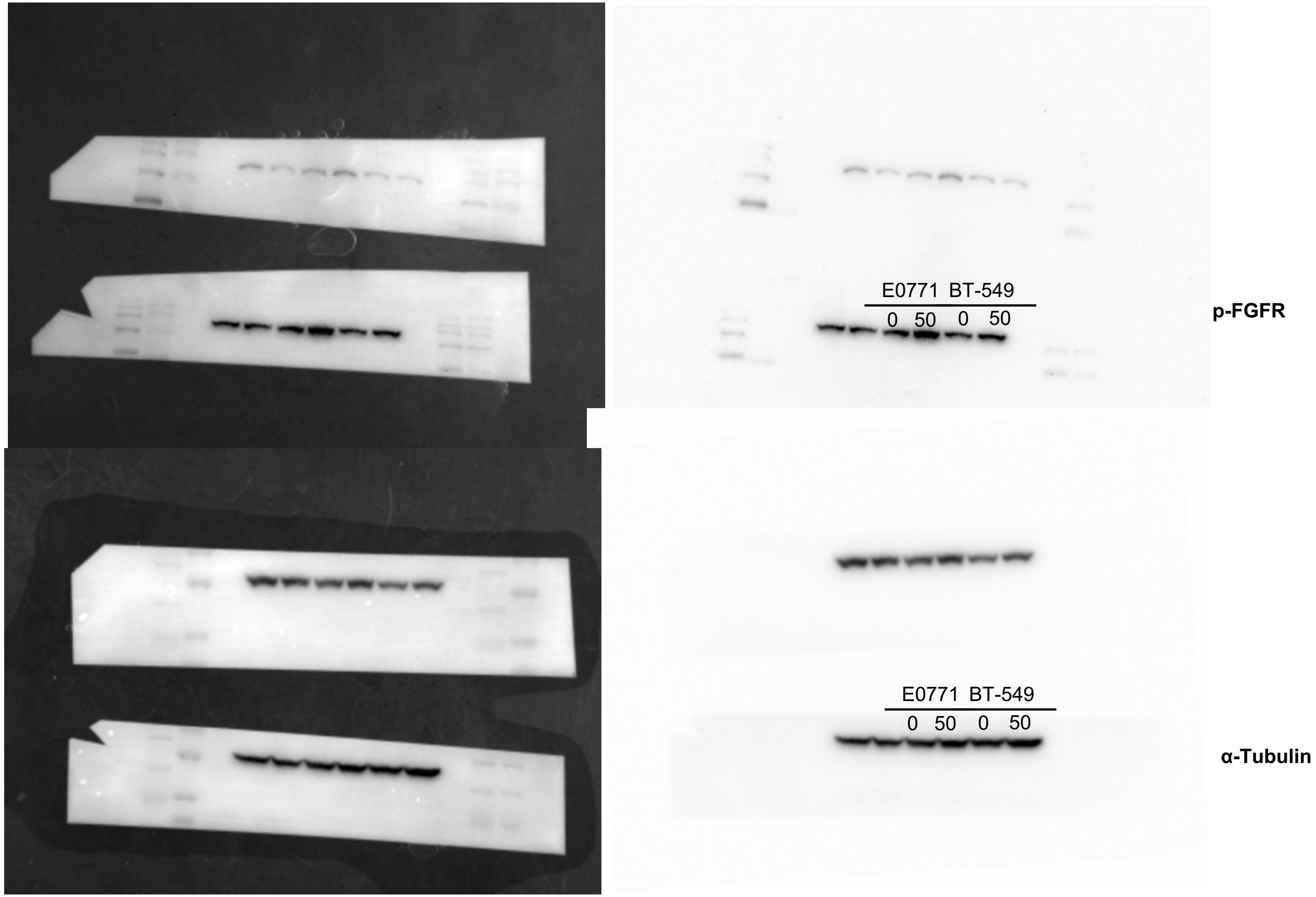

p-FGFR FGF21

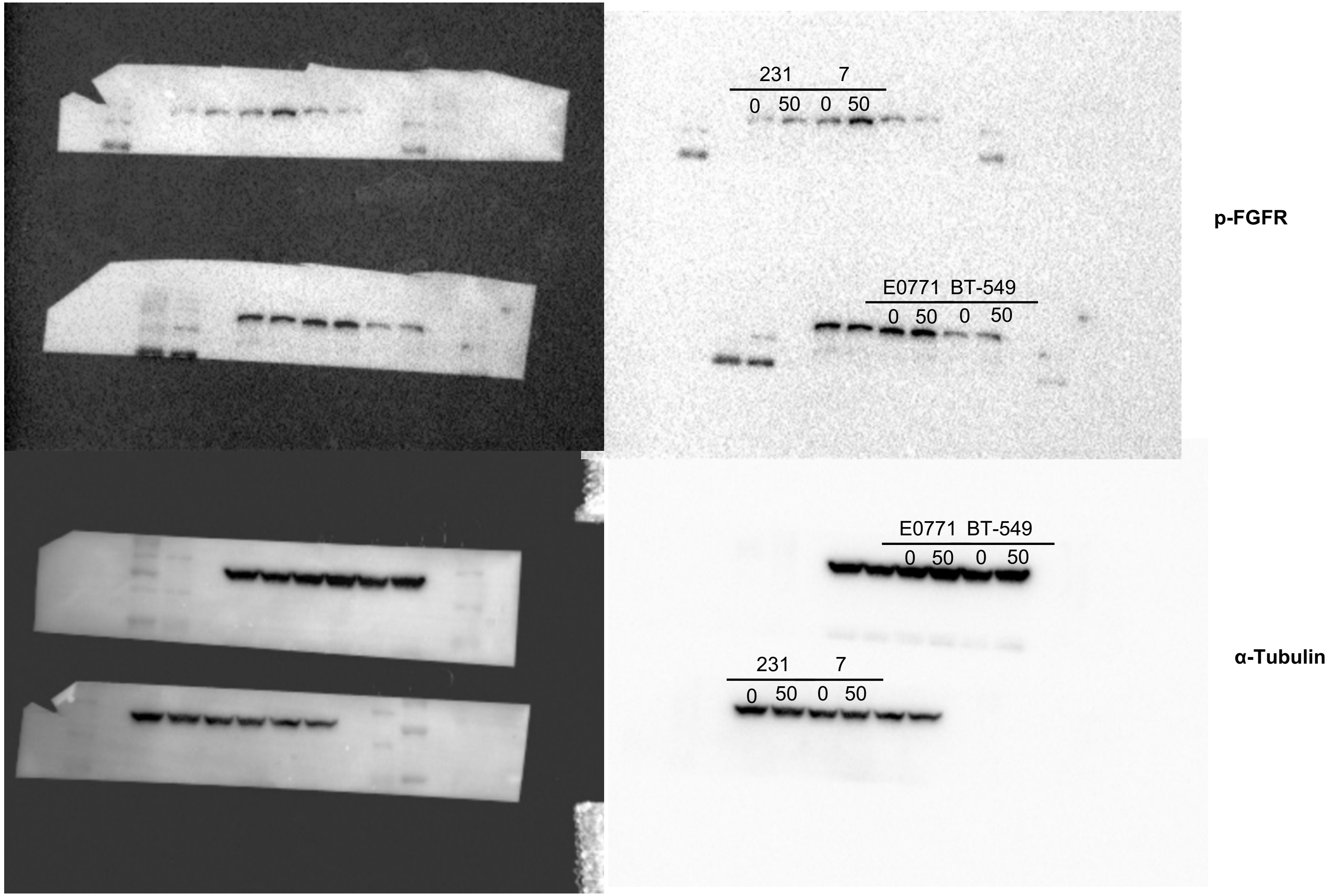
231 7
0 50 0 50
E0771 BT-549
0 50 0 50
p-FGFR
E0771 BT-549
0 50 0 50
231 7
0 50 0 50
α-Tubulin

STAT3 FGF21

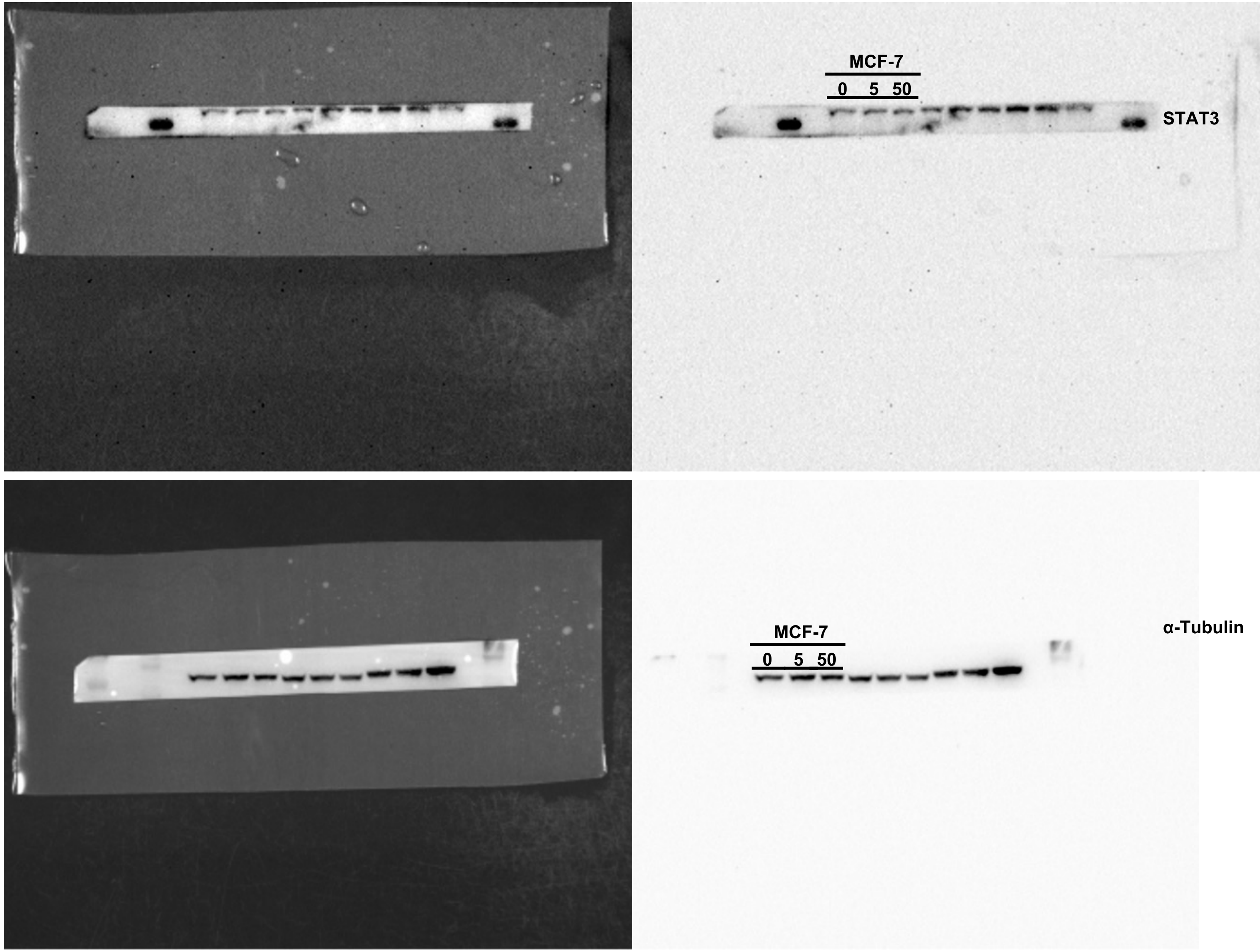

STAT3 FGF21

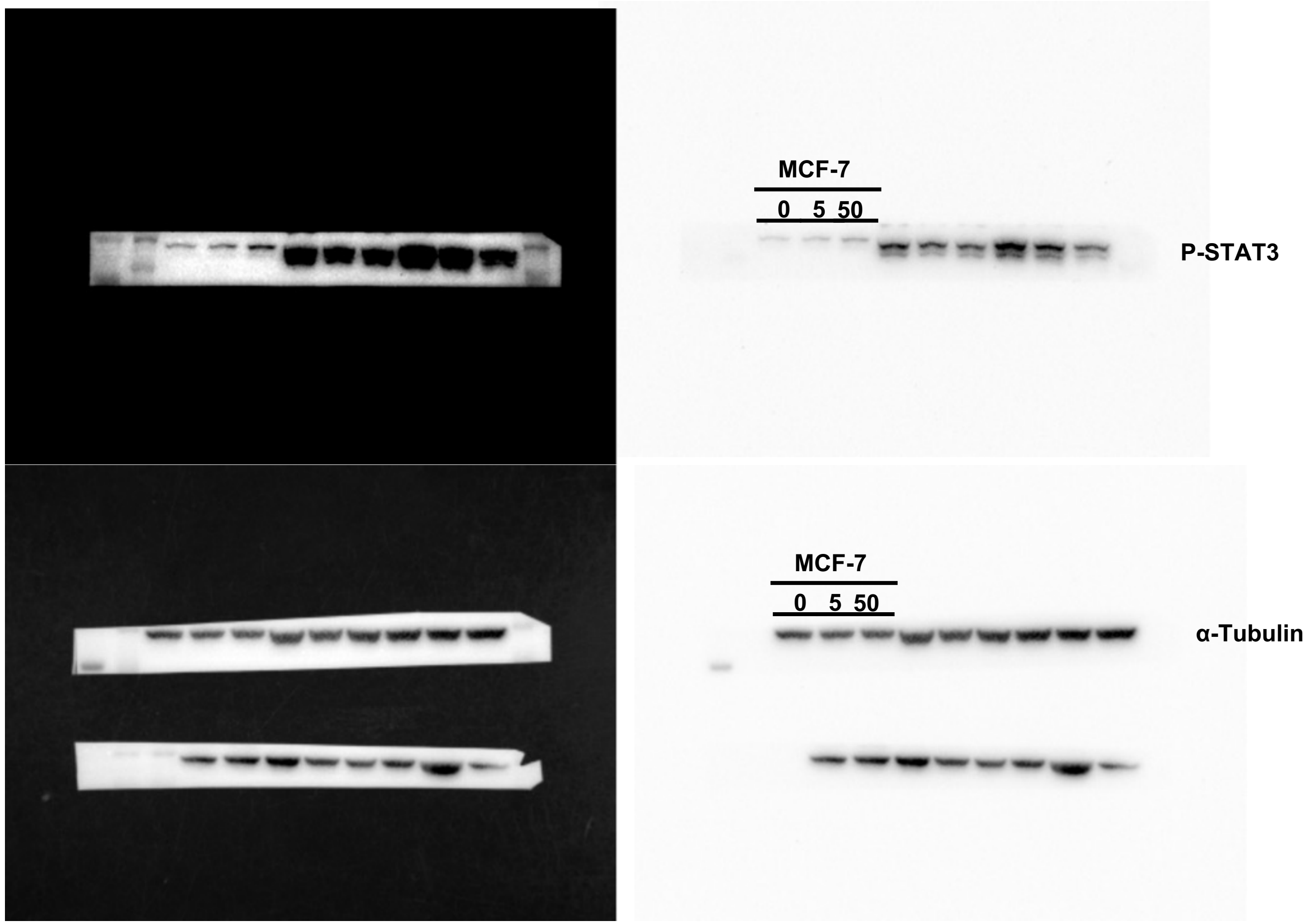

STAT3 FGF21

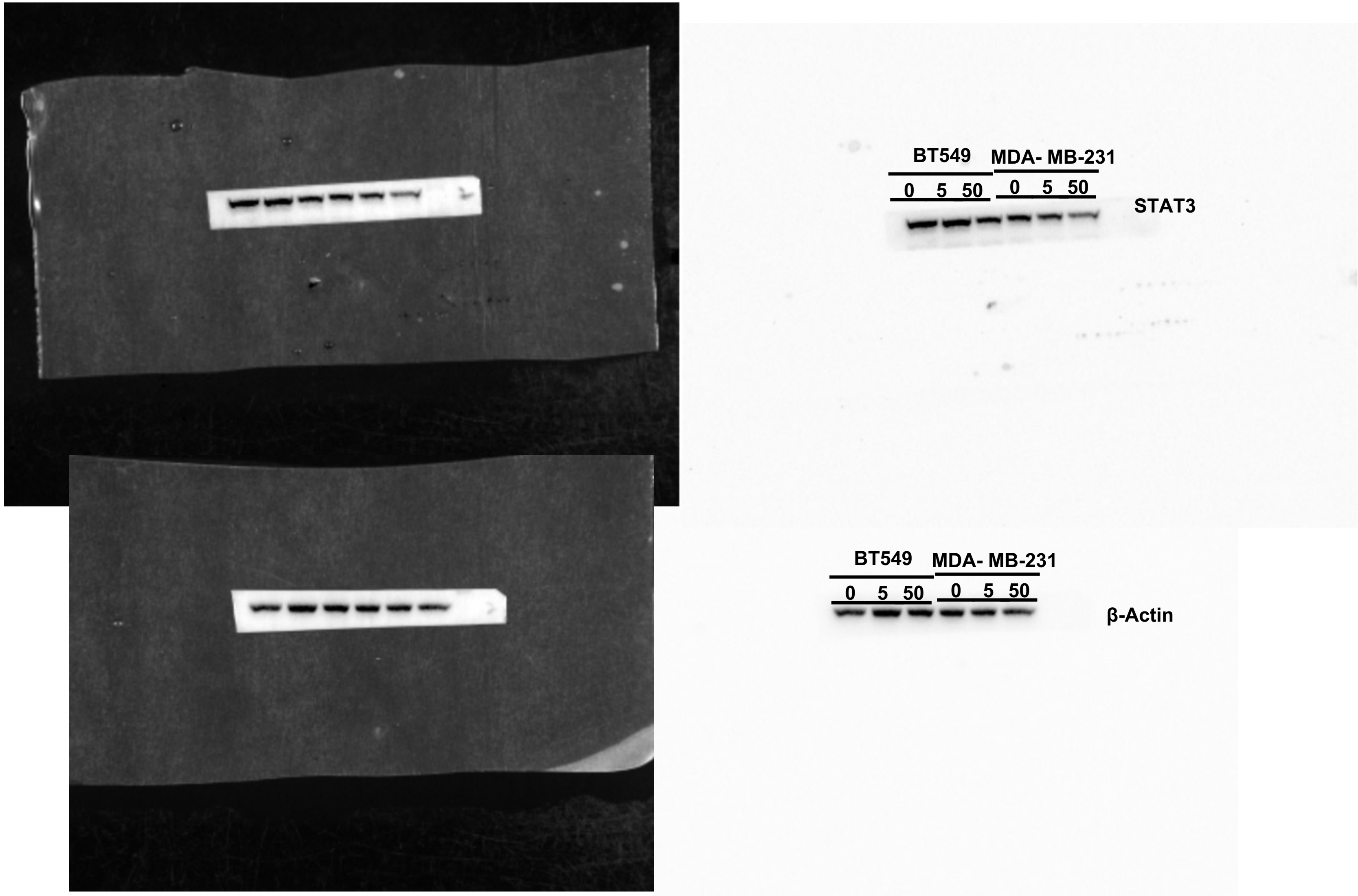
BT549
MDA- MB-231
0 5 50
0 5 50
STAT3
BT549
MDA- MB-231
0 5 50
0 5 50
β-Actin

STAT3 FGF21

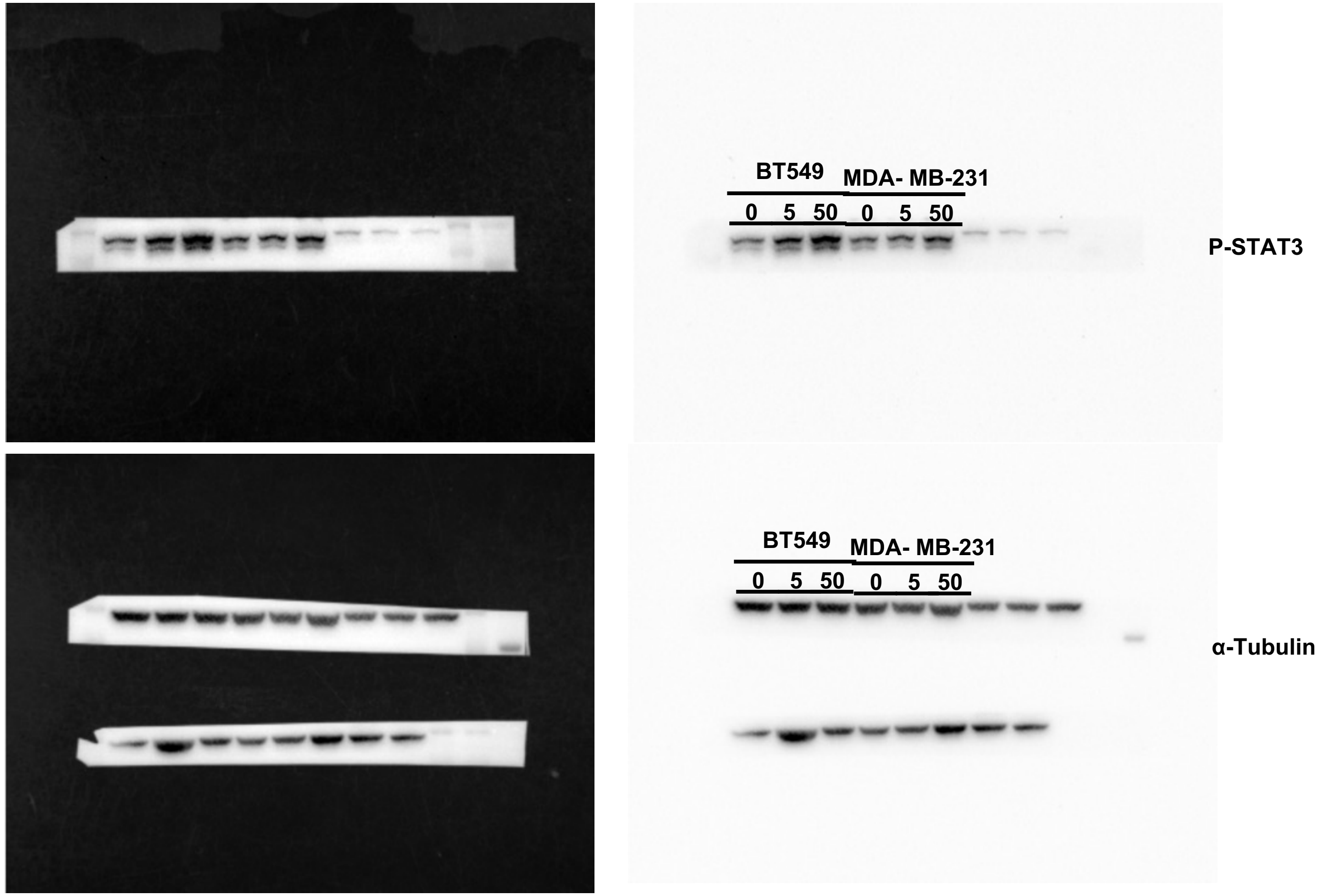
BT549
MDA- MB-231
0 5 50 0 5 50
P-STAT3
BT549
MDA- MB-231
0 5 50 0 5 50
α-Tubulin

STAT3 FGF21

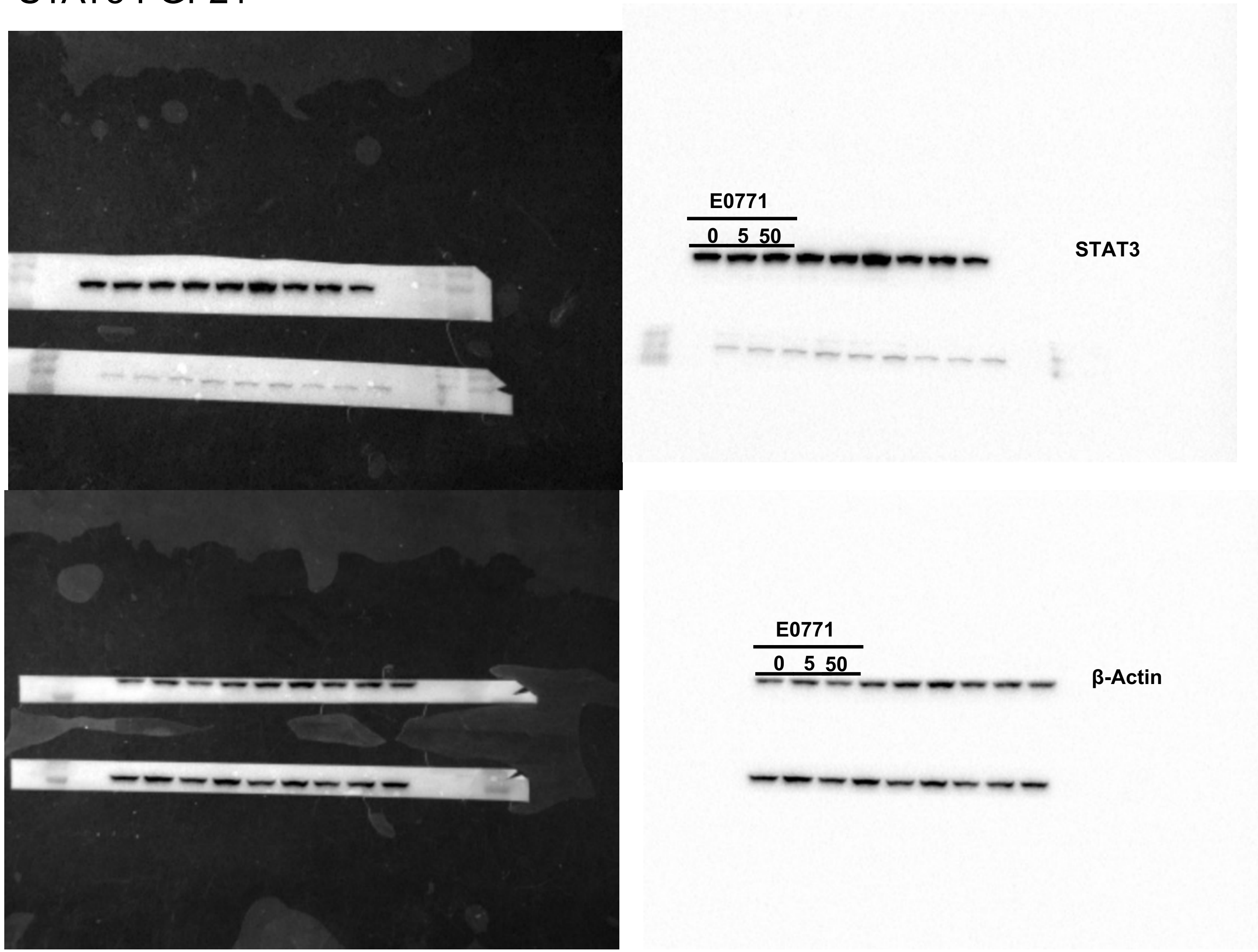

STAT3 FGF21

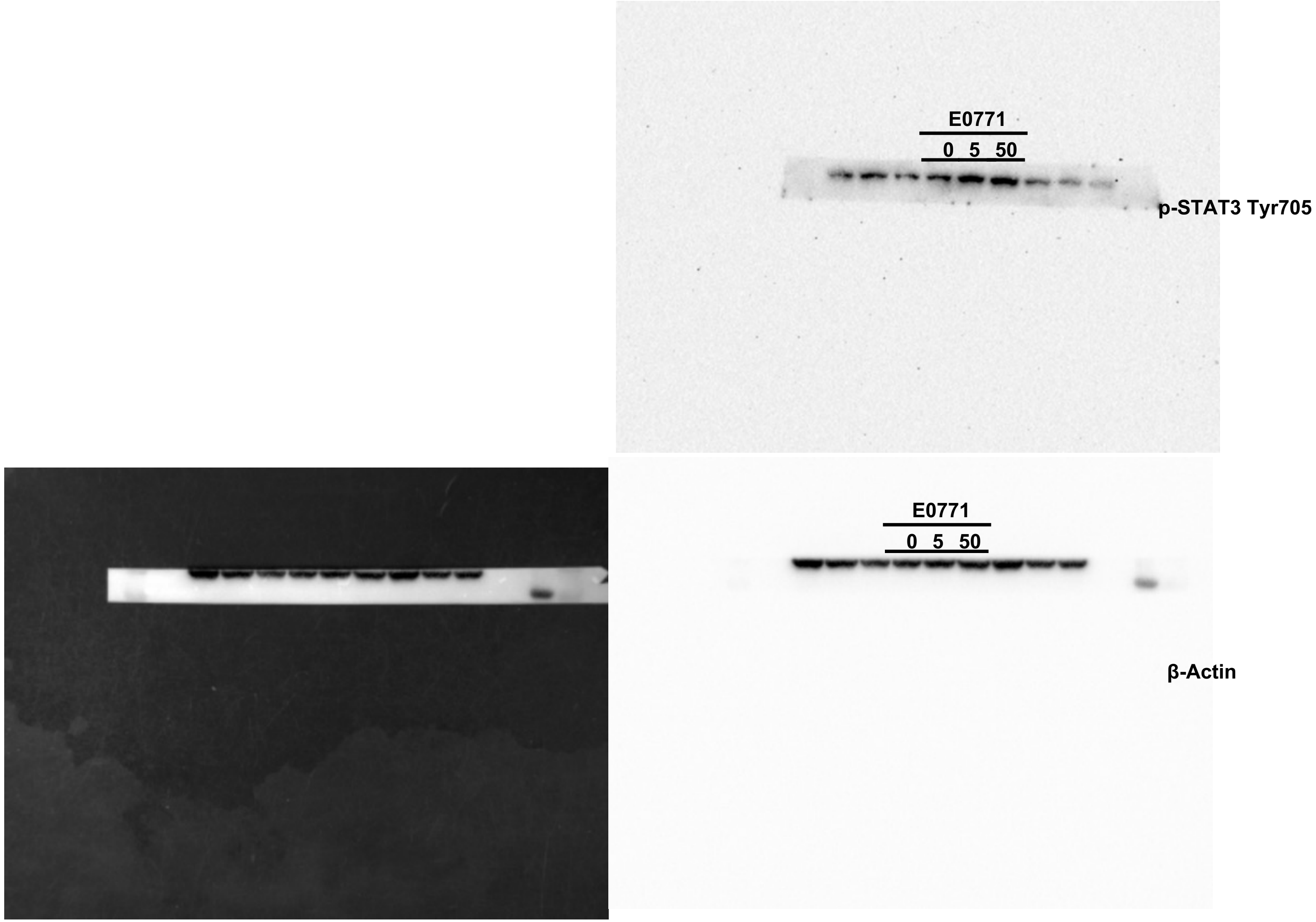

Akt FGF21

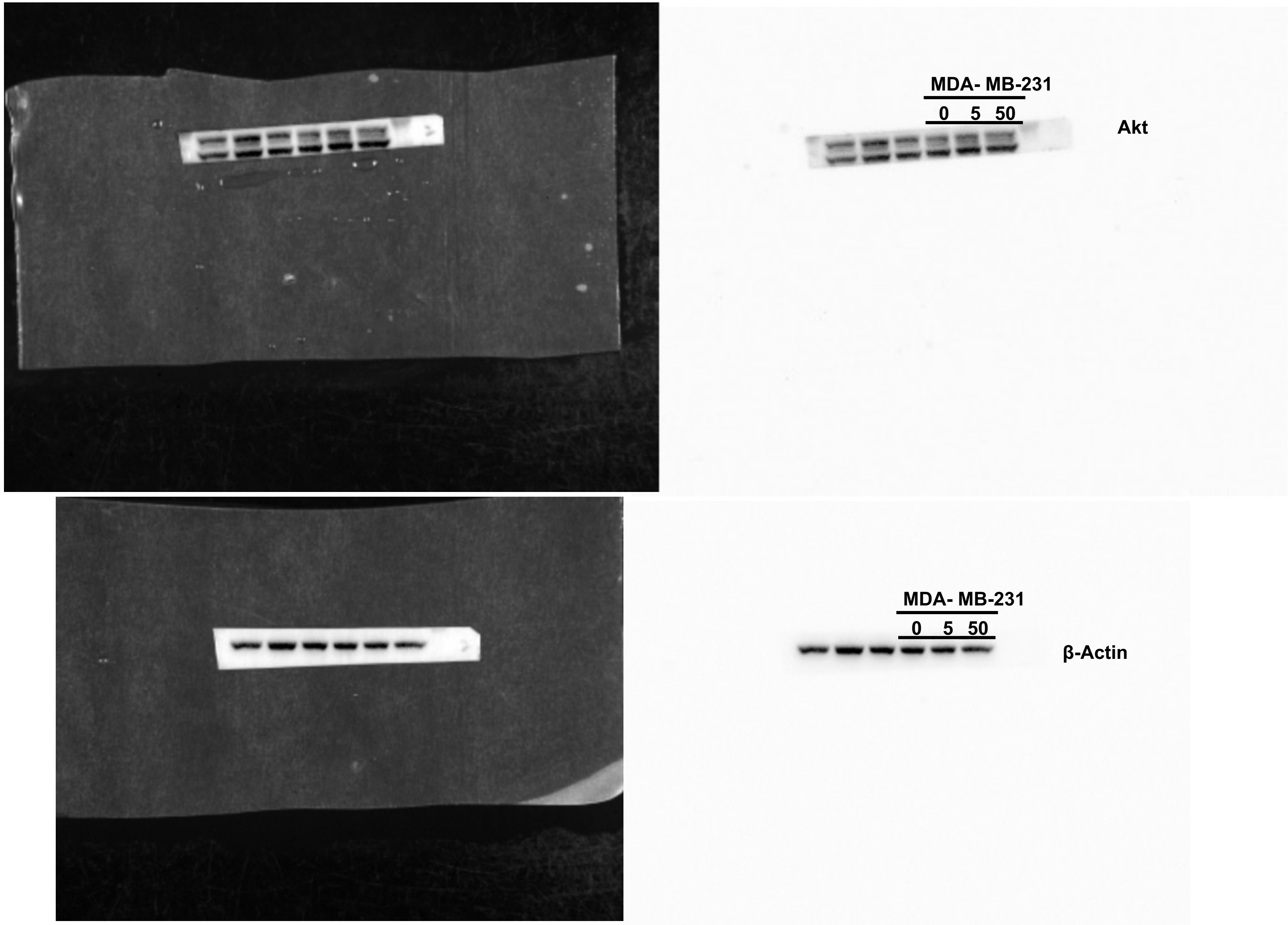

Akt FGF21

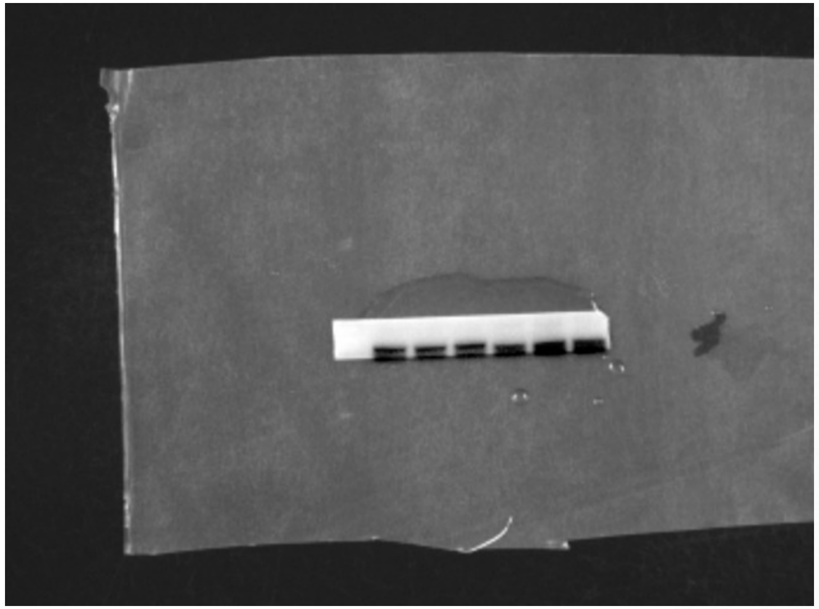

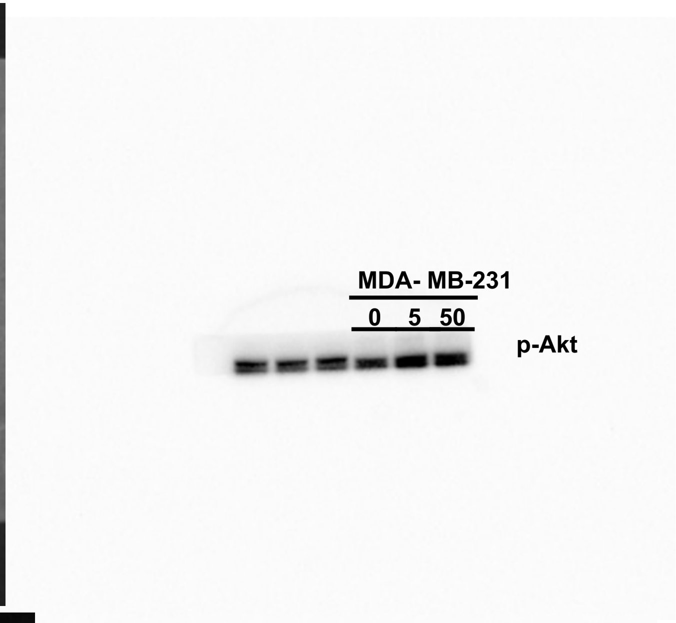

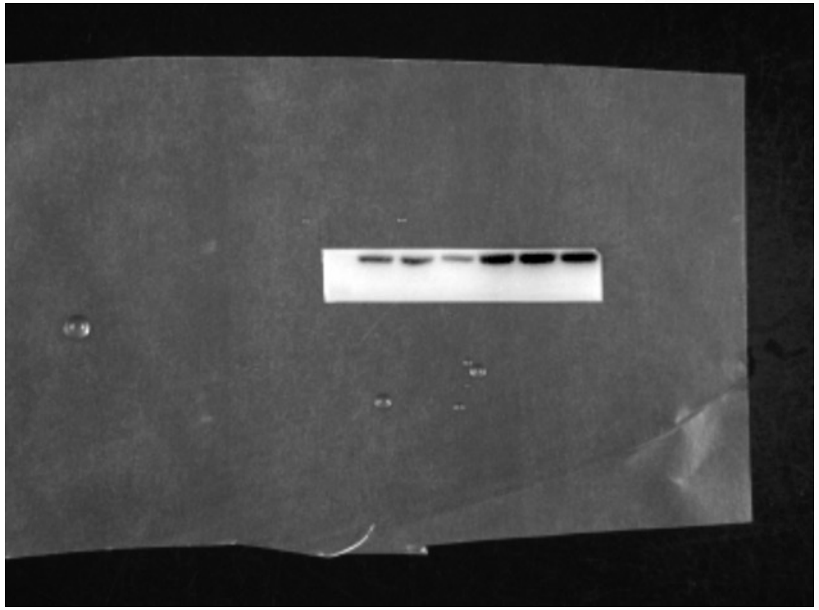

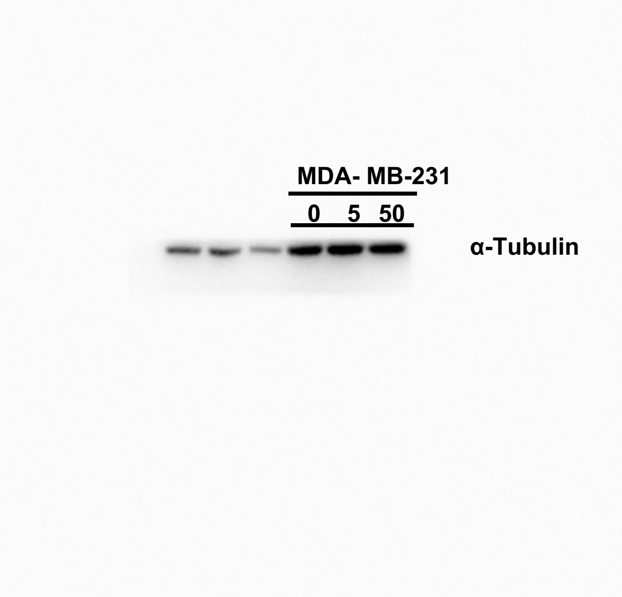

Akt FGF21

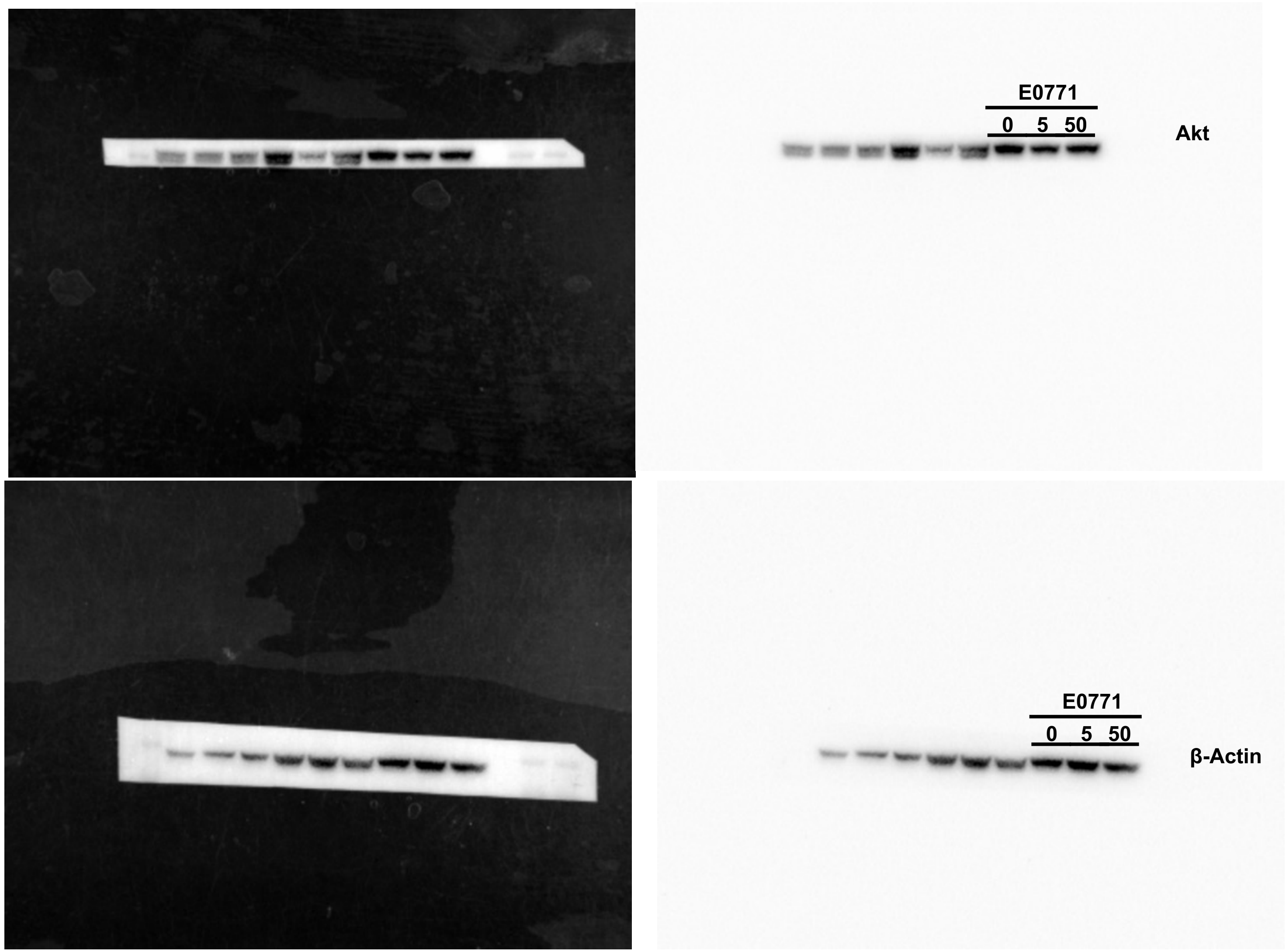

Akt FGF21

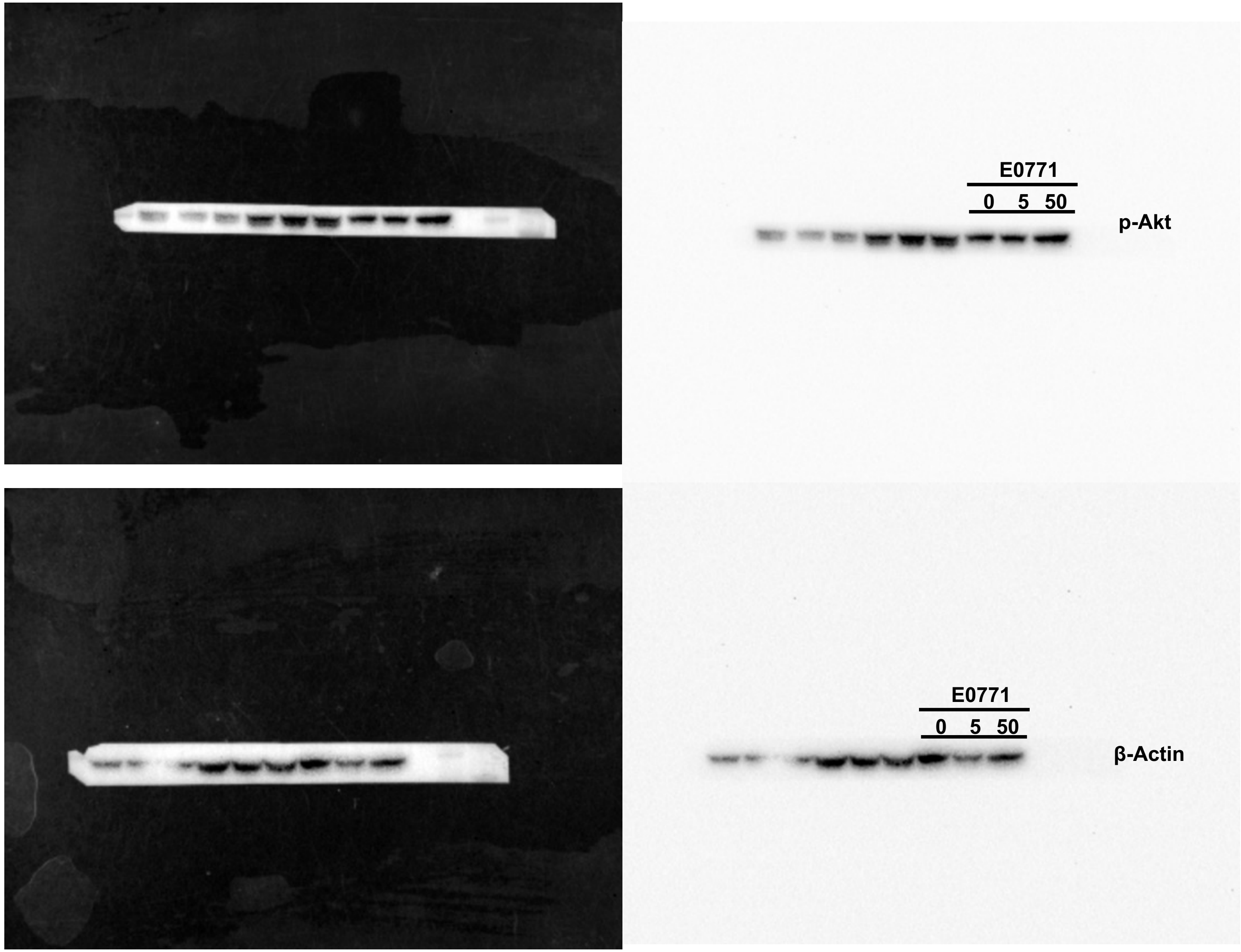

Akt FGF21

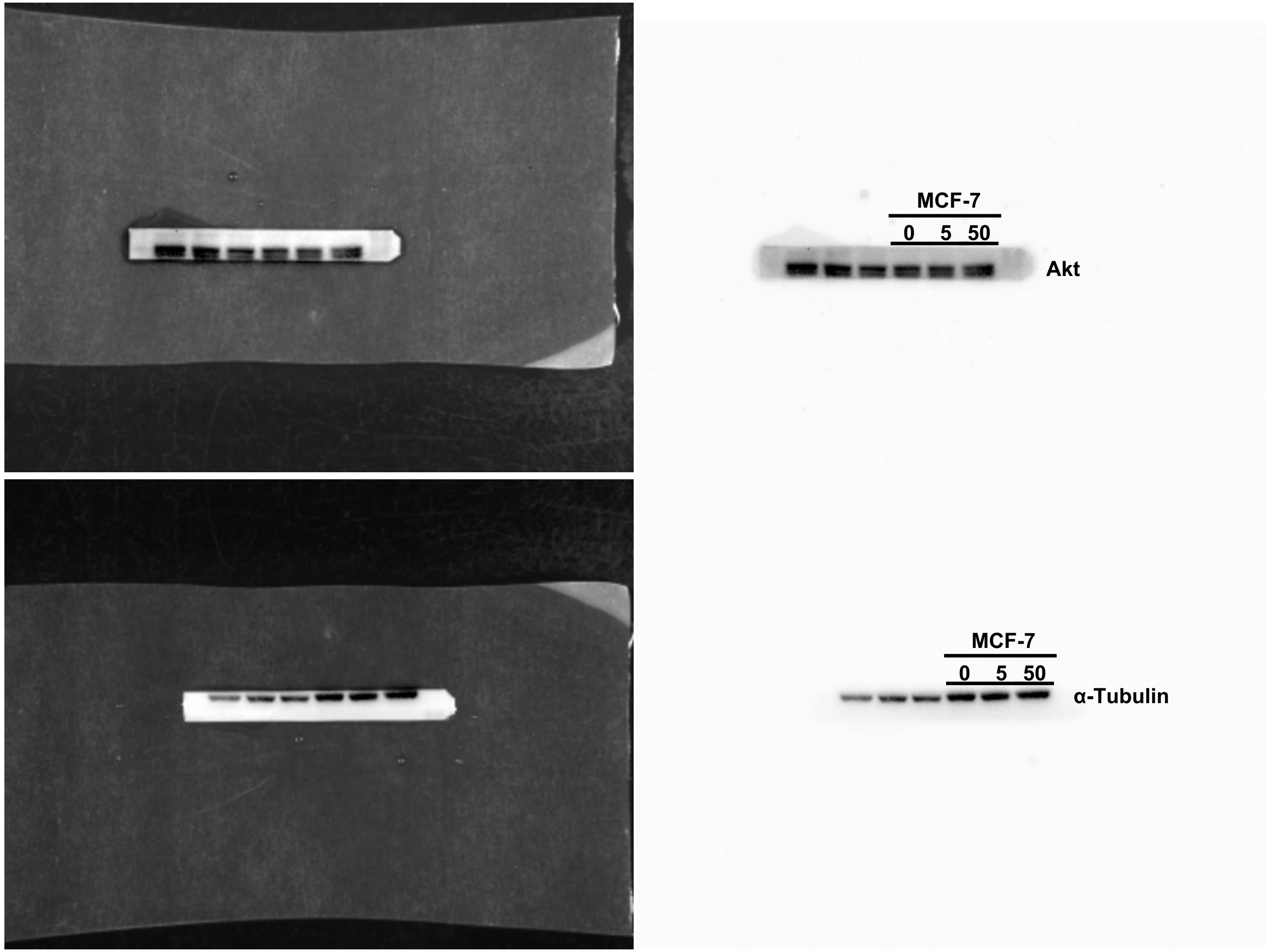

Akt FGF21

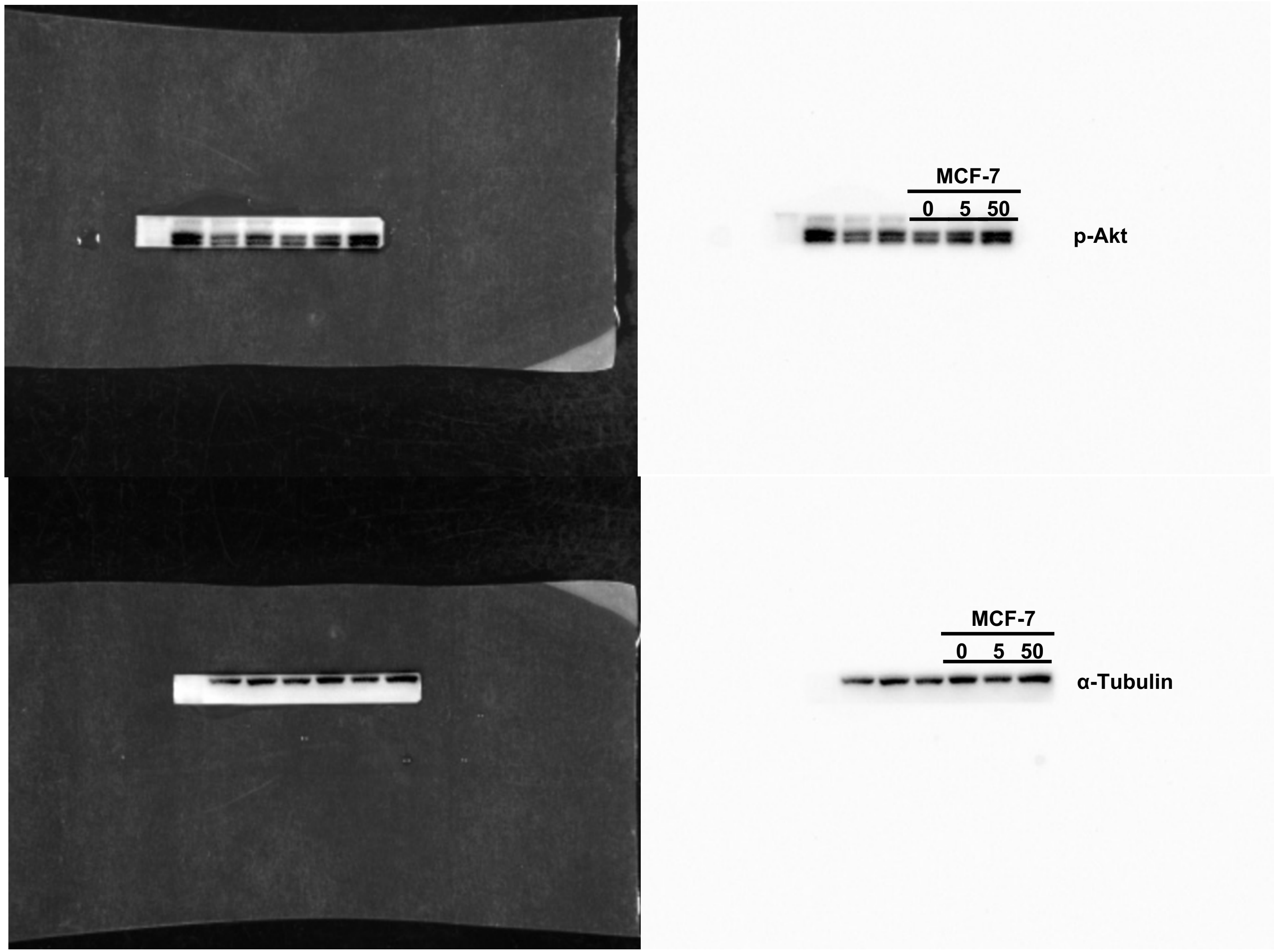

Akt FGF21

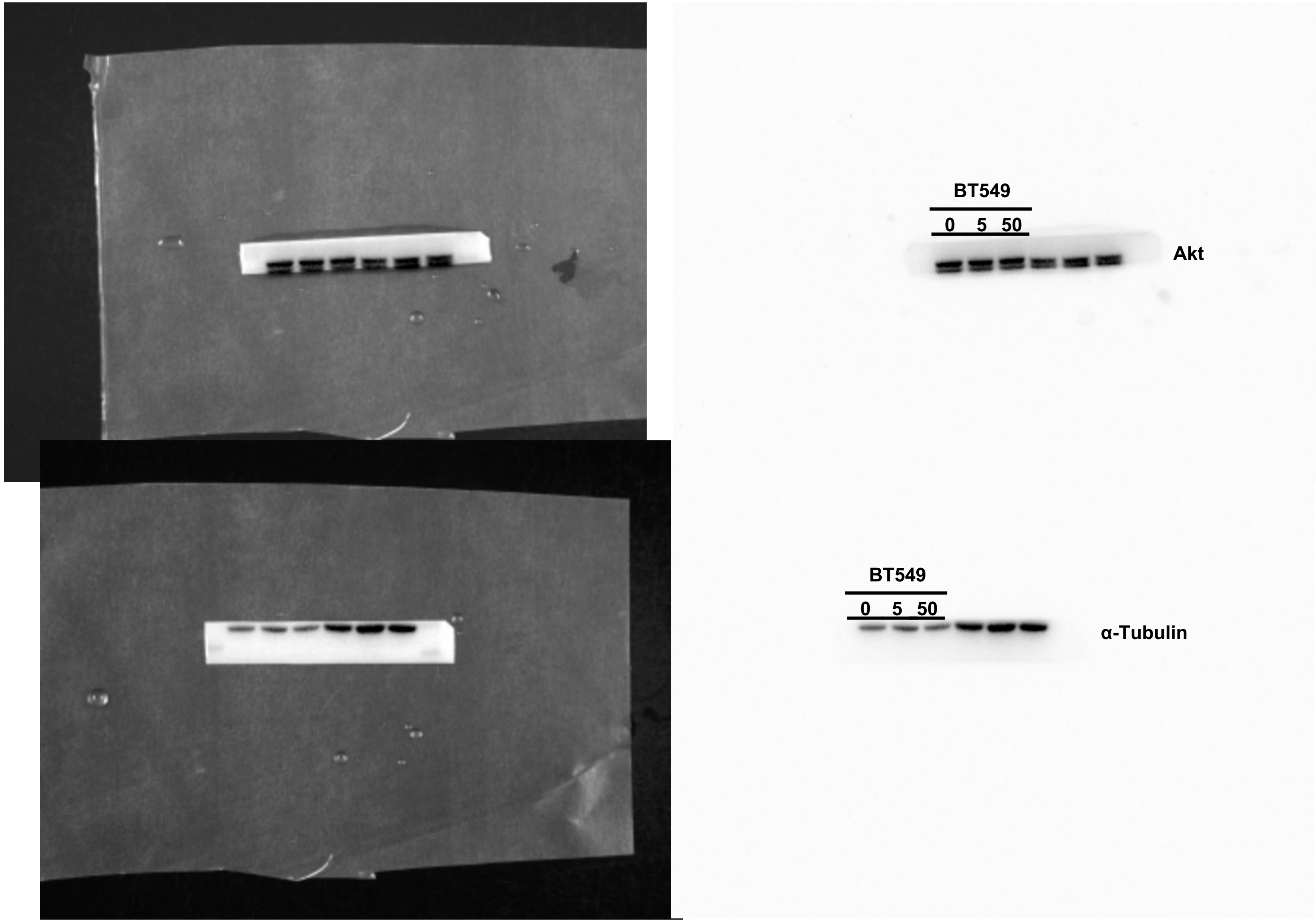

Akt FGF21

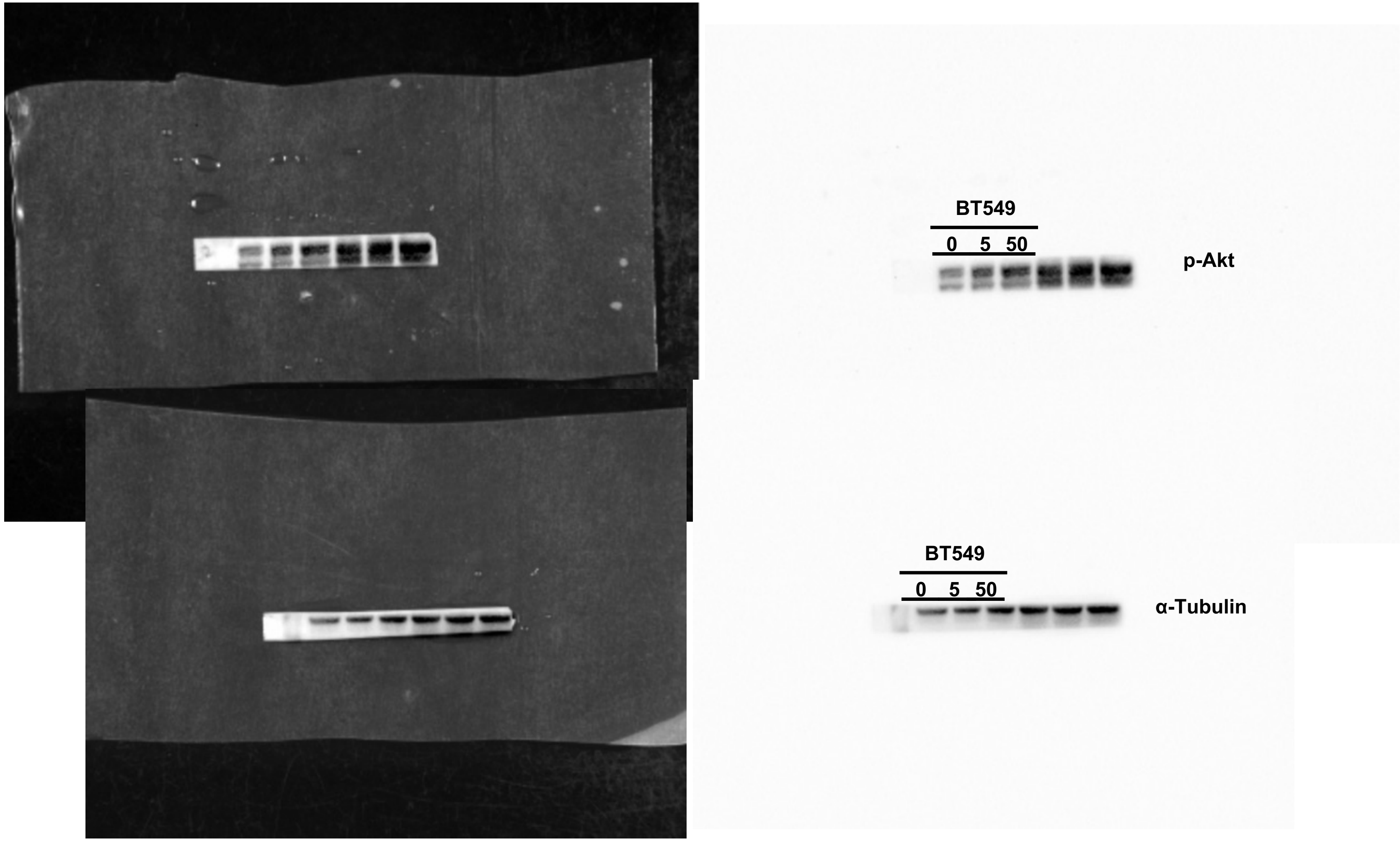

Bcl-2 FGF21

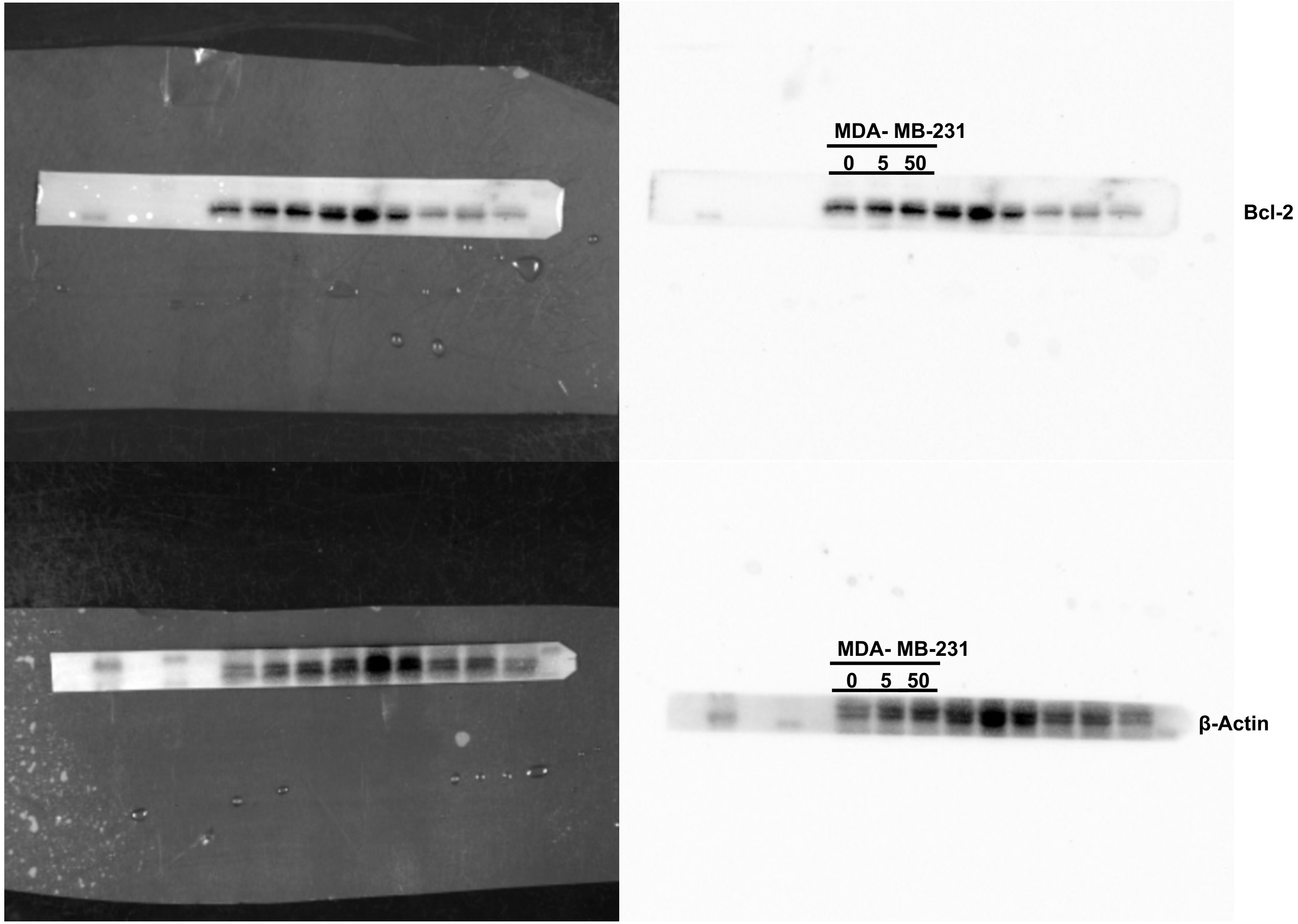

Bcl-2 FGF21

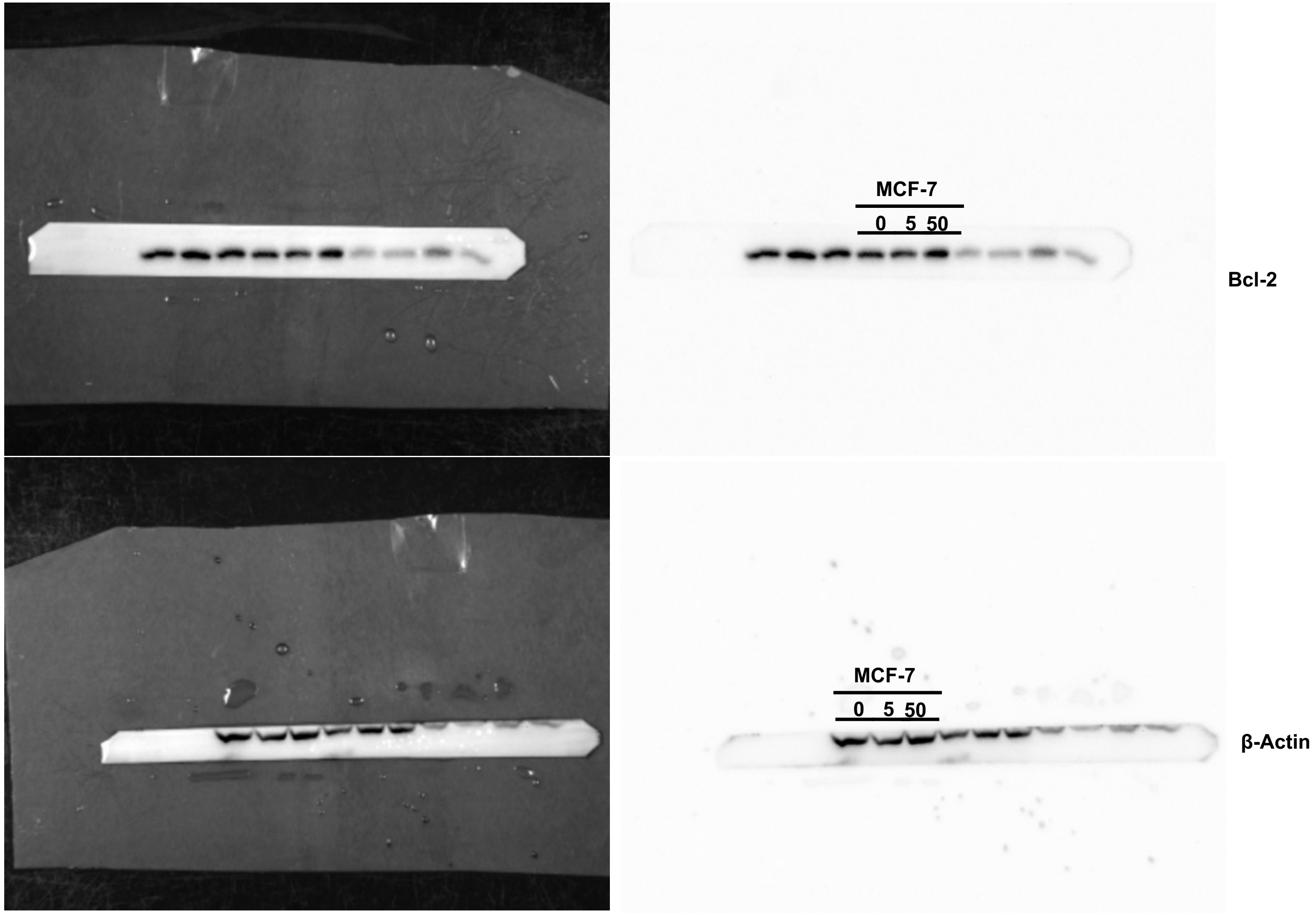

Bcl-2 FGF21

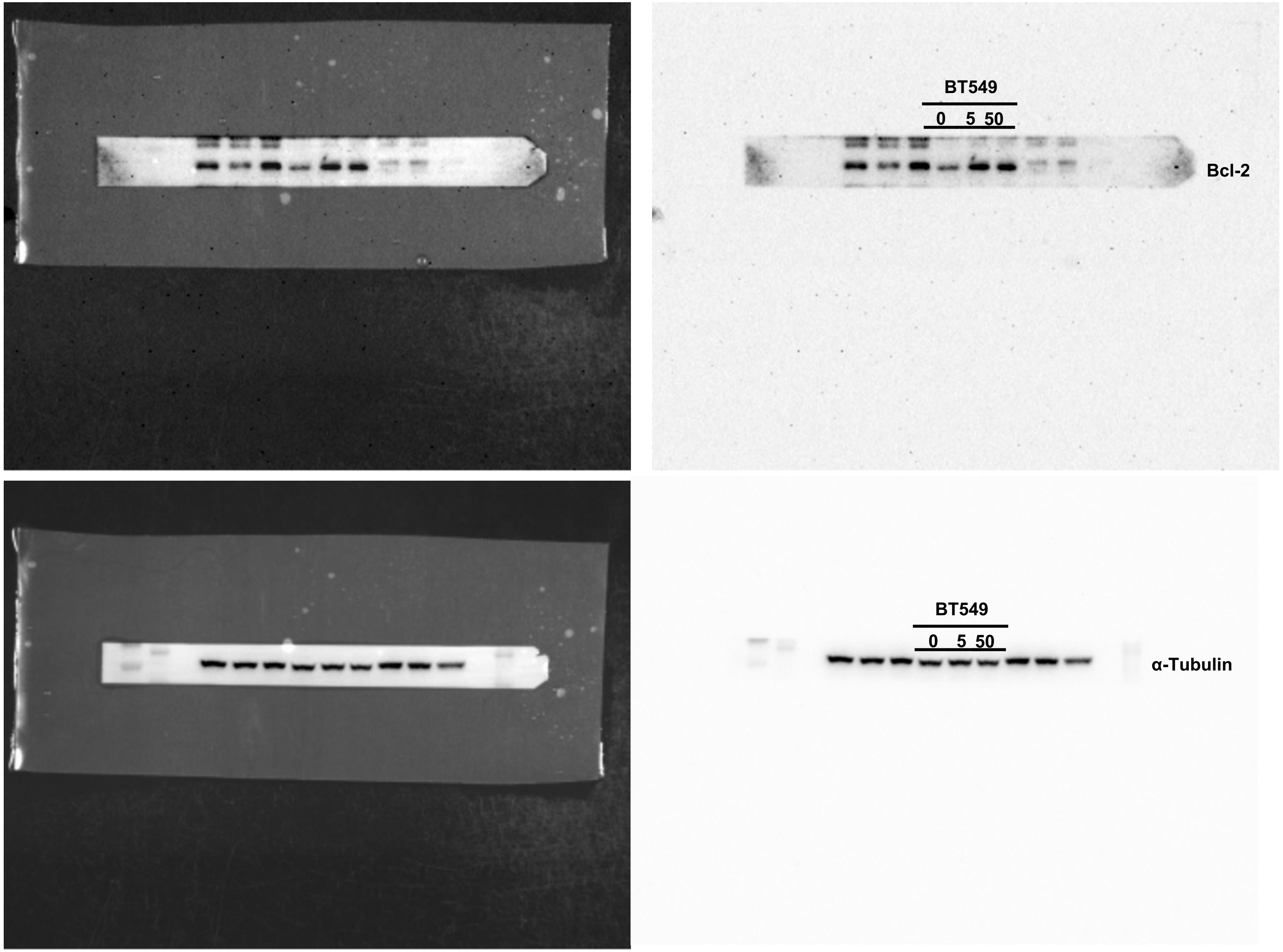

Bcl-2 FGF21

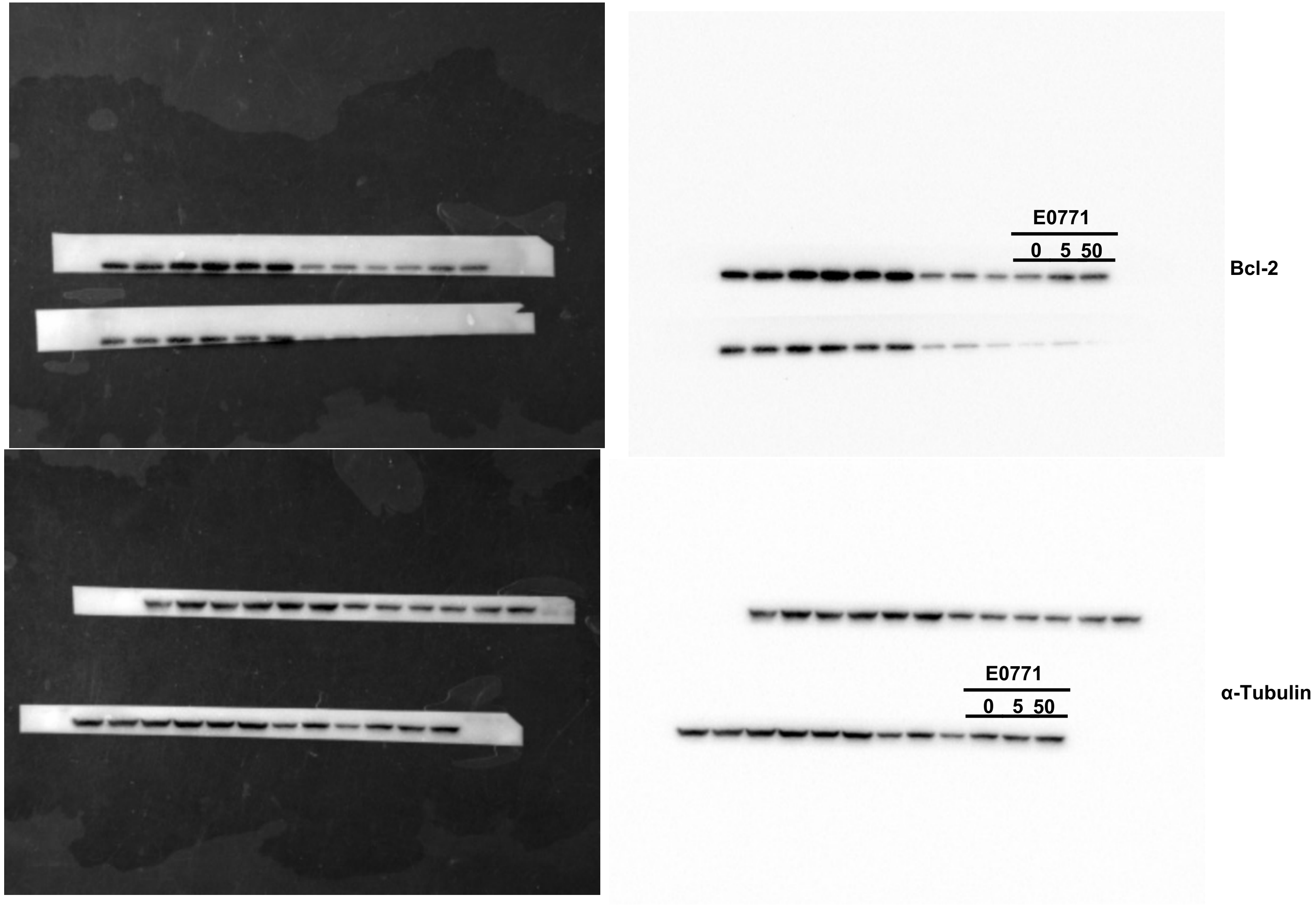

Bax FGF21

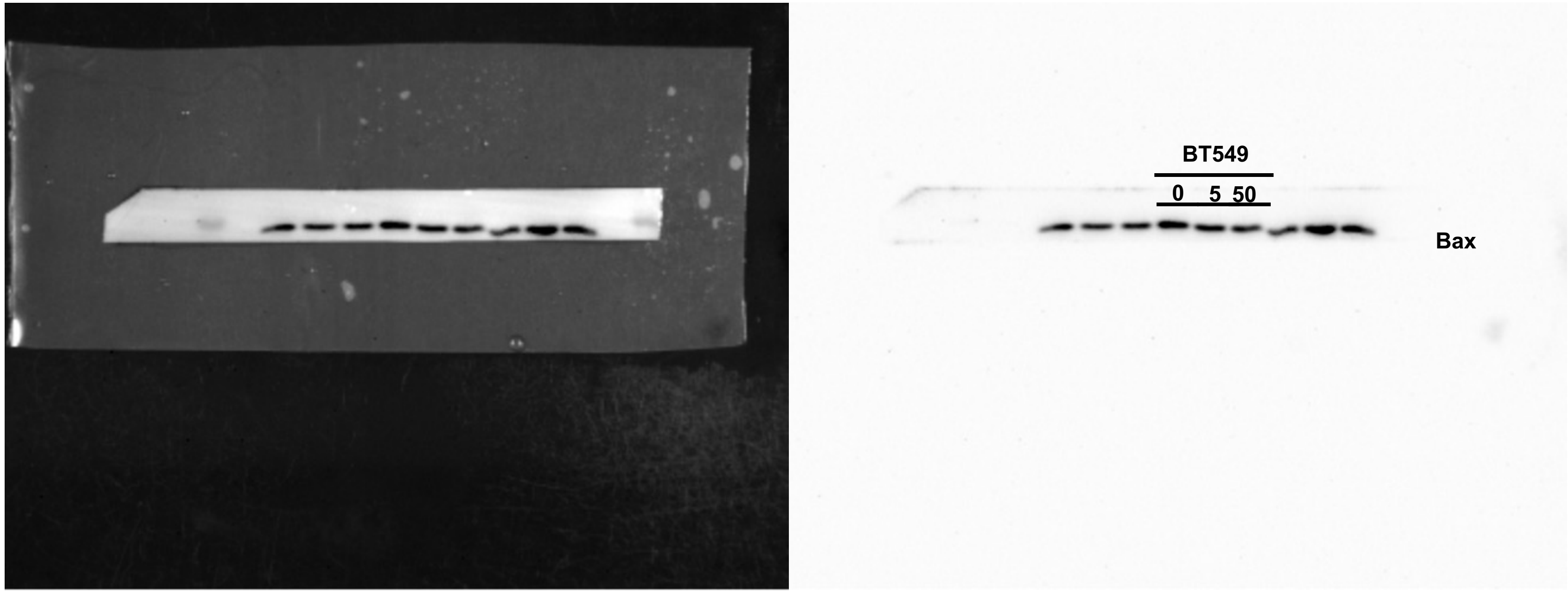

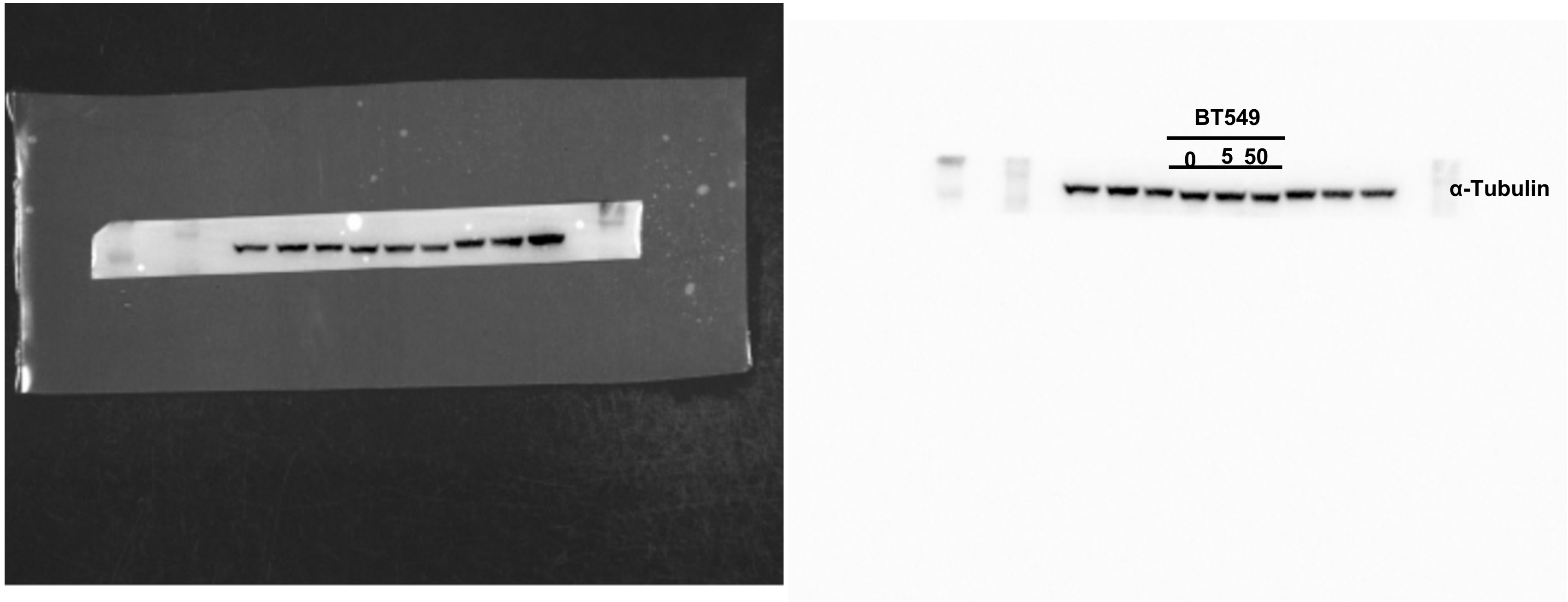

Bax FGF21

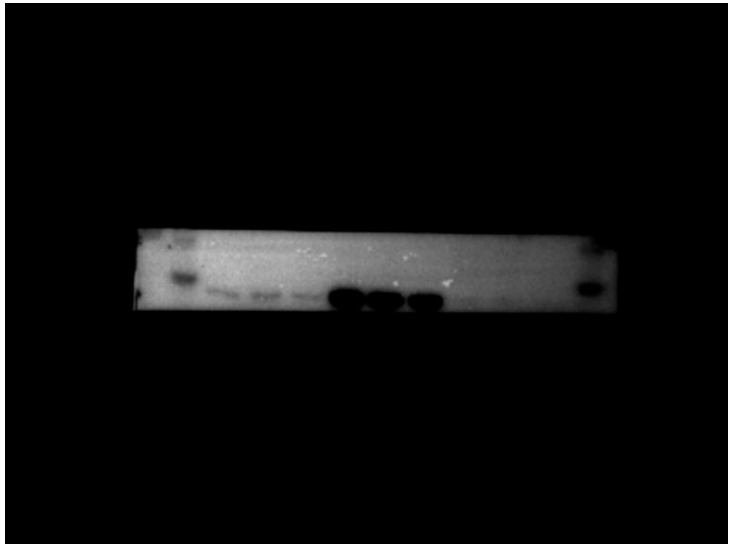

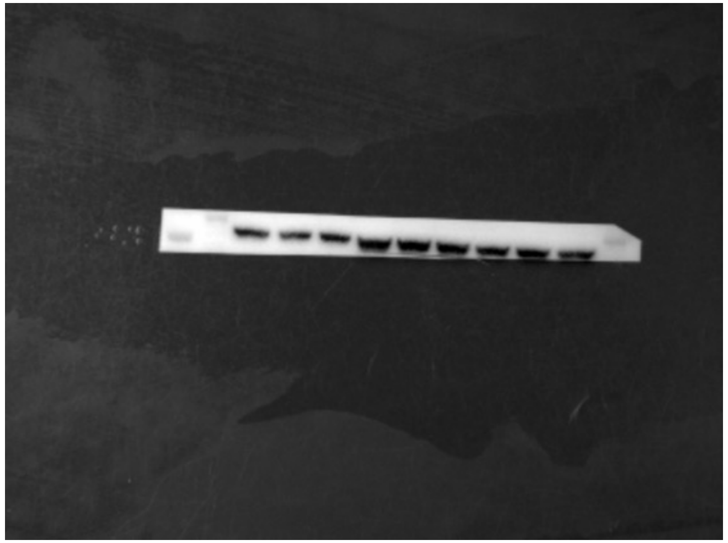

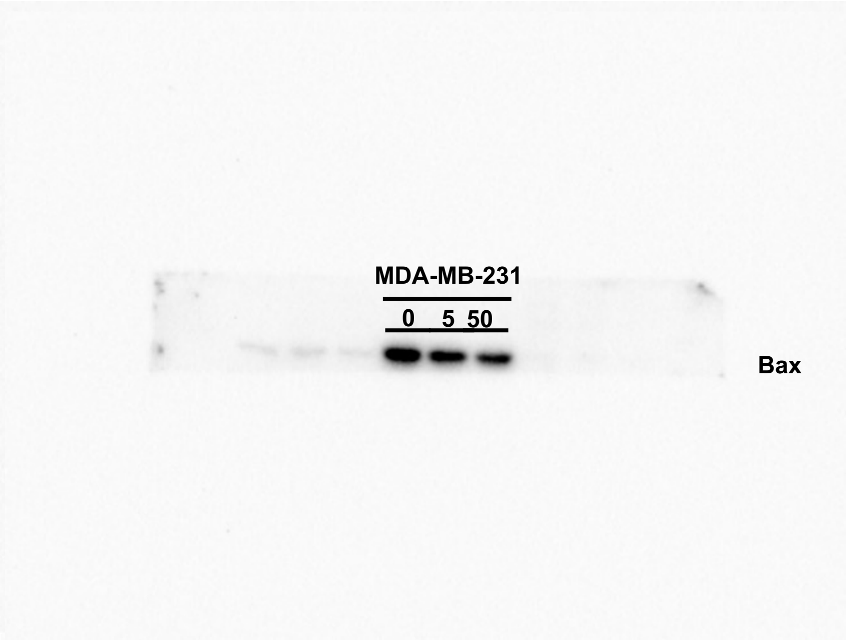

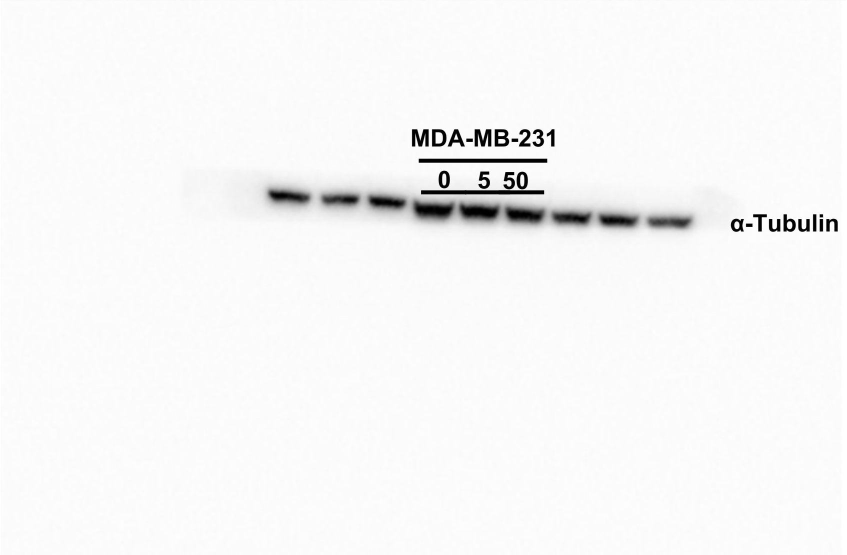

Bax FGF21

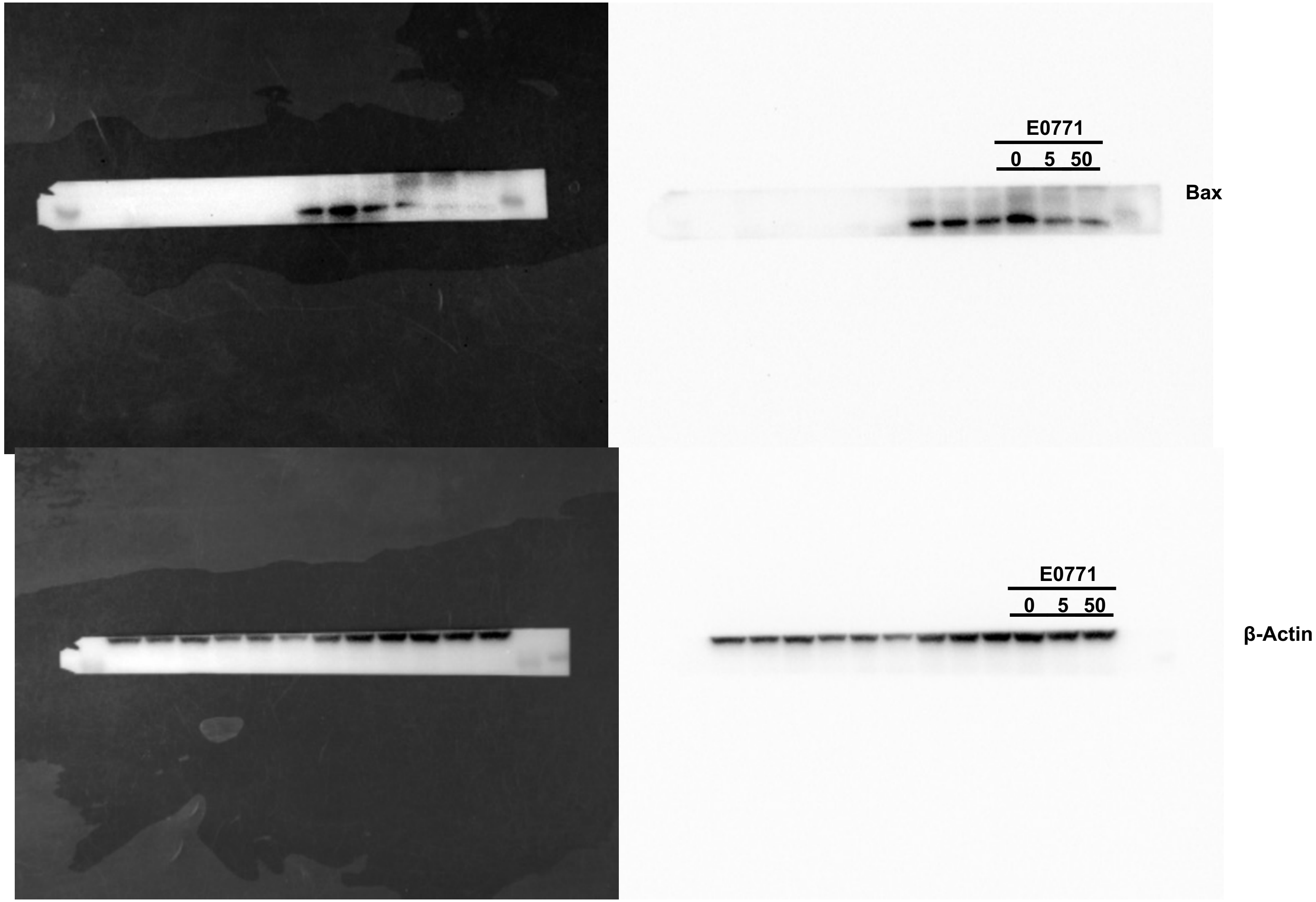

Bax FGF21

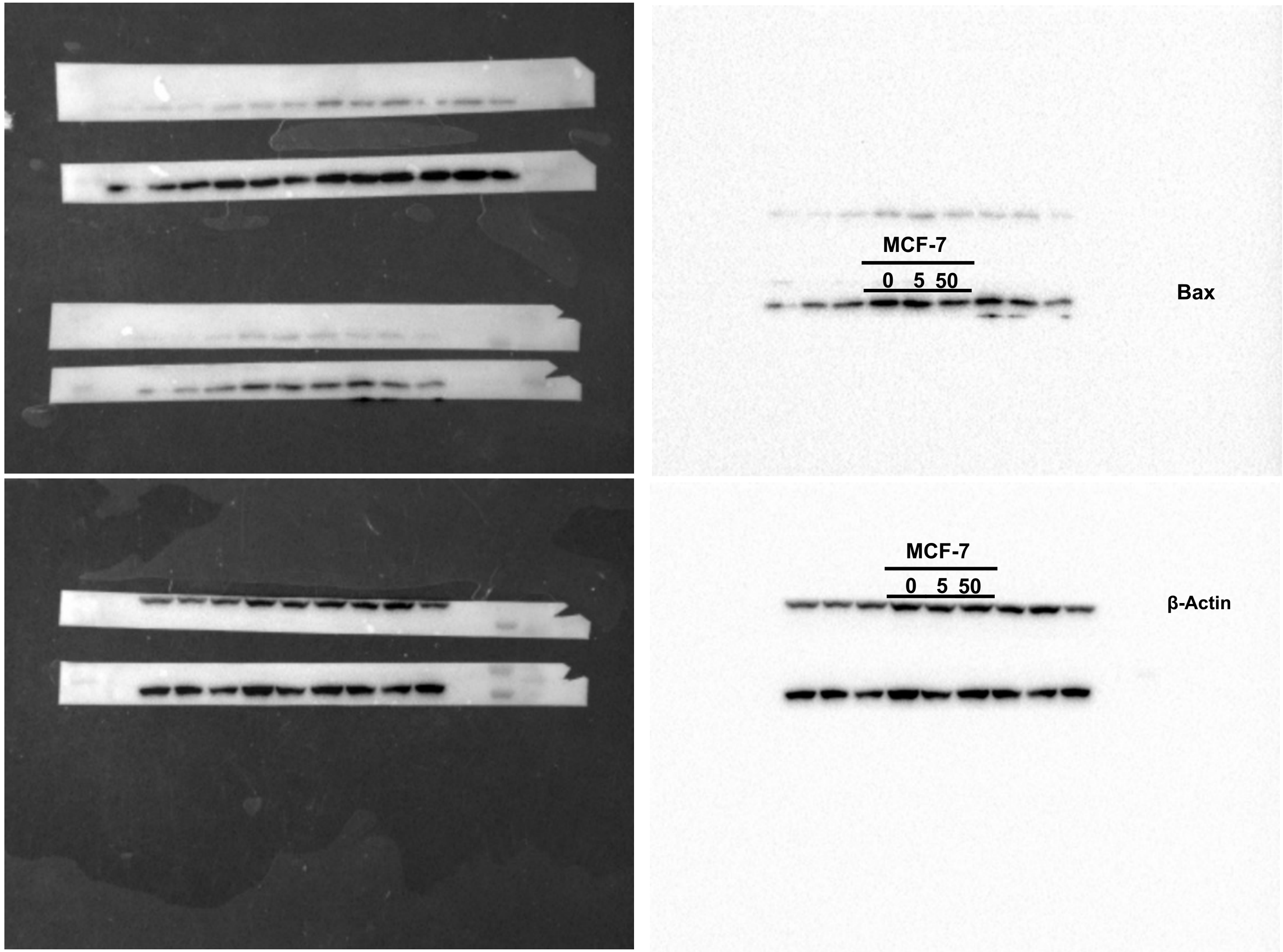

FoxO1 FGF21

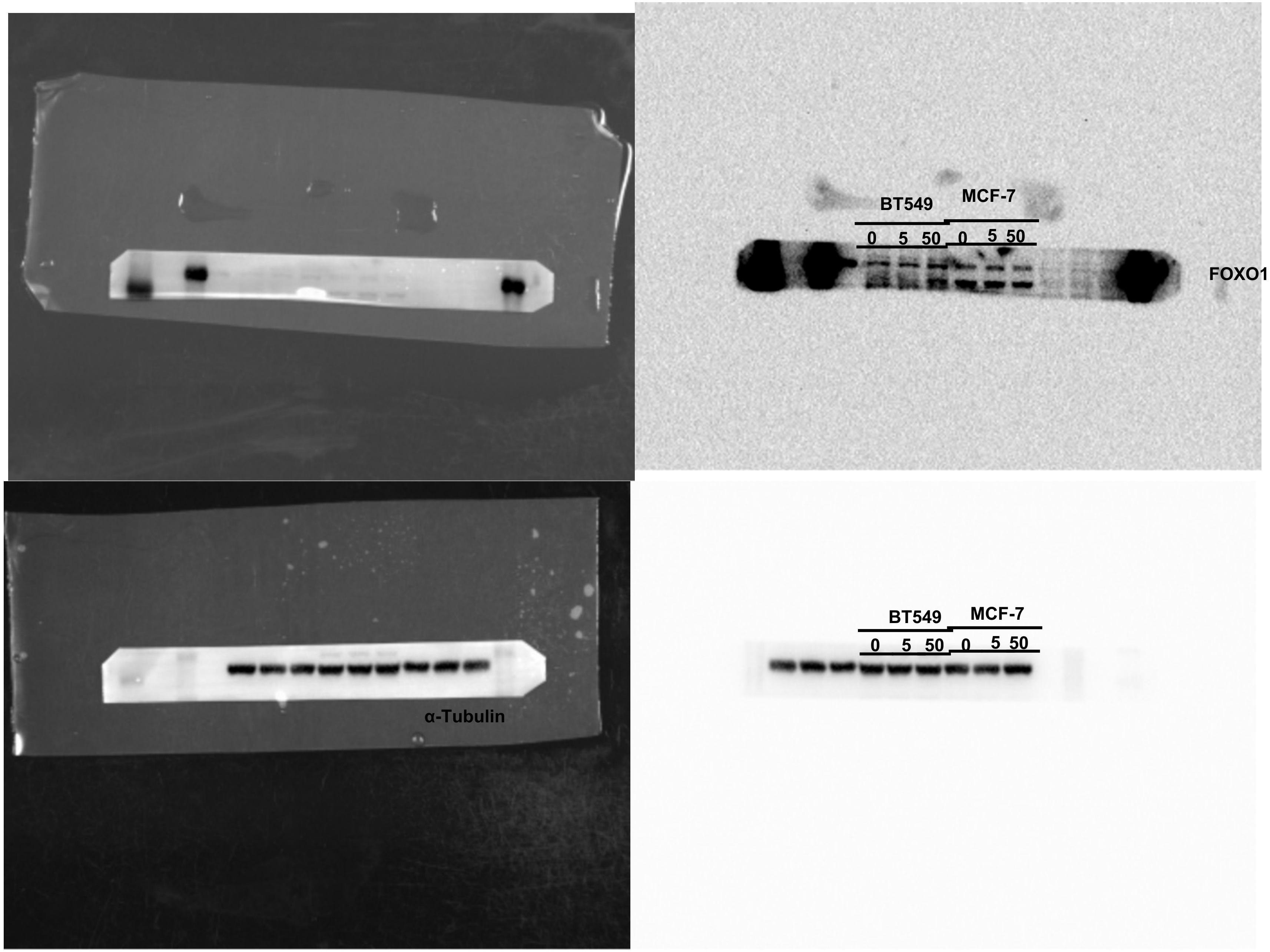

FoxO1 FGF21

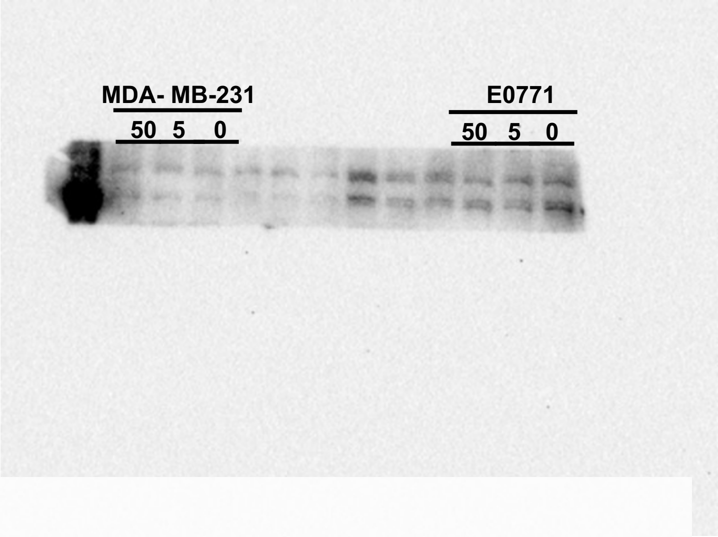

Foxo1

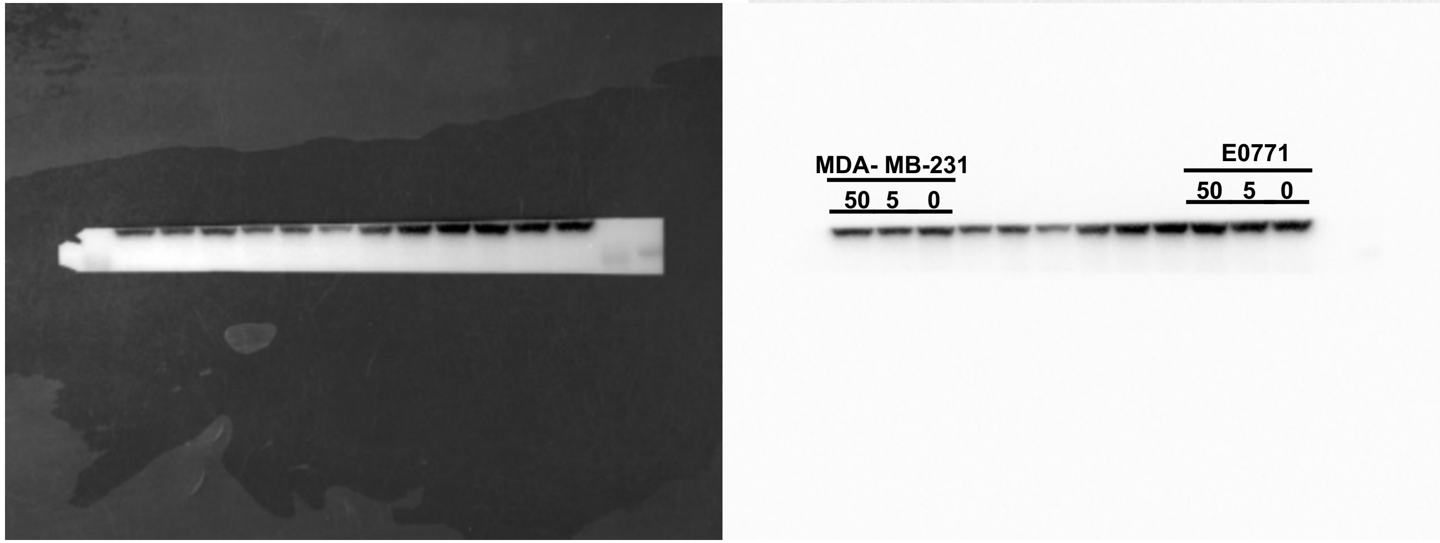

β-Actin

FoxO1 FGF21

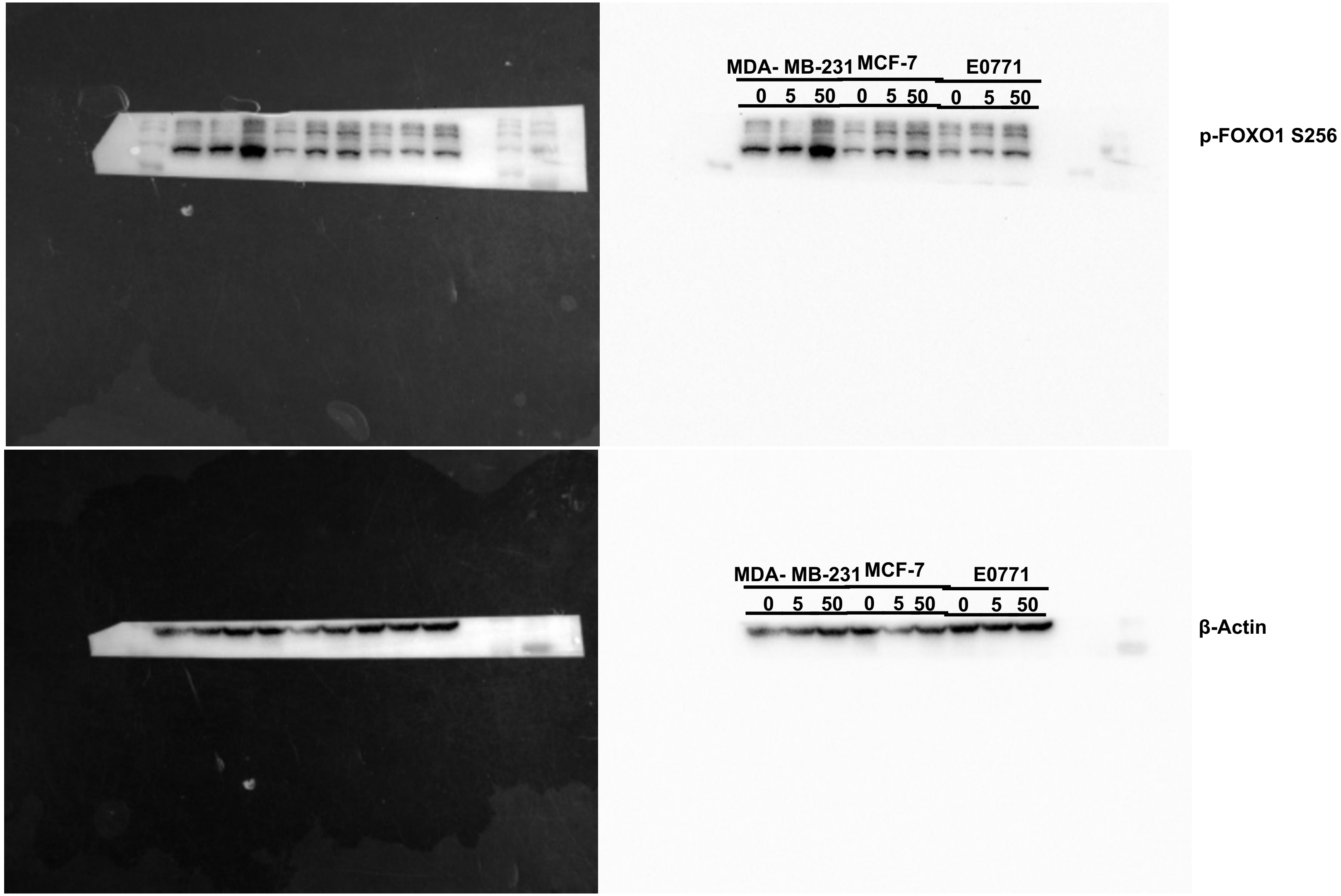

MDA- MB-231
MCF-7
E0771
0 5 50 0 5 50 0 5 50
p-FOXO1 S256
MDA- MB-231
MCF-7
E0771
0 5 50 0 5 50 0 5 50
β-Actin

FoxO1 FGF21

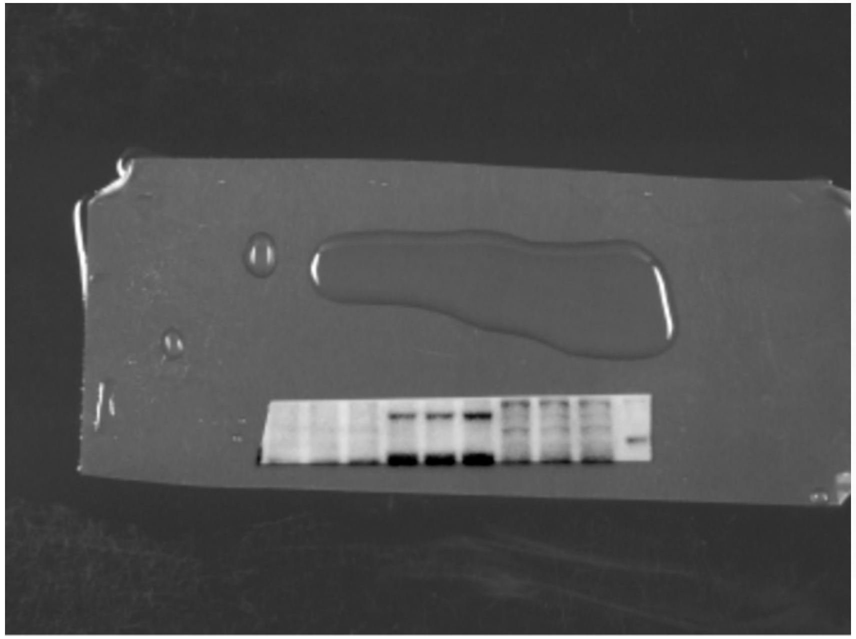

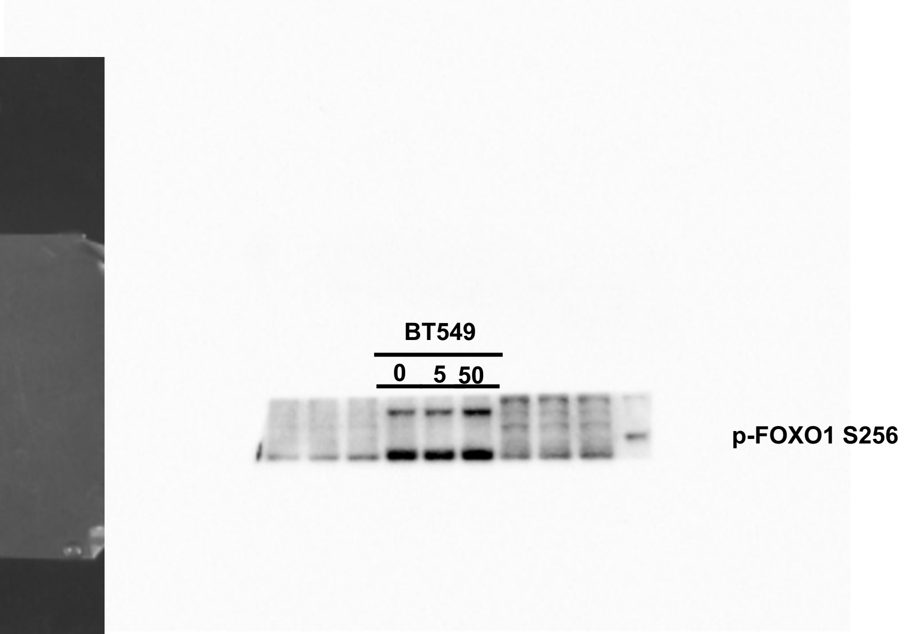

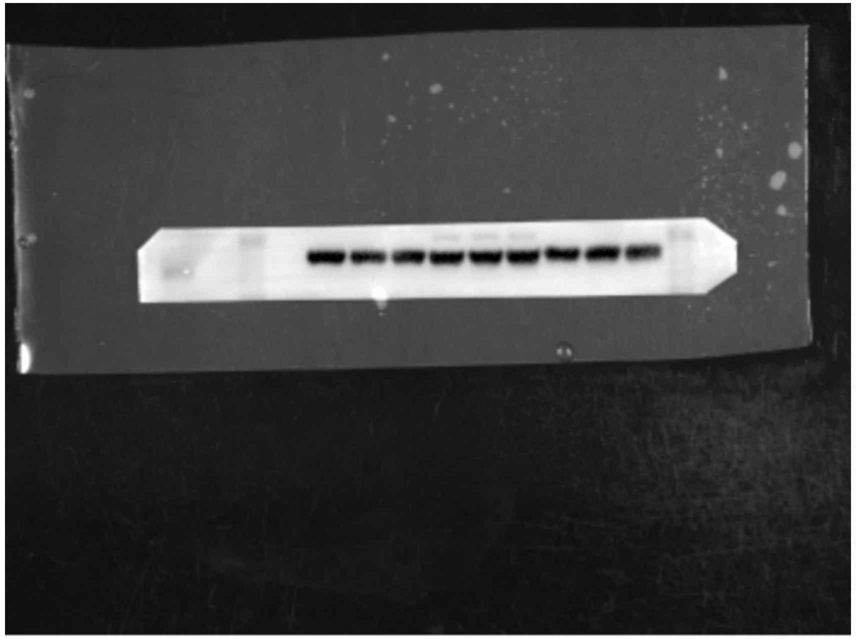

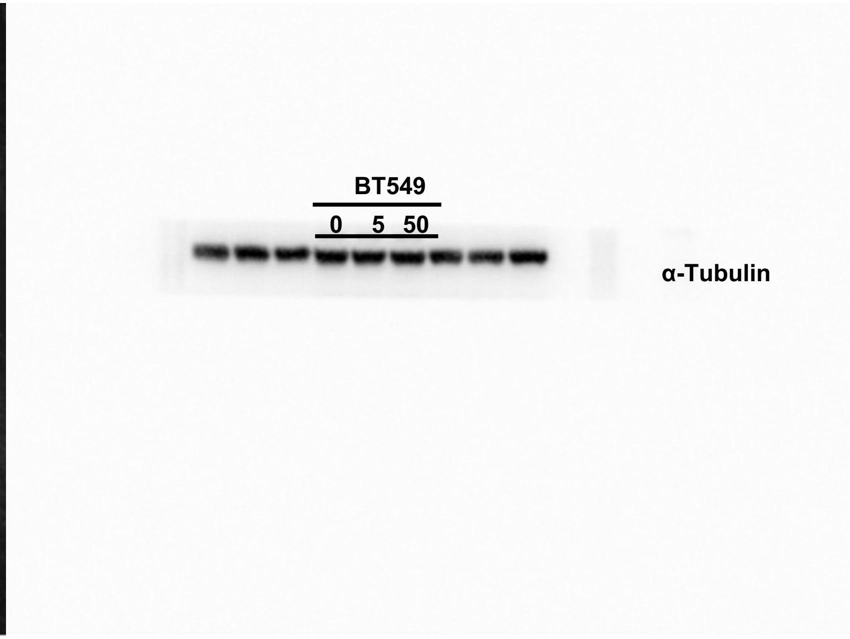

peritumoral injection

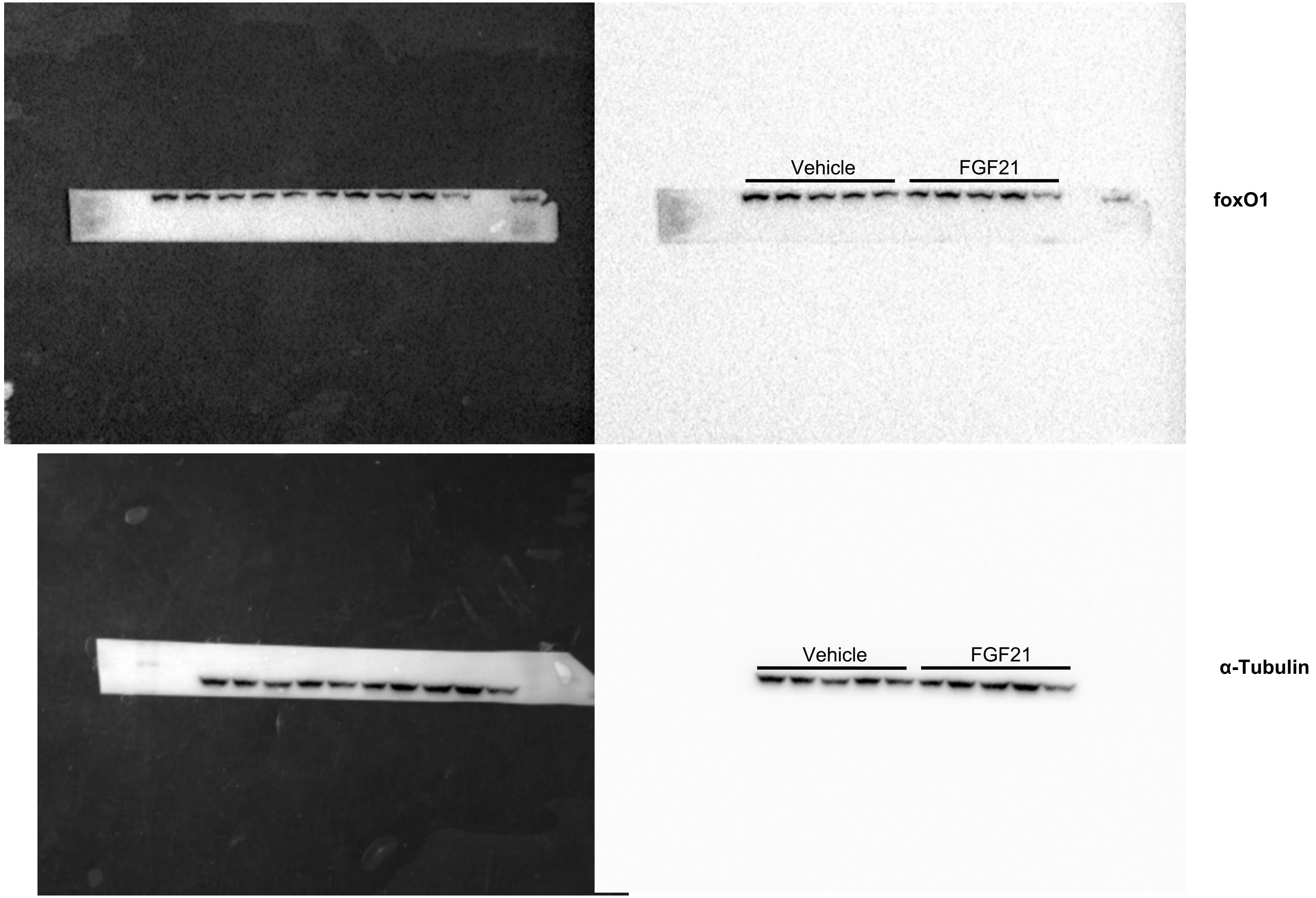

peritumoral injection

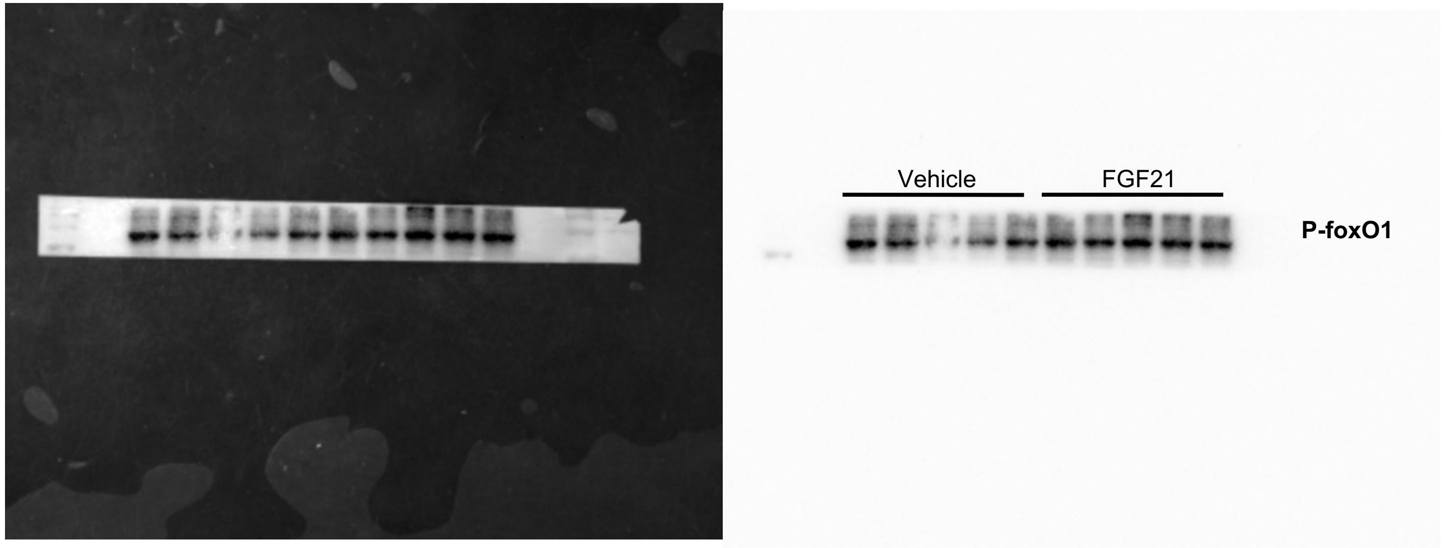

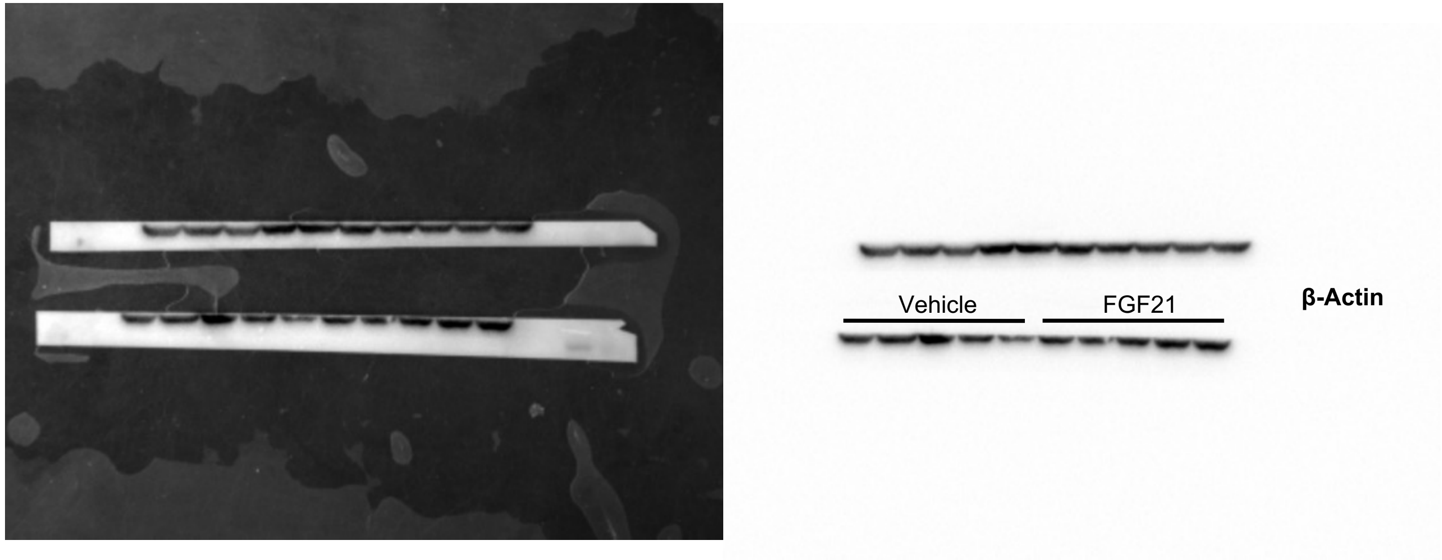

peritumoral injection

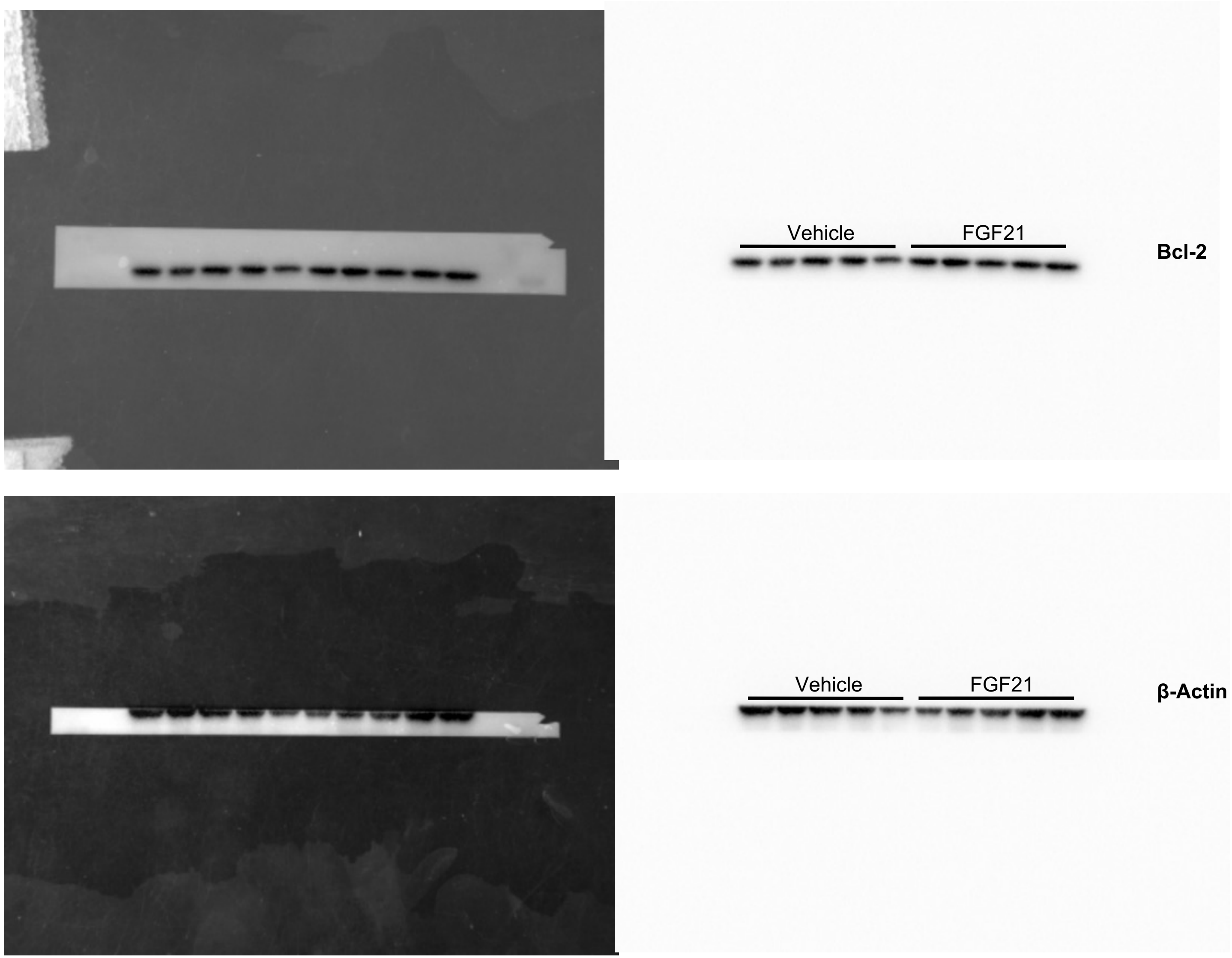

peritumoral injection

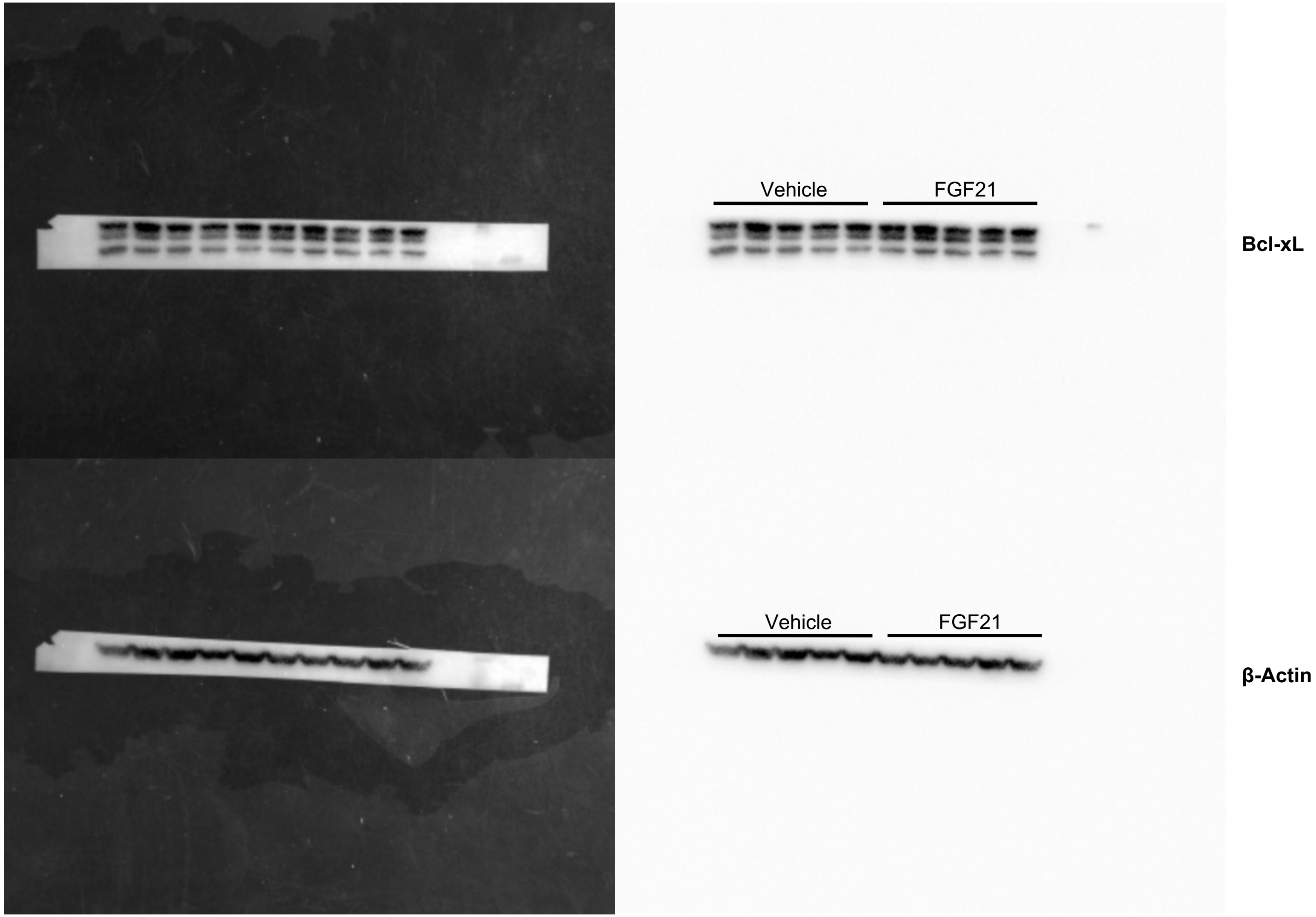

sustained release

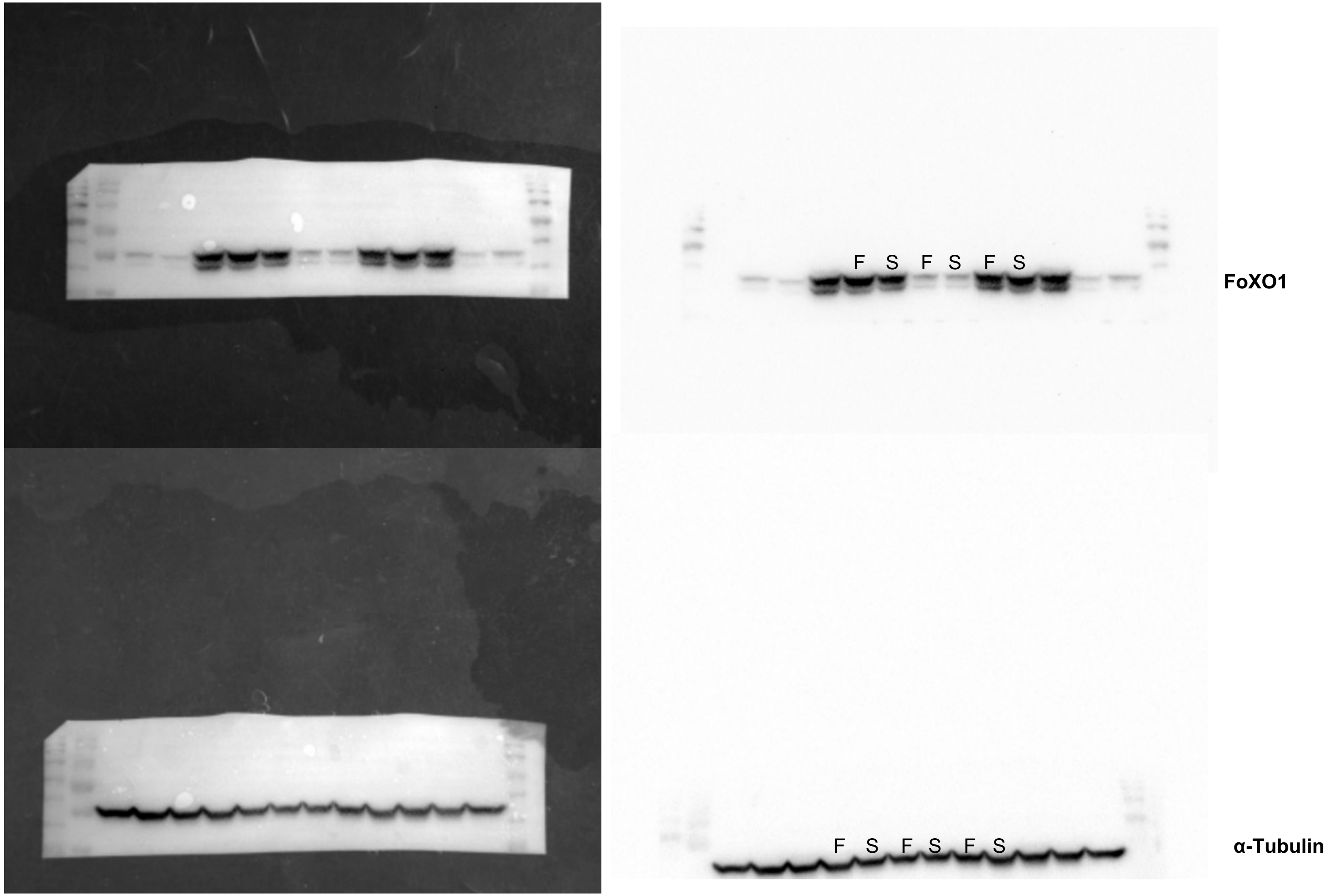

sustained release

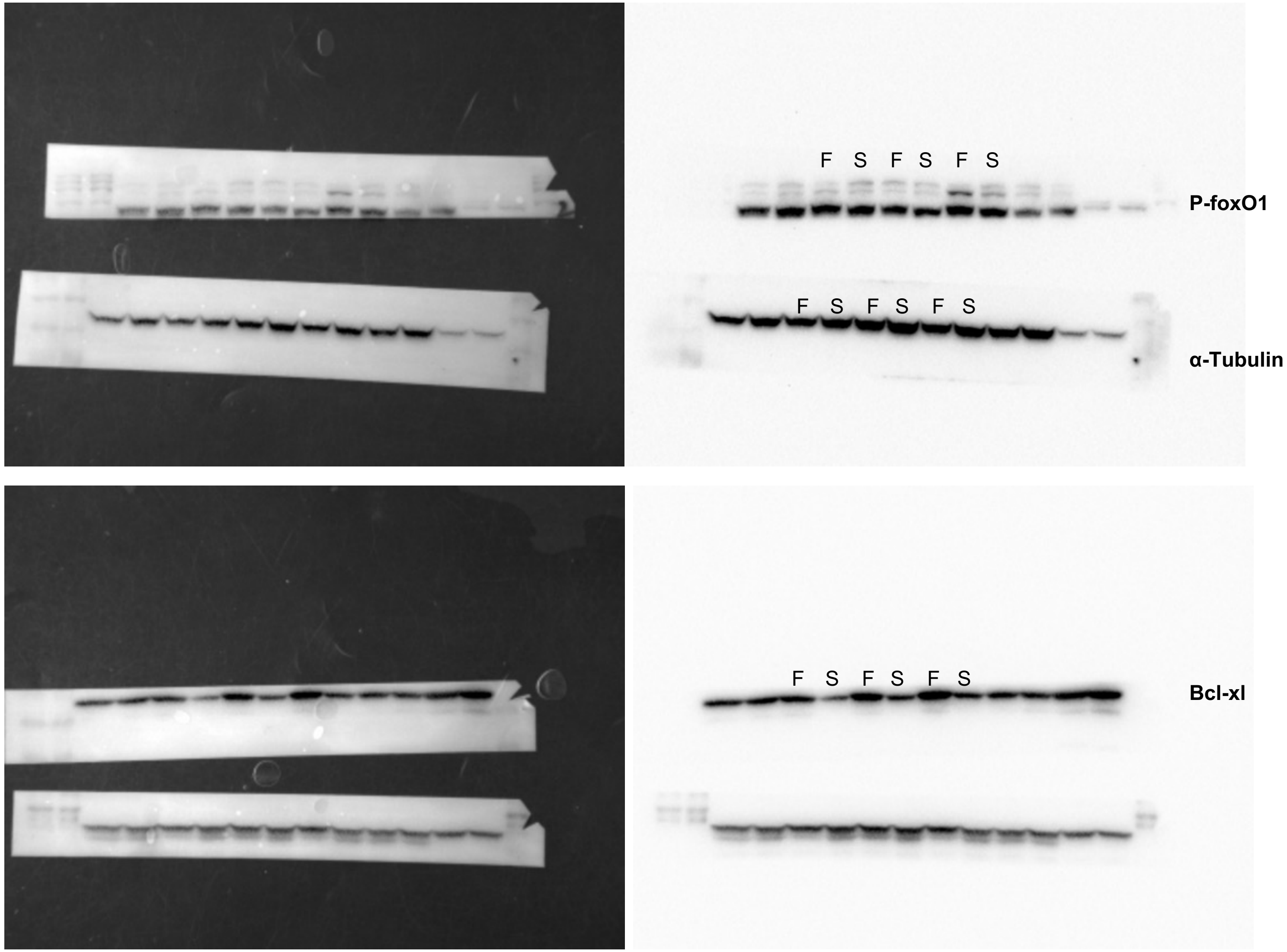

sustained release

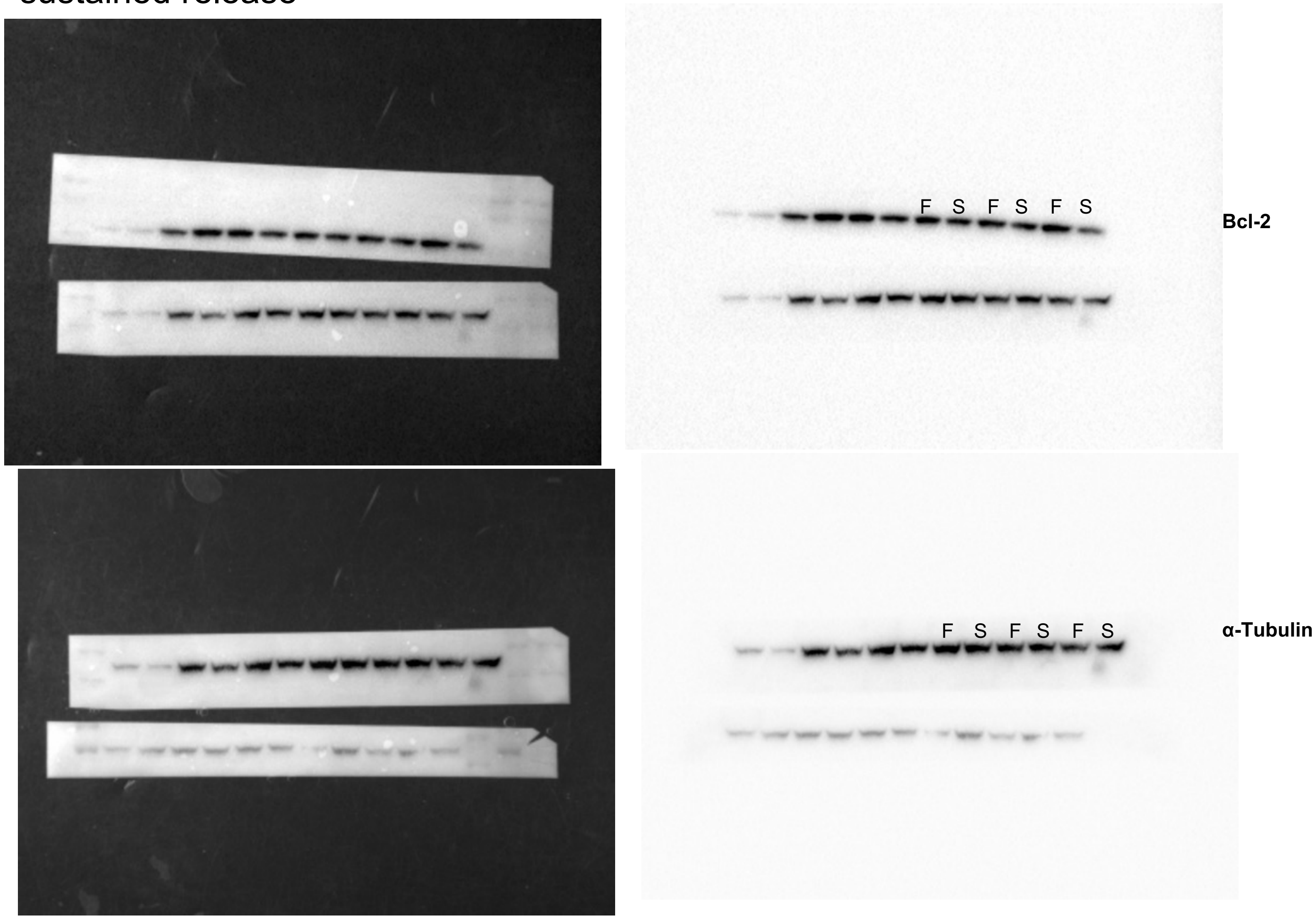

Bcl-2 D+F

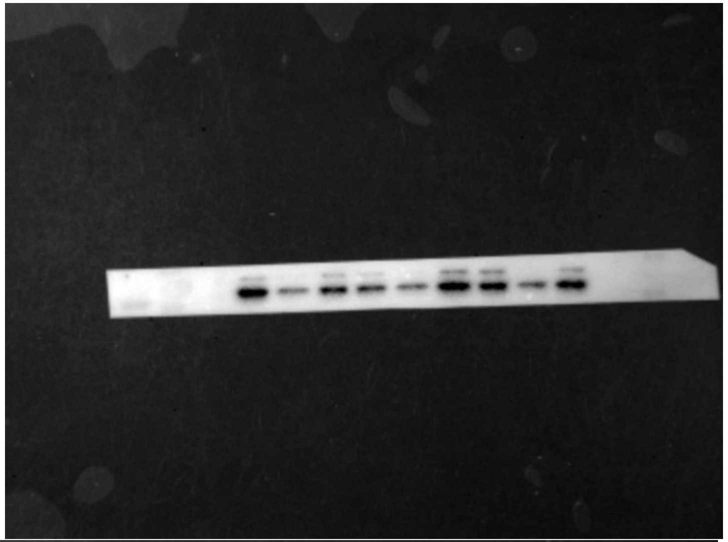

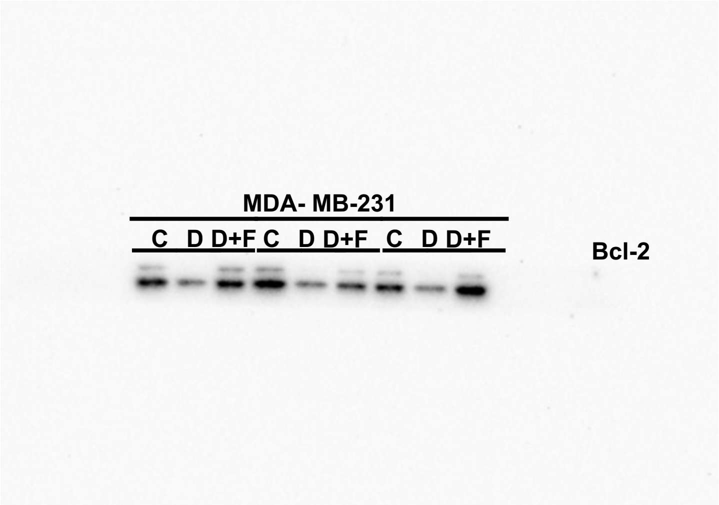
MDA- MB-231
C D D+F C D D+F C D D+F
Bcl-2

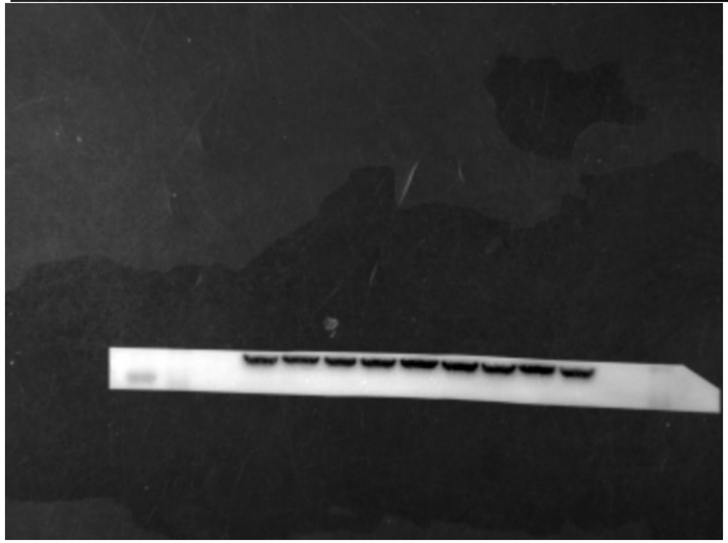

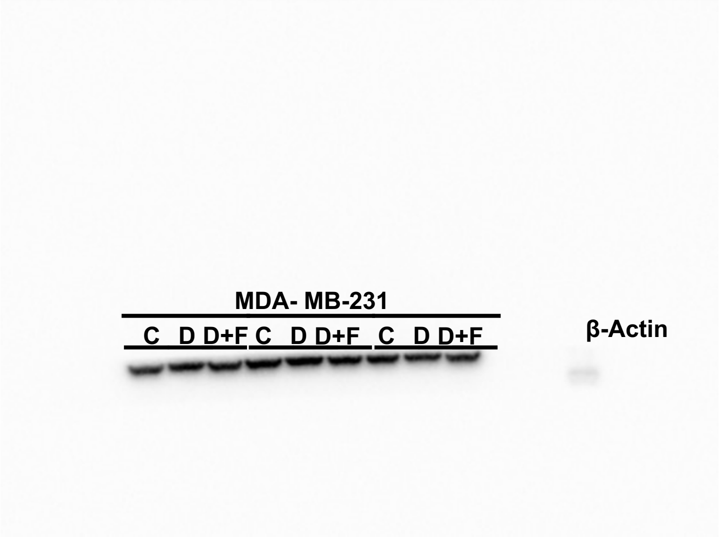
MDA- MB-231
C D D+F C D D+F C D D+F
β-Actin

Bcl-2 D+F

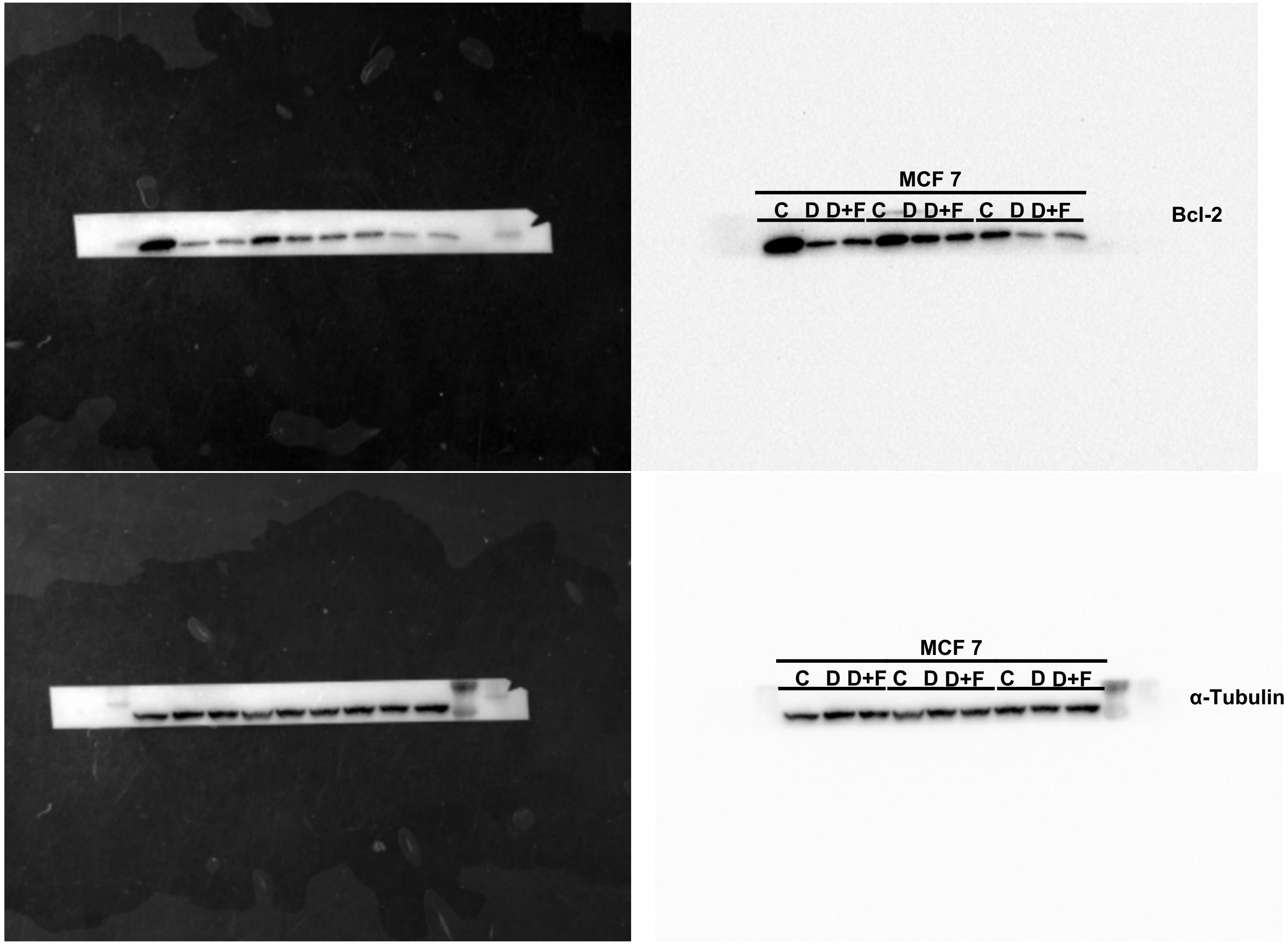

Bcl-2 D+F

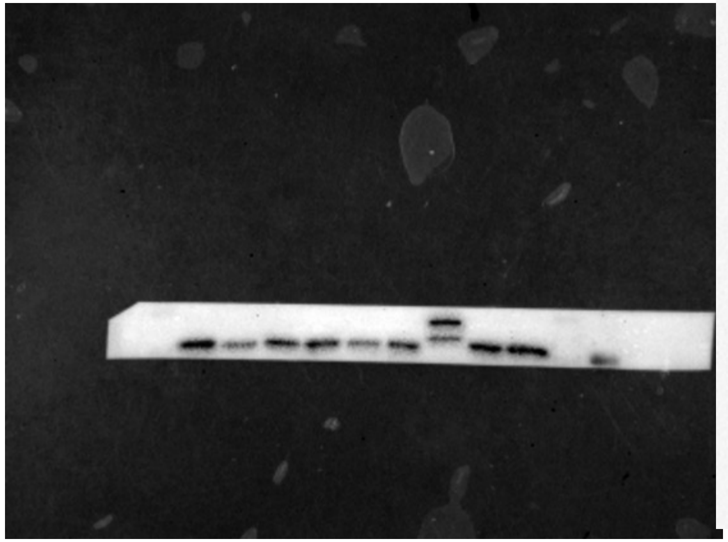

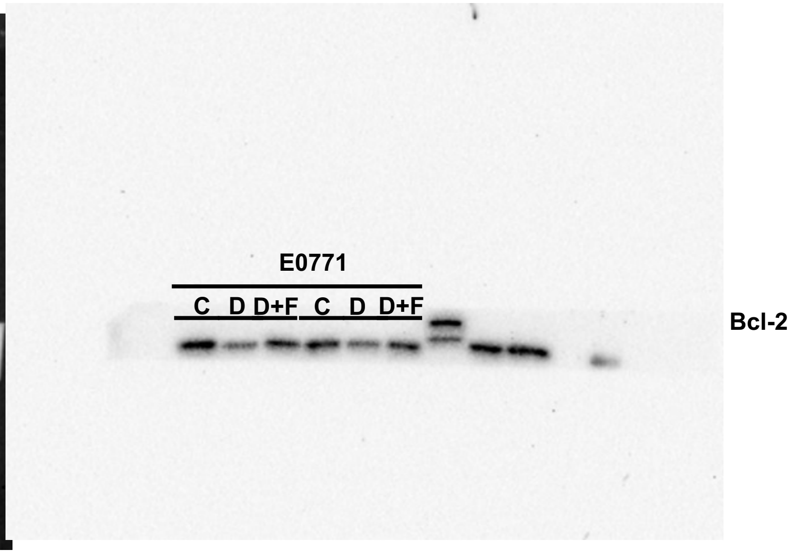

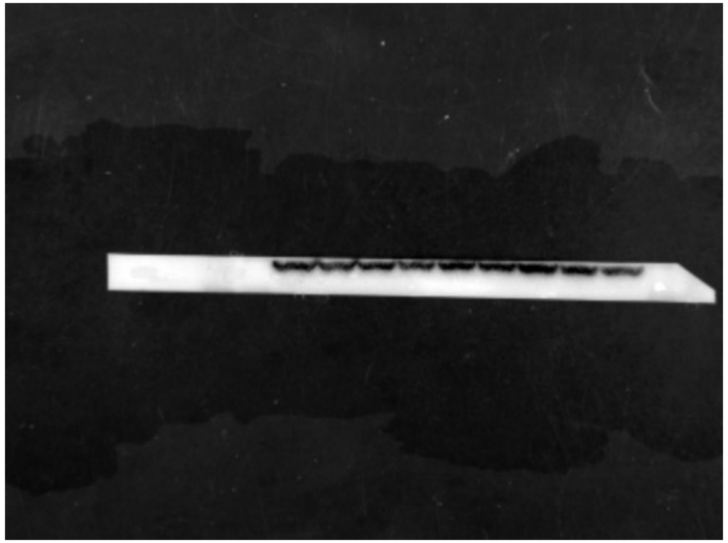

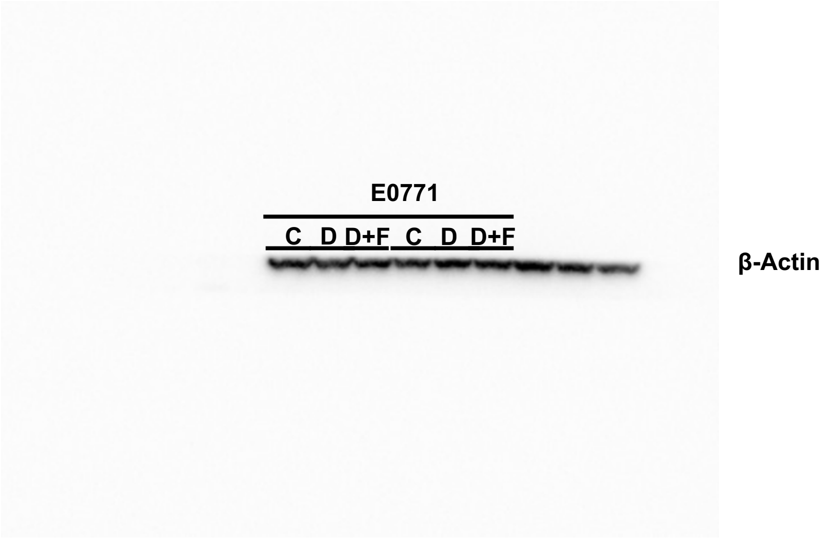

STAT3 D+F

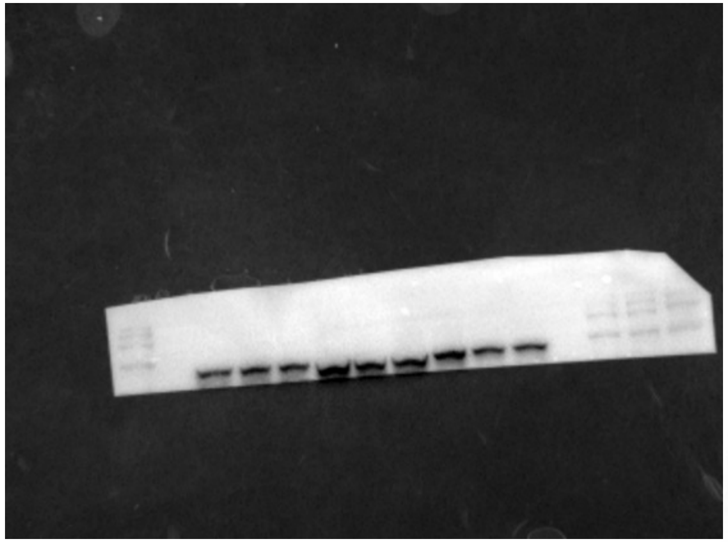

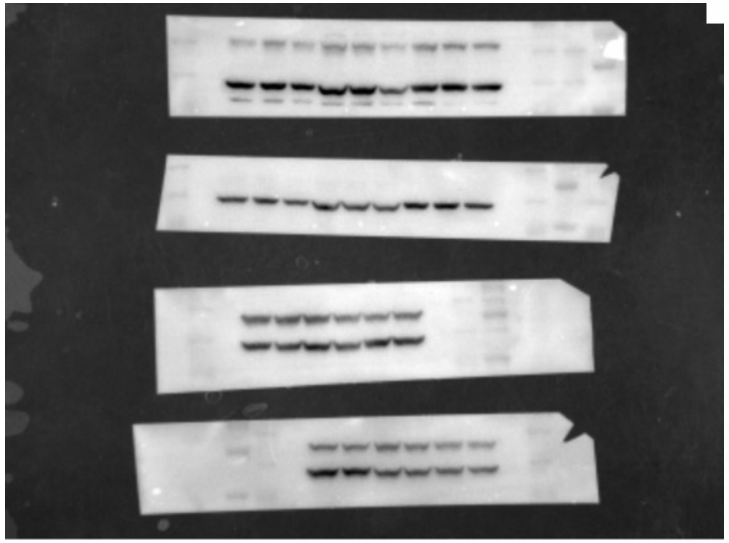

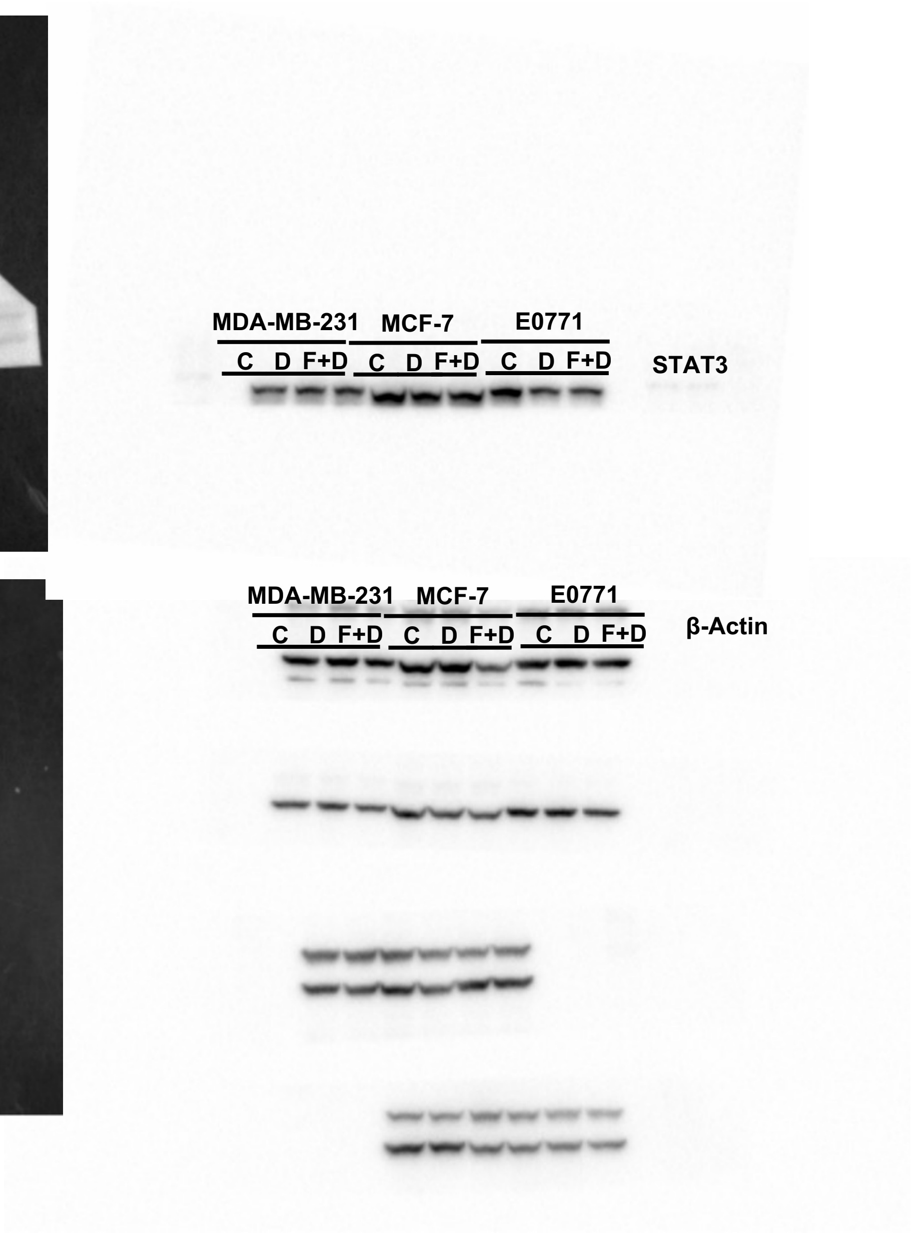
MDA-MB-231
MCF-7
E0771
C D F+D C D F+D C D F+D
STAT3
MDA-MB-231
MCF-7
E0771
C D F+D C D F+D C D F+D
β-Actin

STAT3 D+F

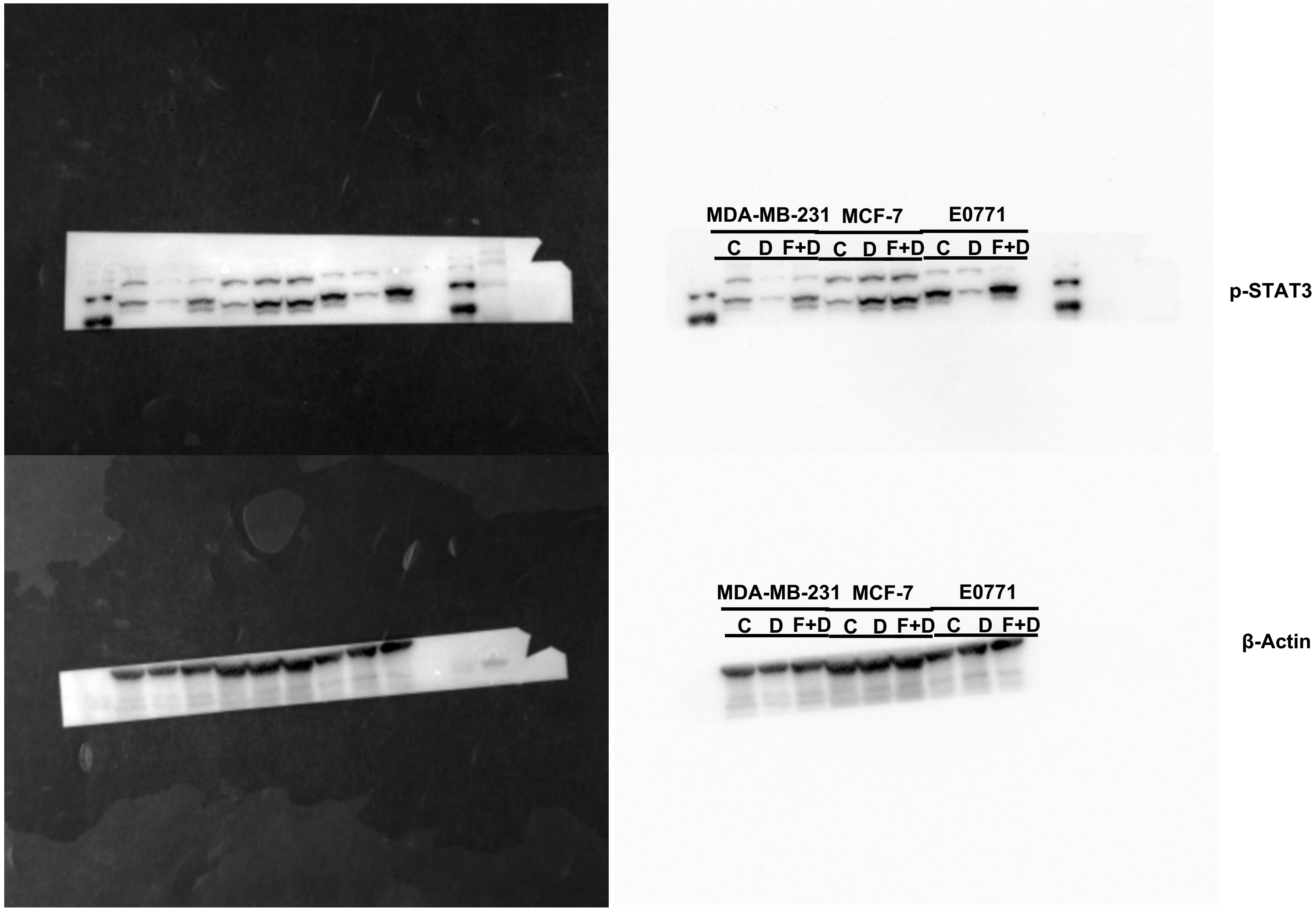
MDA-MB-231 MCF-7 E0771
C D F+D C D F+D C D F+D
p-STAT3
MDA-MB-231 MCF-7 E0771
C D F+D C D F+D C D F+D
β-Actin

Akt D+F

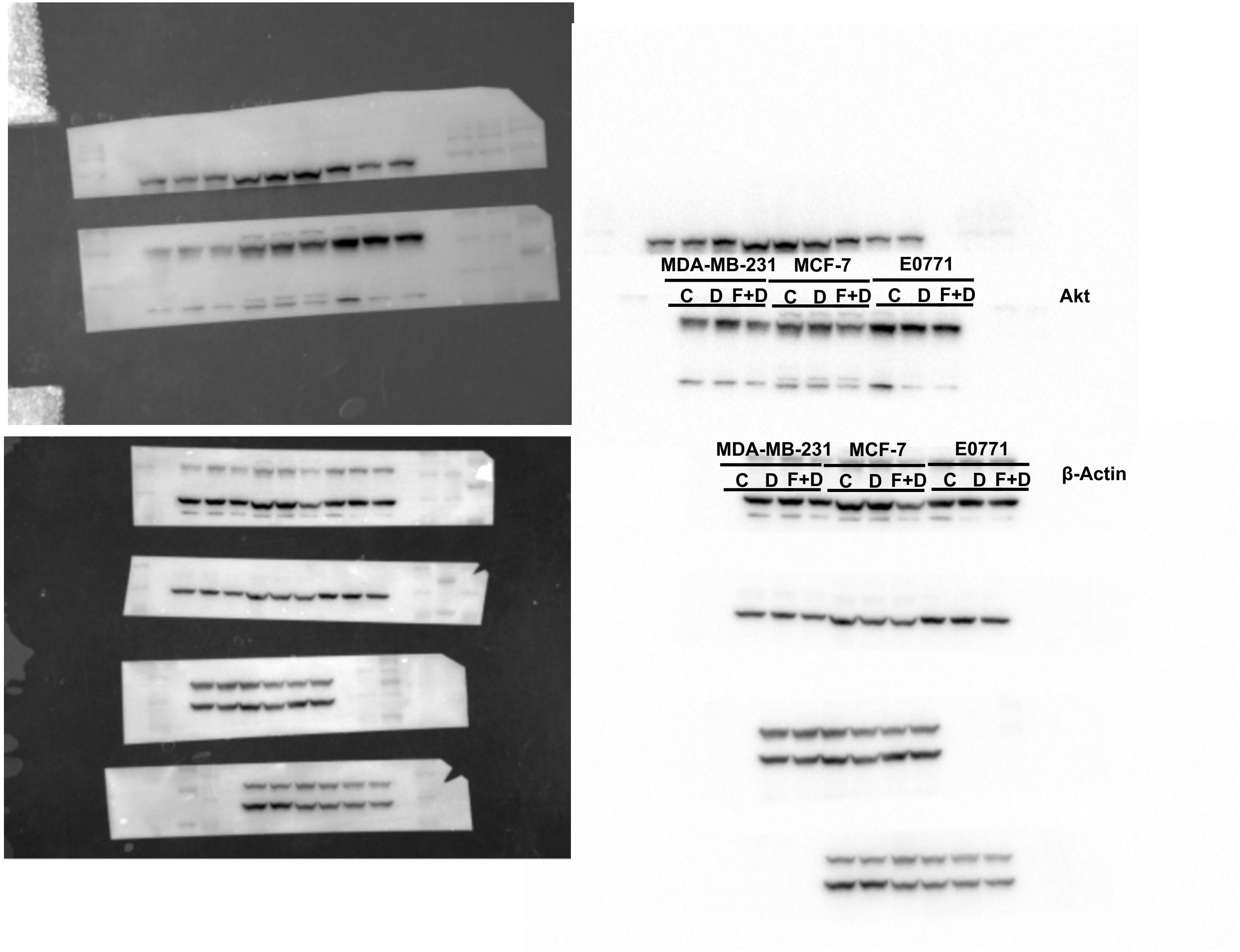
MDA-MB-231 MCF-7 E0771
C D F+D C D F+D C D F+D
Akt
MDA-MB-231 MCF-7 E0771
C D F+D C D F+D C D F+D
β-Actin

Akt D+F

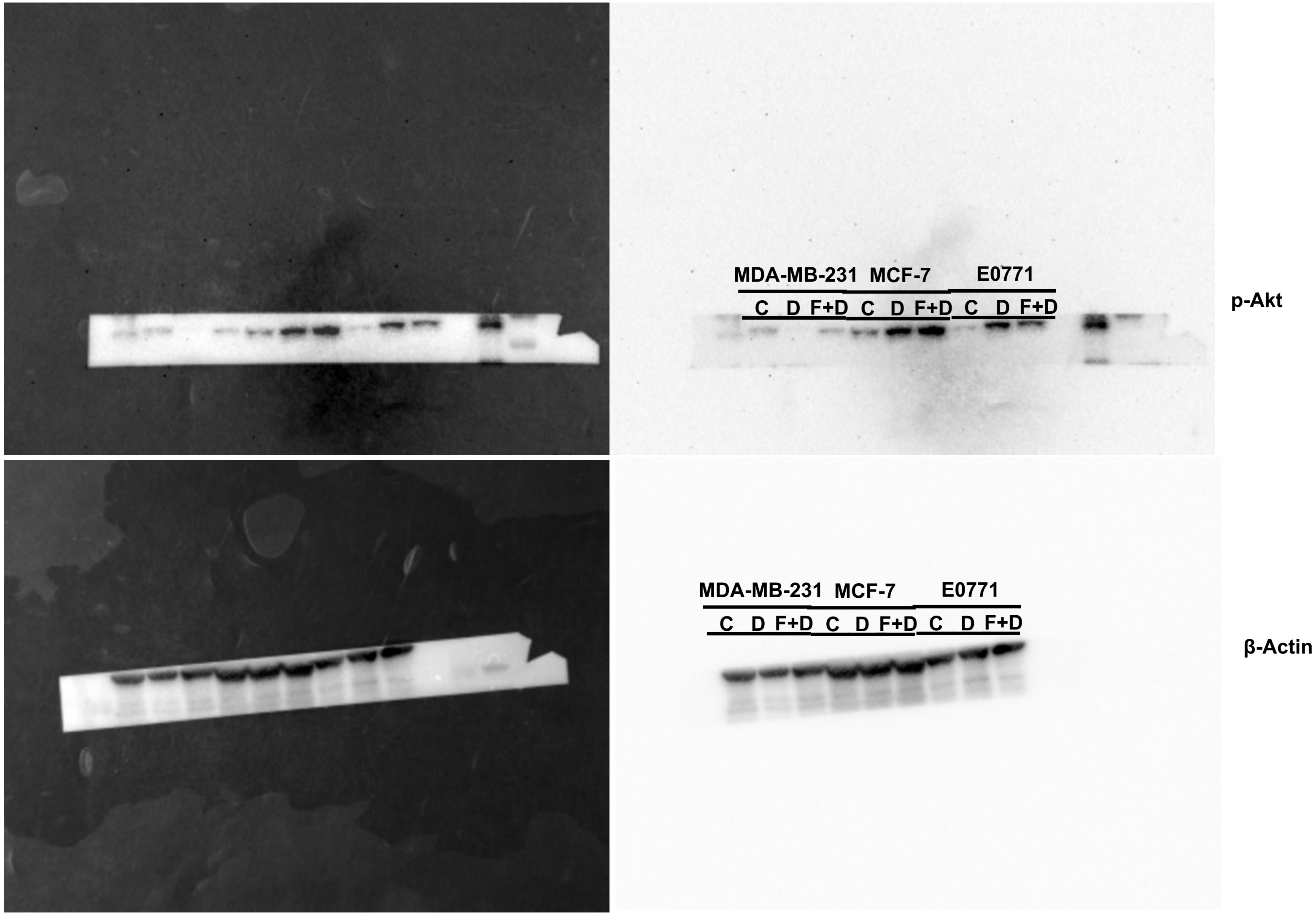
MDA-MB-231 MCF-7 E0771
C D F+D C D F+D C D F+D
p-Akt
MDA-MB-231 MCF-7 E0771
C D F+D C D F+D C D F+D
β-Actin

FoxO1 D+F

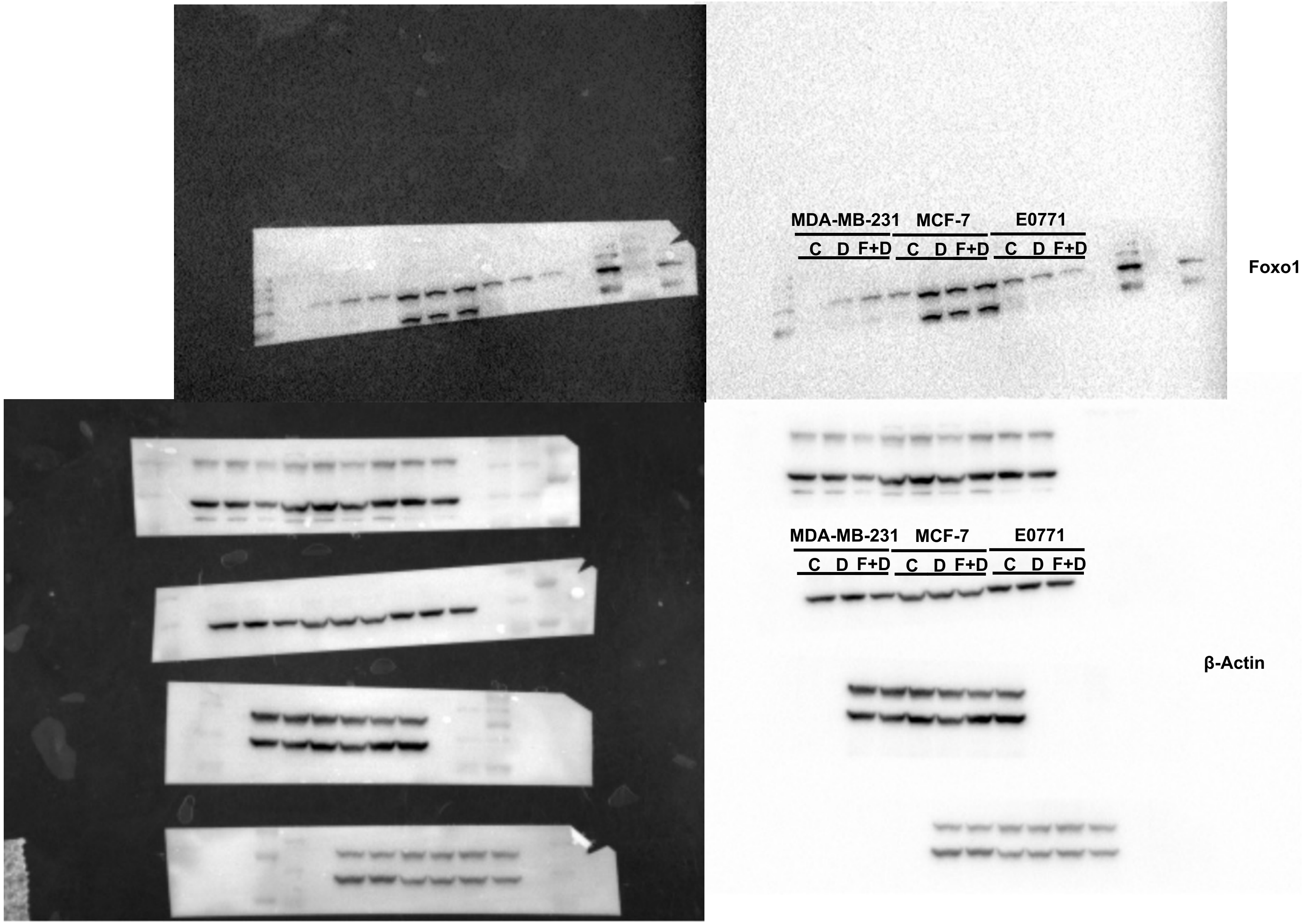
MDA-MB-231
MCF-7
E0771
C D F+D C D F+D C D F+D
Foxo1
MDA-MB-231
MCF-7
E0771
C D F+D C D F+D C D F+D
β-Actin

FoxO1 D+F

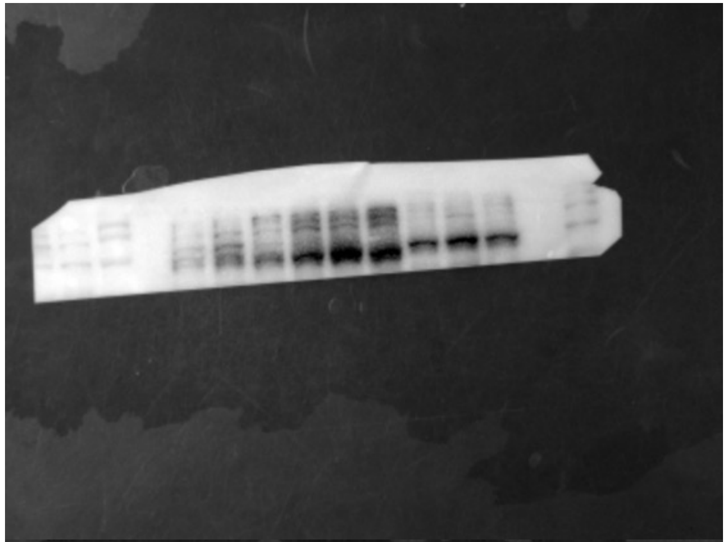

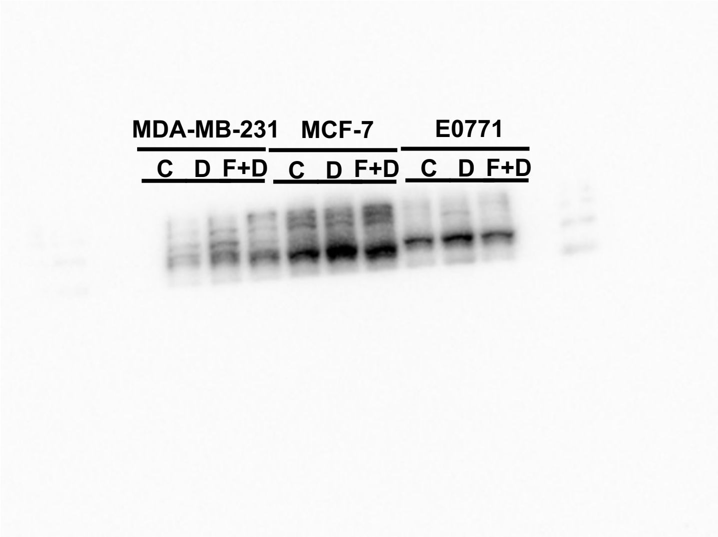

p-FoxO1

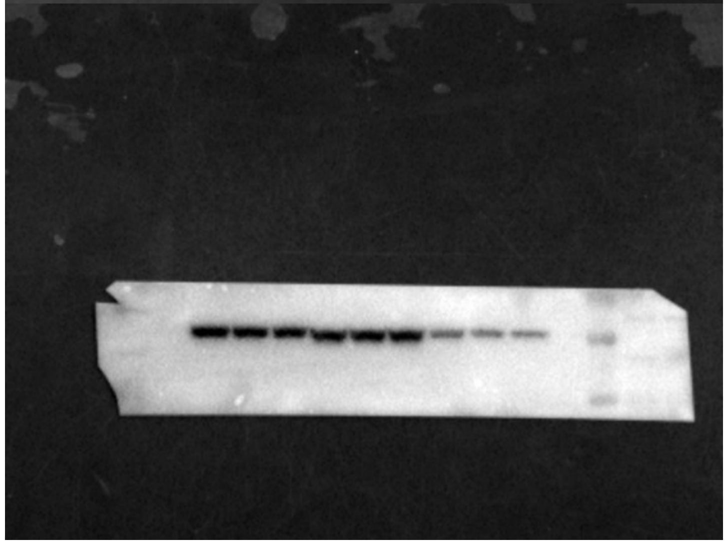

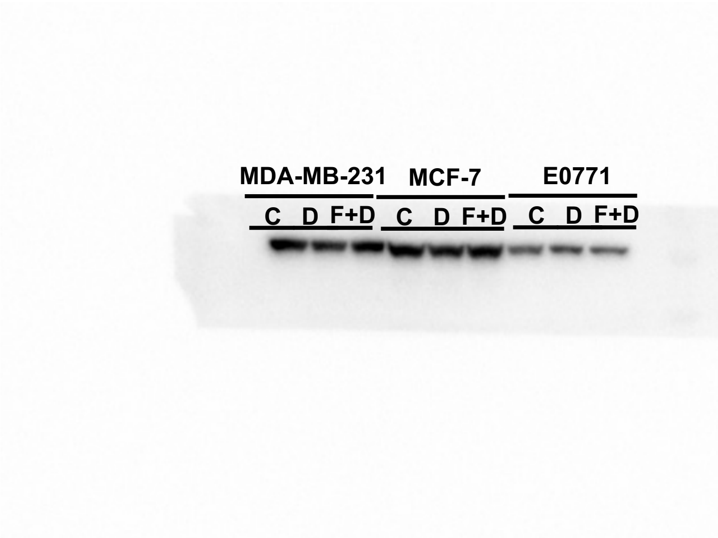

α-Tubulin
